# Key drivers of large scale changes in North Atlantic atmospheric and oceanic circulations and their predictability

**DOI:** 10.1007/s00382-025-07591-1

**Published:** 2025-02-05

**Authors:** Buwen Dong, Yevgeny Aksenov, Ioana Colfescu, Ben Harvey, Joël Hirschi, Simon Josey, Hua Lu, Jenny Mecking, Marilena Oltmanns, Scott Osprey, Jon Robson, Stefanie Rynders, Len Shaffrey, Bablu Sinha, Rowan Sutton, Antje Weisheimer

**Affiliations:** 1 National Centre for Atmospheric Science, Department of Meteorology, University of Reading, Reading, RG6 6BB UK; 2 National Oceanography Centre, Southampton, UK; 3 University of St Andrews, St Andrews, UK; 4 British Antarctic Survey, Cambridge, UK; 5 Atmosphere, Ocean and Planetary Physics, University of Oxford, Oxford, UK

**Keywords:** Atmospheric circulation, Oceanic circulation, Decadal changes, Anthropogenic forcings, North Atlantic climate system

## Abstract

Significant changes have occurred during the last few decades across the North Atlantic climate system, including in the atmosphere, ocean, and cryosphere. These large-scale changes play a vital role in shaping regional climate and extreme weather events across the UK and Western Europe. This review synthesizes the characteristics of observed large-scale changes in North Atlantic atmospheric and oceanic circulations during past decades, identifies the drivers and physical processes responsible for these changes, outlines projected changes due to anthropogenic warming, and discusses the predictability of these circulations. On multi-decadal time scales, internal variability, anthropogenic forcings (especially greenhouse gases), and natural forcings (such as solar variability and volcanic eruptions) are identified as key contributors to large-scale variability in North Atlantic atmospheric and oceanic circulations. However, there remain many uncertainties regarding the detailed characteristics of these various influences, and in some cases their relative importance. We therefore conclude that a better understanding of these drivers, and more accurate quantification of their relative roles, are crucial for more reliable decadal predictions and projections of regional climate for the North Atlantic and Europe.

**Supplementary Information:**

The online version contains supplementary material available at 10.1007/s00382-025-07591-1.

## Introduction

**Fig. 1:**
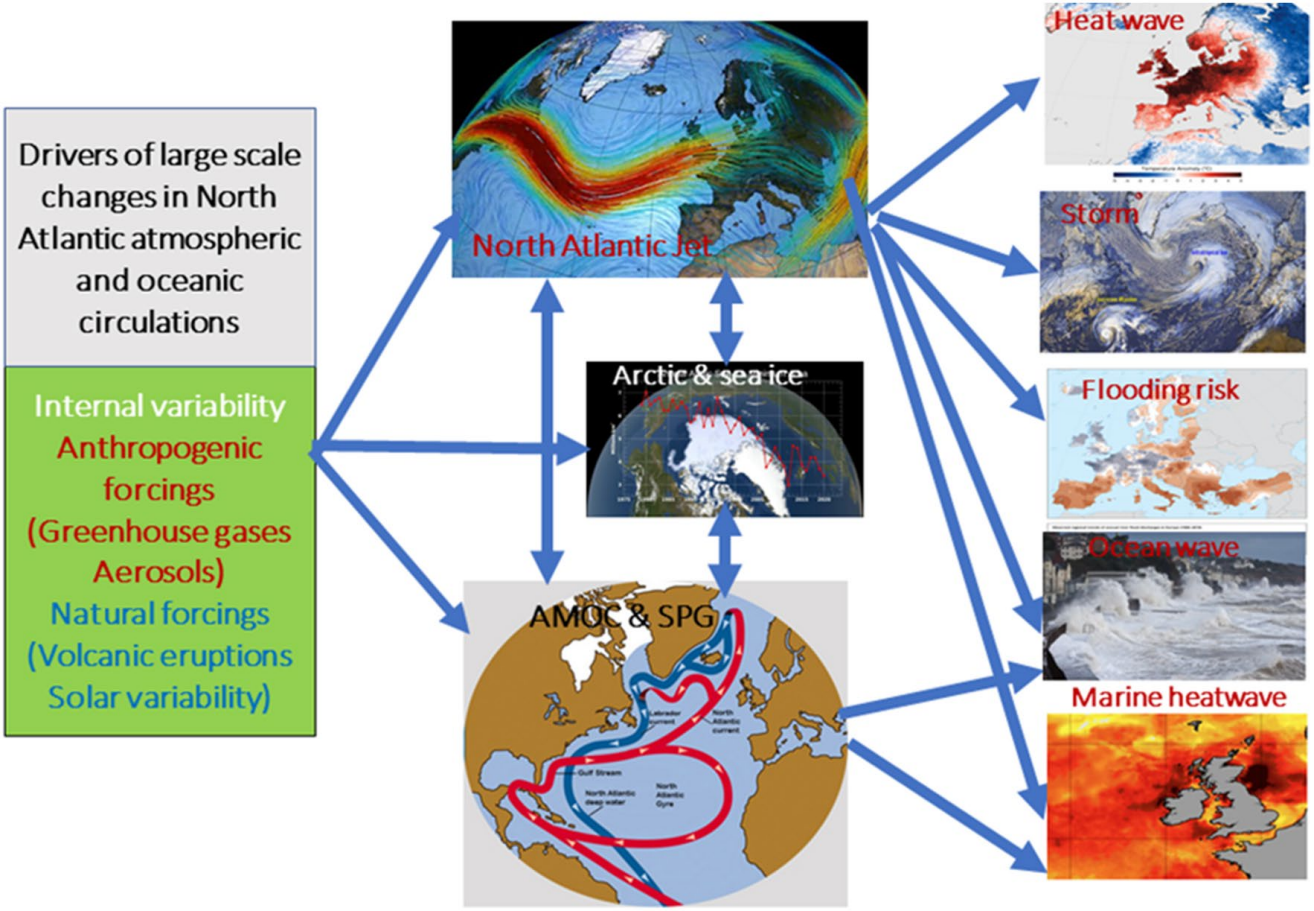
Schematic illustration of drivers (left box) of large scale changes in North Atlantic atmospheric and oceanic circulations (middle column) and associated impacts over the North Atlantic sector (right column). Images used in this figure are adapted from Crondallweather.co.uk, https://svs.gsfc.nasa.gov/5036, https://www.esa.int/SPECIALS/Eduspace_Weather_EN/SEM1HYK1YHH_1.html#subhead1, https://en.wikipedia.org/wiki/2006_European_heatwave, https://www.severe-weather.eu/mcd/north-atlantic-cyclone-windstorm-uk-mk/, https://www.eea.europa.eu/data-and-maps/figures/observed-regional-trends-of-annual, https://www.bbc.co.uk/news/science-environment-49731591, and https://news.stv.tv/scotland/seas-around-scotland-reach-unprecedented-record-breaking-temperatures-amid-extreme-marine-heatwave

Considerable changes have occurred during the last few decades across the North Atlantic climate system, in the atmosphere, ocean, and cryosphere (Sutton et al. 2017; Robson et al. 2018; Woollings et al. 2018). Of particular interest are changes in large-scale circulation patterns in the atmosphere and ocean, since they exert a major influence on regional climates across the North Atlantic sector, including on extreme events such as heatwaves, wind storms, floods, droughts, ocean surface waves and marine heatwaves (Fig. 1; Hurrell 1995; Coumou and Rahmstorf 2012; Dong et al. 2013; Hall et al. 2015; Deser et al. 2017; Iles and Hegerl 2017; Piecuch et al. 2019; Volkov et al. 2019; Rousi et al. 2021, 2022; Hallam et al. 2022; Dunstone et al. 2023; Schurer et al. 2023; Berthou et al. 2024; Simpson et al. 2024).

One of the most important features of atmospheric circulation over the North Atlantic is the eddy-driven jet, characterized by a band of strong westerly winds in the troposphere, with the maximum near tropopause, formed through westerly eddy momentum flux convergence associated with baroclinic eddies (Held 1975; Hoskins et al. 1983; Lorenz and Hartmann 2003; Barriopedro et al 2023). The North Atlantic jet exhibits variability on timescales from days to decades in both its position and intensity. This variability plays a vital role in shaping regional climate and extreme weather over the UK and western Europe (Woollings et al. 2010; Hall et al. 2015; Iles and Hegerl 2017; Simpson et al. 2019; Rousi et al. 2022; Teng et al. 2022) because synoptic scale disturbances tend to form in the regions of maximum jet stream wind speed, and to propagate eastward along tracks that follow the jet axis (Holton 1992; Hurrell 1995).

Observations suggest there has been significant low-frequency variability and/or trends in the North Atlantic jet in both winter and summer (Woollings et al. 2010, 2014, 2018, 2023; Hall et al. 2015; Hanna et al. 2015; Simpson et al. 2018; Dong and Sutton 2021; Hallam et al. 2022; Simmons et al. 2022). This variability has had large impacts on the UK and European climate (Hurrell 1995; Dong et al. 2013; Hanna et al. 2017; Iles and Hegerl 2017; Simpson et al. 2018, 2019; Rousi et al. 2021, 2022). However, the detailed mechanisms that govern low frequency variability of North Atlantic atmospheric circulation remain poorly understood.

Large-scale ocean circulation in the North Atlantic is dominated by the wind-driven gyres, i.e. the subpolar gyre (SPG) and subtropical gyre (STG), and the Atlantic Meridional Overturning Circulation (AMOC). Changes in the wind-driven gyres can impact the AMOC and vice versa (Hatun et al. 2005; Buckley and Marshall 2016). Observations, reanalyses, and proxies indicate substantial decadal-scale variability in the AMOC strength and abrupt changes in the SPG and STG (Robson et al. 2014; Buckley and Marshall 2016; Piecuch et al. 2019; Holliday et al. 2020; Desbruyères et al. 2021; Jackson et al. 2022; McCarthy et al. 2023).

Changes in AMOC strength can affect the climate in the Atlantic sector as well as in remote regions (e.g., Manabe and Stouffer 1999; Vellinga and Wood 2002; Zhang et al. 2019), such as shifts in the Intertropical Convergence Zone (ITCZ) (Vellinga and Wood 2002; Knight et al. 2006), regional sea-level (Levermann et al. 2005; Bingham and Hughes 2009; Little et al. 2019), Sahel/Asian summer monsoon rainfall (Folland et al. 1986; Dong et al. 2006; Knight et al. 2006), Atlantic hurricanes (Goldenberg et al. 2001; Hallam et al. 2019), summer climate over Europe and North America (Sutton and Hodson 2005; Sutton and Dong 2012), and tropical Pacific variability (Dong and Sutton 2007; Timmermann et al. 2007; Kucharski et al. 2016).

A wide range of drivers may influence decadal–multidecadal changes in large-scale North Atlantic atmospheric and oceanic circulations, as illustrated in Fig. 1. The large-scale changes could result from: (1) natural internal variability in the atmosphere (e.g., Wunsch 1999; Feldstein 2000; Eden and Willebrand 2001) and/or ocean (e.g., Sévellec and Fedorov 2013; Josey and Sinha 2022; Moat et al. 2024); (2) natural internal coupled ocean–atmosphere processes (Delworth et al. 1993; Dong and Sutton 2005; Omrani et al. 2014; Davini et al. 2015; Peings et al. 2016; Simpson et al. 2018; Lai et al. 2022); (3) responses to external anthropogenic forcings, such as anthropogenic greenhouse gas forcing (Gregory et al. 2005; Delworth and Dixon 2006; Graff and LaCasce 2012; Ceppi and Shepherd 2017; Harvey et al. 2020; Lee et al. 2021) and anthropogenic aerosol emissions (Delworth and Dixon 2006; Rotstayn et al. 2013; Bellomo et al. 2018; Shen and Ming 2018; Undorf et al. 2018a, b; Watanabe and Tatebe 2019; Menary et al. 2020; Dong et al. 2021, 2022; Hassan et al. 2021; Robson et al. 2022); and (4) responses to external natural forcings, such as the changes in solar ultraviolet radiation (Haigh 1996; Lockwood et al. 2010; Mignot et al. 2011; Menary et al. 2014; Gray et al. 2016; Lu et al. 2009, 2017a, b; Ye et al. 2023a) and volcanic eruptions (Swingedouw et al. 2017; Marshall et al. 2022; Paik et al. 2023).

In this paper, we review recent progress in characterizing and understanding decadal–multidecadal changes in North Atlantic atmospheric and oceanic circulations, and their associated impacts. More specifically, we first summarize: (1) major observed changes in the relevant atmosphere and ocean circulation patterns; (2) key drivers and physical processes involved in decadal–multidecadal changes in these circulation patterns, and to provide an overview of projected future changes; (3) evidence concerning the predictability of these circulation patterns. We then identify areas where further research is required.

This paper is structured as follows. We document the characteristics of observed decadal–multidecadal large-scale changes of atmospheric circulation in the North Atlantic sector during past decades, their drivers, projected changes in the future and physical processes in Sect. 2. Changes in oceanic circulation during past decades, projected changes, and responses to different forcing factors are presented in Sect. 3. The role of atmosphere–ocean coupling is described in Sect. 4, and evidence concerning multi-annual predictability and prediction is revealed in Sect. 5. Synthesis and discussions are presented in Sect. 6 and outstanding challenges in Sect. 7.

## Changes of large-scale atmospheric circulation in North Atlantic

### Observed changes

In recent decades, atmospheric circulation over the North Atlantic has exhibited substantial decadal–multidecadal change. Changes have been observed, for example, in both the latitude and speed of the North Atlantic eddy-driven jet in winter, summer and transition seasons (Woollings et al. 2014, 2023; Hall et al. 2015; Hallam et al. 2022; Simmons 2022). In this section we document key features of the observed changes.

#### Winter

**Fig. 2:**
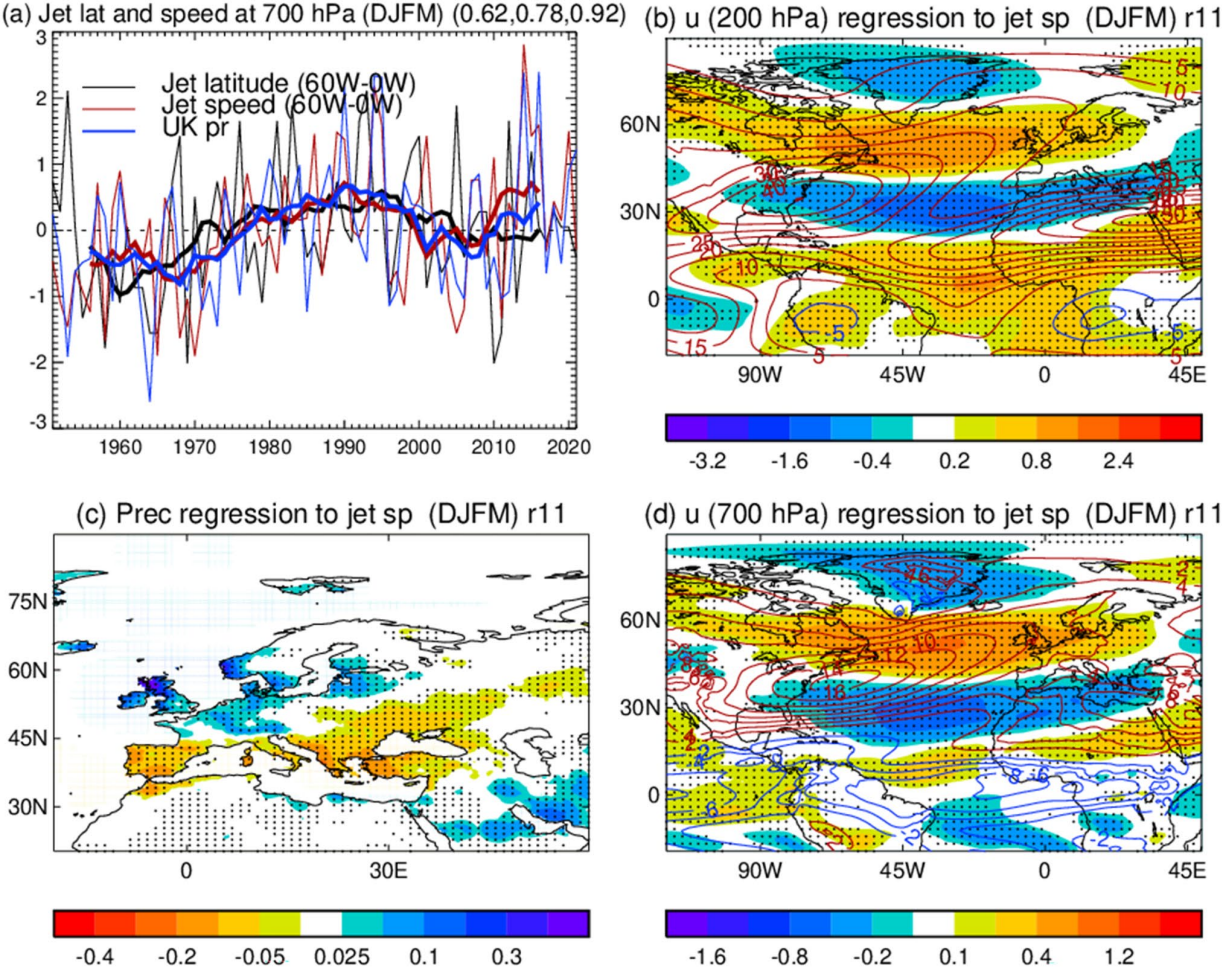
**a** Normalized (by standard deviation of interannual variability) time series of North Atlantic jet latitude and jet speed indices, based on ERA5 reanalysis (Hersbach et al. 2020) at 700 hPa, and UK precipitation index, based on CRUTS4.06 data set (Harris et al. 2014) in DJFM with thick lines representing low frequency (11 year running mean) variations. Jet latitude and jet speed indices are defined as the latitude of seasonal mean maximum zonal wind averaged over (60° W-0) and the corresponding maximum zonal wind speed at 700 hPa. The UK precipitation index is defined as the area averaged precipitation over the land region (51–59° N, 6° W-0). The three numbers in the bracket of the panel (**a**) are corelation coefficients among the jet latitude, jet speed, and precipitation indices (e.g., jet latitude vs jet speed, jet latitude vs precipitation, and jet speed vs precipitation). **b**, **c**, **d** Spatial patterns of 200 hPa zonal wind, precipitation, and 700 hPa zonal wind in DJFM regressed to the normalized low frequency variations of jet speed index in DJFM. Contours (**b** and **d**) show climatology and dots (**b**, **c**, **d**) highlight regions where regressions are statistically significant at the 10% level based on the two-tailed Student’s t test

Observations show that during 1951–2022 there was a strengthening of wind speed on the northward flank of the North Atlantic eddy-driven jet and in its downstream extension into northern Europe (Fig. 2 for extended winter DJFM, Supplementary Fig. S1 for DJF, Woollings et al. 2014; Simpson et al. 2019; Blackport and Fyfe 2022), and the strengthening is seen in both the lower and upper troposphere. Large decadal–multidecadal variations (11 year running mean) are characterized by a strengthening (~ 1.3 m s^−1^) and northward shift (~ 2.0 degree) of the jet from the 1960s to the 1990s, a weakening (~ 0.6 m s^−1^) and southward shift (~ 1.0 degree) from the 1990s to 2000s and recent strengthening (~ 0.5 m s^−1^) with little change in jet latitude. These low-frequency variations of the North Atlantic jet latitude and jet speed are positively correlated with each other (*r* = 0.62, *p* = 0.14) and the changes in jet speed are also closely related to low-frequency variability in the North Atlantic Oscillation (NAO), the seesaw pattern in atmospheric pressure between the Icelandic Low and the Azores High (Woollings et al. 2015). During the last few decades there has been a significant increase in the year-to-year variability of the winter NAO (Hanna et al. 2022), which is linked to more extreme UK winter weather at both ends of the spectrum (Hanna et al. 2017).

Increased jet speed (and northward displacement) is associated with anomalously wet conditions over the northern UK and northwestern Europe and dry conditions over southern Europe (Fig. 2; Simpson et al. 2019; Blackport and Fyfe 2022), and with anomalous warming over the mid-high latitudes in the northern hemisphere (Iles and Hegerl 2017). The correlation between jet speed and the UK precipitation is stronger in DJFM (*r* = 0.92, *p* < 0.01) than in DJF (*r* = 0.86, *p* = 0.01). This is consistent with Simpson et al. (2018, 2019) who identified pronounced multidecadal variability of the eddy-driven jet in March, which is significantly correlated to precipitation variability in western Europe.

#### Summer

**Fig. 3:**
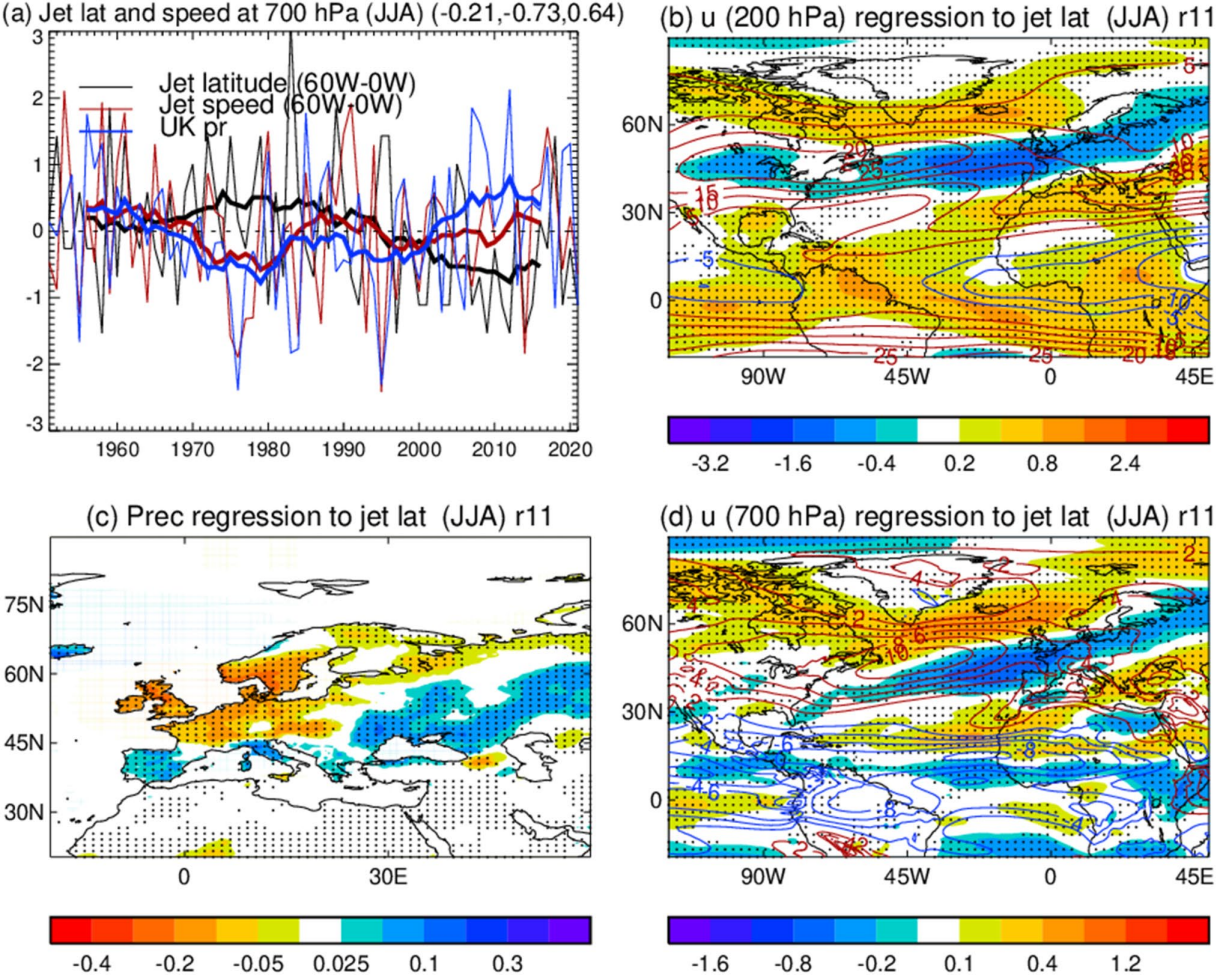
As Fig. 2, but for JJA. **b**, **c**, **d** Spatial patterns of 200 hPa zonal wind, precipitation, and 700 hPa zonal wind in JJA regressed to the normalized low frequency variations of jet latitude index in JJA

Observations also show notable multidecadal changes in the North Atlantic eddy-driven jet in summer during 1951–2022, characterized by a poleward migration of the jet latitude (~ 0.5 degree) and a decrease in jet speed (~ 0.3 m s^−1^) from 1950 to 1970s, followed by an equatorward migration (~ 1.0 degree) and an increase in the jet speed from 1980 to 2000s (~ 0.3 m s^−1^) (Fig. 3, Woollings et al. 2014; Dong et al. 2021; Simmons 2022). Since the early 2010s, a decrease in the jet speed and a northward displacement of the jet are evident. These low-frequency variations of the jet latitude and jet speed are negatively correlated in summer; i.e. a northward shift of the jet is associated with decreased jet speed. Also, the jet latitude index is negatively correlated with the UK rainfall (*r* = − 0.73, *p* = 0.06) while jet speed is positively correlated with the UK rainfall (*r* = 0.64, *p* = 0.13). Thus, a northward shift of the North Atlantic jet stream accompanied by decreased jet speed is associated with decreased rainfall over the UK and northwestern Europe but increased rainfall over the Iberian Peninsula and southeast Europe in summer (Fig. 3c, Supplementary Fig. S2c). A northward shift of summer North Atlantic jet is also associated with more frequent high temperature extremes, heatwaves and droughts over the UK and Europe (Dong et al. 2013, 2017; Iles and Hegerl 2017; Deng et al. 2022; Rousi et al. 2022).

#### Transition seasons

Research into North Atlantic circulation changes and their impacts on the UK and western European has mainly focused on the summer and winter seasons, with a few studies examining the transition seasons. In spring (March–May), the jet latitude shows a northward displacement from 1950 to 1990s and then a southward displacement from the mid-1990s onwards with weak change in the jet speed (Supplementary Fig. S3, Woollings et al. 2014). The low-frequency variability of the jet latitude and the UK precipitation are positively correlated with a correlation coefficient of 0.74 with the northward displacement being associated with increased rainfall. The northward displacement of spring jet is associated with enhanced precipitation over the UK and western Europe (Supplementary Fig. S3a, c). This is consistent with a recent study by Ionita et al. (2020) who showed that the recent southward displacement of the North Atlantic jet is associated with dry conditions over the UK and western Europe in spring (Ionita et al. 2020). However, the relationship of low-frequency changes between the jet speed and jet latitude is weak.

In autumn, the jet latitude index shows a northward displacement from 1950 to 1970s, a southward displacement from 1970s to mid-1990s and a northward displacement from the mid-1990s onwards (Supplementary Fig. S4a, Woollings et al. 2014). The low-frequency variability in jet speed and jet latitude are not well-correlated. The northward displacement of the North Atlantic jet is associated with decreased precipitation over the northern UK and western Europe and an increase in the frequency of drier summer-type circulation regimes (Vrac et al. 2014; Cotterill et al. 2023).

### Drivers

A range of drivers could be responsible for the observed decadal–multidecadal changes in the North Atlantic jet speed and jet latitude (Fig. 1, also see Simpson et al. 2018; Smith et al. 2022b). These drivers can be related to (1) natural internal variability in the atmosphere (e.g., Wunsch 1999; Feldstein 2000); (2) natural variability of coupled ocean–atmosphere processes (Buchan et al. 2014; Omrani et al. 2014; Davini et al. 2015; Duchez et al. 2016b; Peings et al. 2016; Simpson et al. 2018; Grist et al. 2019), (3) responses to external anthropogenic forcings, such as greenhouse gas forcing (Graff and LaCasce 2012; Ceppi and Shepherd 2017; Harvey et al. 2020; Lee et al. 2021) and aerosol emissions (Rotstayn et al. 2013; Shen and Ming 2018; Undorf et al. 2018a, b; Watanabe and Tatebe 2019; Dong et al. 2021, 2022), and (4) responses to external natural forcings, such as volcanic eruptions (Swingedouw et al. 2017; Marshall et al. 2022; Paik et al. 2023) and solar variability (Lockwood et al. 2010; Gray et al. 2016; Lu et al. 2017a, b; Drews et al. 2022; Kuroda et al. 2022). In this section, we summarize advances in understanding the influence of these drivers on decadal–multidecadal changes of the North Atlantic jet.

#### Internal variability

The North Atlantic jet varies naturally on timescales from weeks to decades and longer, resulting from intrinsic processes of the atmosphere that occur in the absence of external forcing (e.g., Wunsch 1999; Feldstein 2000). Low-frequency variability of the ocean is another factor that may contribute to changes in the characteristics of the jet (Simpson et al. 2018). Studies have suggested that multidecadal variability of sea surface temperature (SST) over the North Atlantic (Atlantic multidecadal variability, AMV) may influence the jet through a stratospheric pathway (Kerr 2000; Omrani et al. 2014), from the tropical Atlantic (Davini et al. 2015; Peings et al. 2016) or via a local influence of the North Atlantic SSTs on stationary waves, baroclinicity, and/or eddy–mean flow interactions (Kushnir 1994; Msadek et al. 2011; Gastineau and Frankignoul 2012; Peings et al. 2016).

Some studies have found that the response of atmospheric circulation to local North Atlantic SST anomalies is small (Kushnir et al. 2002; Thomson and Vallis 2018a, b). But this conclusion has mostly been drawn from model-based analyses. Given their apparent deficiencies e.g., the systematic underestimation of North Atlantic atmospheric circulation variability on multi-decadal timescales in coupled climate models (O’Reilly et al. 2021), it is possible that the real-world atmosphere exhibits a greater response to extratropical SST anomalies. There is growing evidence that the atmospheric response to midlatitude SST anomalies might be systematically underestimated in climate models (Scaife and Smith 2018; Simpson et al. 2018, 2019; Czaja et al. 2019; Wills et al. 2019). Recent studies have found that models with improved representation of ocean fronts and the overlying atmosphere could significantly alter the nature of ocean–atmosphere coupling (Smirnov et al. 2015; Siqueira and Kirtman 2016; Parfitt et al. 2017; Famooss Paolini et al. 2022; Seo et al. 2023).

#### Anthropogenic forcings

##### The response to greenhouse gas forcing

In response to an increase in anthropogenic greenhouse gases (GHG) concentrations, the troposphere is expected to warm with the maximum warming in the tropical upper troposphere (Meehl et al. 2007; Santer et al. 2008) and at the surface in polar regions especially over the Arctic (Holland and Bitz 2003; Screen and Simmonds 2010). In contrast, the stratosphere is expected to cool globally (Shine et al. 2003). This non-uniform response pattern modifies both horizontal and vertical atmospheric temperature gradients, with expected impacts on the mid-latitude atmospheric baroclinicity and therefore mid-latitude atmospheric circulation (Graff and LaCasce 2012; Shaw et al. 2016; Ceppi and Shepherd 2017; Shaw 2019; Harvey et al. 2020; Lee et al. 2021). The warming in the upper troposphere of the tropics and cooling in the stratosphere, and enhanced warming at the surface of the Arctic, have been shown to force opposite responses in the jet location and strength (e.g., Held 1993; Harvey et al. 2014, 2015; Shaw et al. 2016; Shaw 2019; Stendel et al. 2021).

In association with the tropospheric warming and stratospheric cooling in response to GHG increases, the midlatitude jet streams and storm tracks are predicted to shift poleward under future climate warming (e.g., Yin 2005; Lorenz and DeWeaver 2007; Lu et al. 2008; Kidston and Gerber 2010; Barnes and Polvani 2013; Shaw et al. 2016; Simpson and Polvani 2016; Mbengue and Schneider 2017; Shaw 2019), accompanied by an expansion of the Hadley cell (e.g., Hu et al. 2013; Nguyen et al. 2015; Tao et al. 2016; Grise and Davis 2020). However, enhanced warming at the Arctic surface drives an equatorward shift in the mean jet location by decreasing the surface meridional temperature gradient, and thus baroclinic eddies that maintain the North Atlantic eddy-driven jet (Butler et al. 2010; Screen et al. 2013; Shaw 2019; Chen et al. 2020; Stendel et al. 2021). Current consensus across climate models is that the upper-tropospheric warming wins out over the Arctic surface warming, causing a net poleward shift of the zonal mean jet in winter and annual mean (Yin 2005; Barnes and Polvani 2013; Harvey et al. 2014; Shaw 2019). However, there is still substantial disagreement among models over the magnitude of the jet response (Manzini et al. 2014; Grise and Polvani 2016; Peings et al. 2018; Harvey et al. 2020, 2023).

The above results focused on zonal mean changes, and there is important regional variability of the jet stream and associated storm track responses (Woollings and Blackburn 2012; Barnes and Polvani 2013; Oudar et al. 2020). For example, studies based on CMIP3 model simulations indicated that the winter North Atlantic jet will strengthen and extend eastward, with corresponding stormtrack changes (Bengtsson et al. 2006, 2009; Ulbrich et al. 2008; Shaw et al. 2016). A similar but slightly weaker response is found in CMIP5 and CMIP6 models (Haarsma et al. 2013; Peings et al. 2018; Harvey et al. 2020, 2023).

The changes in North Atlantic jet stream in summer in response to GHG forcing, generally show a northward shift with weak changes in jet speed in the multimodel mean (Barnes and Polvani 2013; Harvey et al. 2020, 2023). However, there is a large spread in the jet response in both winter and summer among different models (Barnes and Polvani 2013; Manzini et al. 2014; Harvey et al. 2020, 2023), which promotes the value of jet-based storylines for assessing and communicating uncertainty in local climate projections over the UK and western Europe (Zappa and Shepherd 2017; Harvey et al. 2023).

##### The response to anthropogenic aerosol forcing

Anthropogenic aerosols affect global and regional climate through aerosol-radiation and aerosol-cloud interactions (e.g., Boucher et al. 2013). Because of their inhomogeneous spatial distributions, anthropogenic aerosols (AAer) or aerosol precursor emissions can cause changes in horizontal and vertical temperature gradients, which in turn affect atmospheric circulation (Rotstayn et al. 2013; Shen and Ming 2018; Undorf et al. 2018a), potentially including the strength and position of the Northern Hemisphere subtropical jet stream (Undorf et al. 2018a; Dong and Sutton 2021; Dong et al. 2022). Anthropogenic aerosols may affect atmospheric circulation directly through fast responses of the atmosphere and land-surface and also more slowly through aerosol-induced changes in sea surface temperatures (SSTs), such as a potential influence on the AMV (Booth et al. 2012; Undorf et al. 2018a, b; Watanabe and Tatebe 2019). For instance, it has been found that anthropogenic sulphate emissions explain 46–63% of the forced SST variations at decadal time scales (Watanabe and Tatebe 2019).

Pausata et al. (2015a) showed that AAer reductions in the near future caused a more positive winter NAO by using an atmospheric general circulation model that is coupled with a mixed layer ocean model. The positive winter NAO is accompanied by an eastward shift of the Azores High with a significant increase in blocking frequency over the western Mediterranean. AAer reductions may also result in the AMV being increasingly controlled by internal variability with reduced impact on regional precipitation (Watanabe and Tatebe 2019). Dong and Sutton (2021) suggested that AAer changes may have contributed to an observed equatorward trend in the North Atlantic summer jet from the 1970s to 2010s (see also Undorf et al. 2018a) and the impact of AAer changes on meridional temperature gradients in the lower troposphere is implicated as a key mechanism.

#### Natural forcings

Multi-decadal changes in the eddy-driven jet over the North Atlantic has been found to be sensitive to external natural forcings, such as solar variability (Lockwood et al. 2010; Gray et al. 2010; Drews et al. 2022) and volcanic eruptions (Swingedouw et al. 2017; Marshall et al. 2022; Paik et al. 2023). The stratospheric processes and the associated coupling with the troposphere during winter and spring plays a part in linking North Atlantic variability with those external natural forcings.

Changes in the absorption of solar ultraviolet (UV) radiation by stratospheric ozone over the 11-year solar cycle has the potential to organise/synchronise the decadal variation of the NAO whereby the strength and positioning of the North Atlantic westerly jet are modulated (Gray et al. 2016; Drews et al. 2022). Lockwood et al. (2010) revealed a statistical link between low solar activity and cold European winters, consistent with Barriopedro et al. (2008) who found blocking episodes in the East Atlantic increased in both duration and intensity when solar activity was low. During solar maxima, the effect is significantly projected onto a positive NAO, thus a poleward shift of the North Atlantic jet (Gray et al. 2016; Drews et al. 2022). A lagged positive NAO response by around three years to solar maximum has also been found (Gray et al. 2013; Scaife et al. 2013). The response appears to be induced by changes in the meridional temperature gradient of the upper stratosphere, subsequent poleward and downward propagation of stratospheric wind anomalies via modulation of stratospheric polar vortex and air-sea interactions that accumulate solar influences to favour a poleward and eastward migration of the North Atlantic jet (Ineson et al. 2011; Gray et al. 2016; Drews et al. 2022). The response to solar activity may also be conditional upon the phases of other forcings, such as the Quasi-Biennial Oscillation (QBO) in the tropical lower stratosphere (e.g., Lu et al. 2009). The solar signal may be further modified/amplified by other processes, including nonlinear planetary wave breaking, downward wave reflection, and resonance with the impact being on the seasonal evolution rather than the mean state (Lu et al. 2017a, b). A recent study based on ensemble simulations of a chemistry-climate coupled model suggests that solar influence on the jet shift is non-stationary and mainly associated with the solar cycles that have larger-than-average amplitude (Drews et al. 2022). Yet other model-based studies found no systematic solar influence on the North Atlantic and western European climate (Chiodo et al. 2019; Osman et al. 2021). To date, a complete process understanding of solar influence and its interaction with internal variability and other drivers remains inconclusive.

Observations suggest that large tropical volcanic eruptions may induce a poleward shift of the North Atlantic jet stream in winter (Robock and Mao 1995; Robock 2000). Volcanic eruptions inject a large amount of aerosol particles into the atmosphere, which scatter incoming shortwave radiation while absorbing/emitting longwave radiation, causing cooling of the Earth's surface but warming of the stratosphere (Stenchikov et al 2009). The enhanced meridional temperature gradient in the lower stratosphere may result in a positive phase of the NAO via a strengthening of the stratospheric polar vortex, thus a strengthened and poleward shifted jet stream over the North Atlantic in winter that lasts up to 5 years (Robock and Mao 1995; Marshall et al. 2009). Contrary to low-latitude eruptions, the response to high-latitude eruptions can be associated with a negative NAO both in winter and summer (Sjolte et al. 2021).

This effect has so far been reproduced by only a few CMIP5 and CMIP6 models (Charlton-Perez et al. 2013; Polvani and Camargo 2020; Paik et al. 2023). It seems that climate models can reproduce a pattern similar to the observed NAO response in the first winter following the eruption, but the amplitude of the response and impact on surface temperatures are rather weak in comparison with observations (Hermanson et al. 2020). Although stratospheric warming tends to shift the jet poleward, uncertainty remains in terms of the response to surface cooling which may interface with the stratosphere-related eddy circulation feedbacks and their connection to intrinsic natural variability (DallaSanta et al. 2019).

#### Arctic changes and its influences on the North Atlantic

In recent decades, the warming in the Arctic has been much faster than in the rest of the world in both observations and climate models, a phenomenon known as Arctic amplification (AA) (Serreze et al. 2009; Rantanen et al. 2022). The question to what extent Arctic amplification and related sea ice loss may impact mid-latitude weather and general atmospheric dynamics has received a lot of attention over the past decades (e.g., Francis and Vavrus 2012; Cohen et al. 2014, 2020; Barnes and Screen 2015; Overland et al. 2015; Screen 2017; Screen et al. 2013, 2018, 2022, Zappa et al. 2018; Ye et al. 2023b). Separate hypotheses have been proposed to explain the link between amplified Arctic warming and mid-latitude atmospheric circulation in winter and summer and they involve changes in the polar vortex, storm tracks, jet stream, planetary waves, stratosphere-troposphere coupling, and eddy-mean flow interactions (e.g., Doblas-Reyes et al. 2021).

In winter, it has been proposed that amplified warming of the Arctic, which weakens the meridional temperature gradient in the lower troposphere and weakens the predominant westerly wind, could cause the northern hemisphere jet stream to shift equatorward and result in a weaker jet and larger-amplitude waves (e.g., “wavier” circulation) in the midlatitude circulation (Francis et al. 2017). A wavier circulation has been proposed to link to increased occurrence of extreme midlatitude weather (e.g., Francis and Vavrus 2012; Barnes and Screen 2015; Screen 2017; Cohen et al. 2018; Riboldi et al. 2020; Riebold et al. 2023).

In summer, it has been proposed that amplified warming of the Arctic could result in a weakening of the westerly jet and mid-latitude storm tracks, as suggested for the recent period of Arctic warming (Coumou et al. 2015; Petrie et al. 2015; Chang et al. 2016). It is hypothesized that weaker jets, diminished meridional temperature contrast, and reduced baroclinicity might induce a larger amplitude in stationary wave response to stationary forcings (Zappa et al. 2011; Hoskins and Woollings 2015; Coumou et al. 2018; Mann et al. 2018), and also that a double jet structure would favour wave resonance (Kornhuber et al. 2017; Mann et al. 2018), that is associated with simultaneous heatwaves in the northern hemisphere (Coumou et al. 2014; Kornhuber et al. 2019; Teng et al. 2022).

Studies that support the Arctic influence are mostly based on observational relationships between the Arctic temperature or sea ice extent and mid-latitude anomalies or extremes (Cohen et al. 2012; Francis and Vavrus 2012, 2015; Budikova et al. 2017). However, climate models are unable to simulate significant responses to Arctic sea ice loss, larger than the natural variability (Screen 2017, England et al. 2018; Screen et al. 2018; Peings et al. 2017; Blackport and Screen 2020; Dai and Song 2020; Smith et al. 2022a). These divergent conclusions between model and observational studies, and also between different model studies continue to obfuscate a clear understanding of how Arctic warming is influencing mid-latitude weather (Blackport and Screen 2020; Cohen et al. 2020; Dai and Song 2020; Overland et al. 2021). The 6th Assessment Report (AR6) from the intergovernmental Panel on Climate Change (IPCC) concluded “there is low confidence in the relative contribution of Arctic warming to mid-latitude atmospheric changes compared to other drivers” (Doblas-Reyes et al. 2021).

### Attribution of observed changes

A common approach to examine which drivers are responsible for the observed variability (Sect. 2.1) is to analyse ensembles of free-running coupled climate model simulations including all external forcings (natural and anthropogenic), or a subset of them. The simulations should, as a minimum, exhibit variability consistent with that observed. In that case, an attempt can be made to attribute to one or both of external forcings (if the variability is present in the ensemble mean) or to internal variability (if it is only exhibited by some members). If external forcings are important then single forcing experiments can be used to further explore the most important forcing. If, however, the simulations do not exhibit variability consistent with the observations, then the reasons for the discrepancy must be examined to gain confidence in the model simulations.

One important change in observations in winter was a tendency toward the positive phase of the winter NAO and a strengthening and northward shift of the jet stream from the 1960s to the 1990s (Fig. 2, Hurrell 1995), weakening and southward shift from the 1990s to 2000s and recent strengthening in the jet speed with weak change in the jet latitude (Fig. 2, Blackport and Fyfe 2022). However, climate models struggled to capture these observed decadal–multidecadal variations (Osborn 2004; Eyrin et al. 2021; Blackport and Fyfe 2022; Eade et al. 2022; Shaw et al. 2024). Blackport and Fyfe (2022) showed that over the period from 1951 to 2020, the wintertime North Atlantic jet has strengthened, while model trends are, on average, only very weakly positive. The observed strengthening is greater than in any one of the ensemble simulations from CMIP6 climate models considered. Climate models also have biases in many key aspects of the North Atlantic circulation, including the eddy driven jet in winter that is displaced to the south and east in many models (Harvey et al. 2020), and underestimates of blocking frequency over the northeast Atlantic and Europe (Dunn-Sigouin and Son 2013). Such biases can affect model response to external forcings and can compound difficulties in detecting and attributing trends (Dong and Sutton 2021).

The weak response to external forcings in the models has been attributed to a number of reasons, including poor representation of tropical-extratropical and stratosphere-troposphere coupling (O’Reilly et al. 2019; Klavans et al. 2021; Shaw et al. 2024), lack of persistence in surface temperature in particular over oceans (Sévellec and Drijfhout 2019), underestimation of regime behaviour (Strommen and Palmer 2019), lack of eddy feedbacks (Scaife et al. 2019; Blackport and Fyfe 2022; Hardiman et al. 2022), and errors in ocean–atmosphere coupling (Zhang et al. 2021), as the paradox seems to be common to coupled models (e.g., Scaife and Smith 2018).

Observations show notable multidecadal changes in the summer jet during 1951–2022, characterized by poleward migration and decreasing jet speed from 1950 to 1970s, followed by an equatorward migration and increasing jet speed from 1980s. This shift is related to increase in Greenland atmospheric blocking over the same time period (Hanna et al. 2016). However, multimodel mean of CMIP6 model simulations failed to capture these observed decadal–multidecadal changes of atmospheric circulation in the North Atlantic (Harvey et al. 2023). There is limited evidence that specific features of atmospheric circulation in the North Atlantic region in summer have been affected by changes in anthropogenic aerosol (AAer) emissions (Undorf et al. 2018a, b; Dong and Sutton 2021). However, the causes of southward displacement of the summer North Atlantic jet from the 1980s are still poorly understood (Dong and Sutton 2021).

### Projected changes

**Fig. 4:**
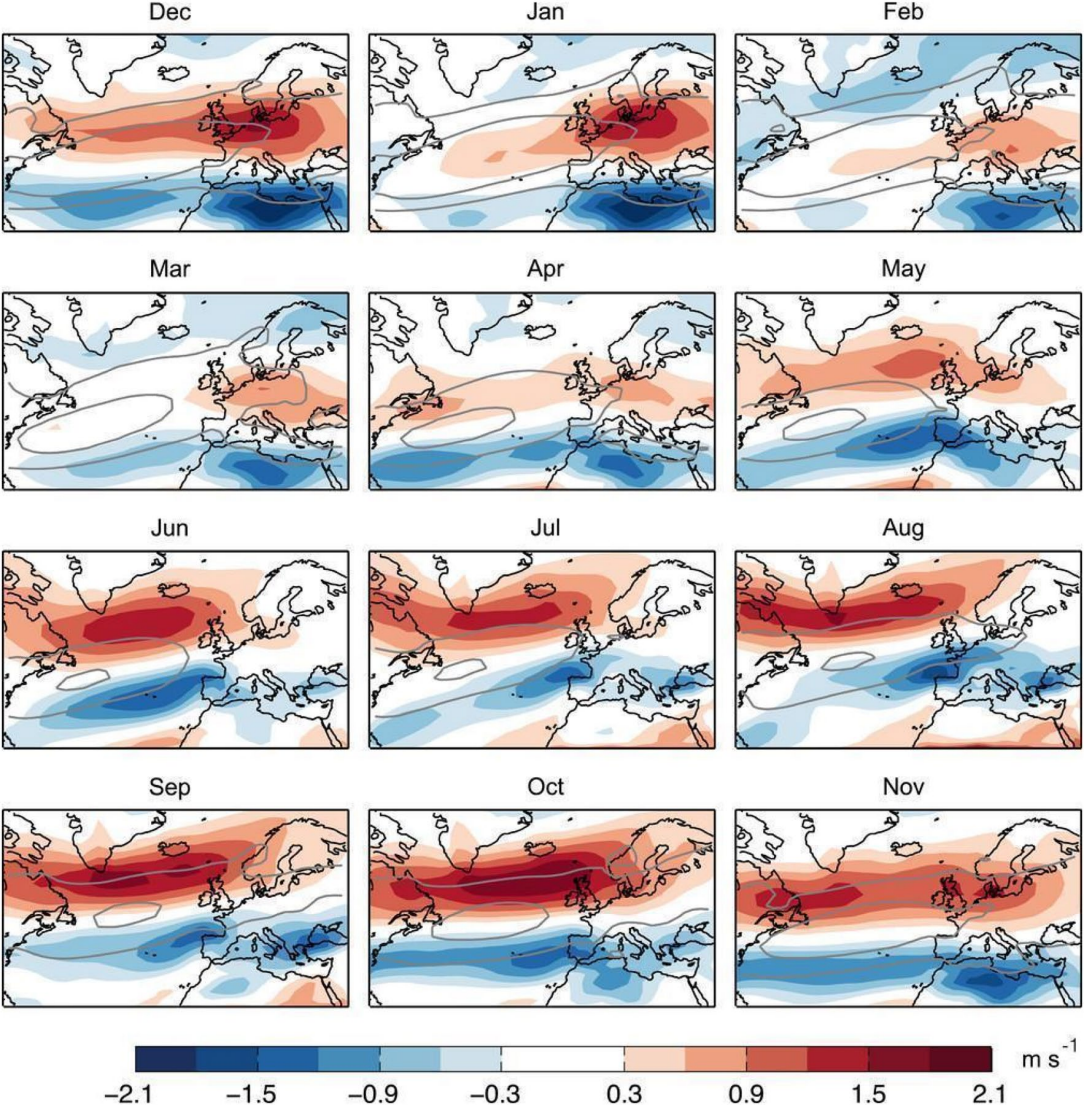
CMIP5 multimodel mean climate response (shading) in the zonal wind at 850 hPa by the end of the twenty-first century under the RCP8.5 scenario. The climate response is separately presented for each individual calendar month. Grey contours correspond to the 4 (outer) and 8 (inner) m s^−1^ isotachs of the zonal wind at 850 hPa in the historical period (1960–90) in the multimodel mean. This image is adapted from Fig. 1 of Zappa et al. (2015) 10.1175/JCLI-D-14-00823.1. ^©^ American Meteorological Society. Used with permission

**Fig. 5:**
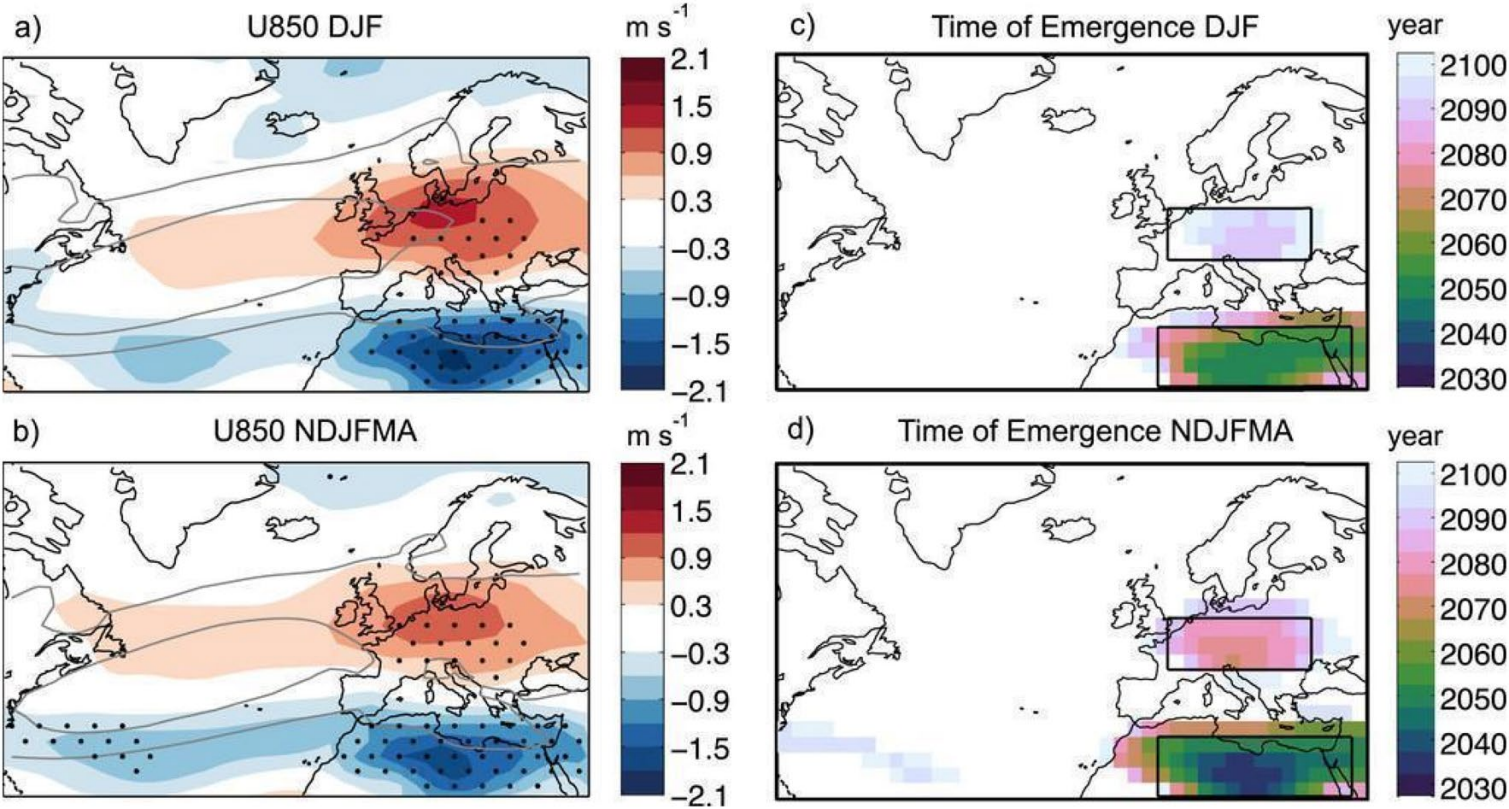
Multimodel mean end-of-century U850 response separately computed for the **a** meteorological winter (DJF) and **b** extended winter (NDJFMA) time averages. **c**, **d** The time of emergence of the U850 response evaluated for the time periods in (**a**) and (**b**), respectively. In (**a**) and (**b**), stippling is applied where at least 90% of the models show a response of the same sign for the end-of-century climate change response, and the grey contours correspond to the 4 (outer) and 8 (inner) m s^−1^ isotachs of U850 in the historical period in the multimodel mean. This image is adapted from Fig. 3 of Zappa et al. (2015) 10.1175/JCLI-D-14–00823.1. © American Meteorological Society. Used with permission

Analyses of CMIP5 and CMIP6 multimodel mean response in zonal wind show a strong seasonal and regional dependence in the response to climate change of westerlies in the Northern Hemisphere. Seasonal differences of the North Atlantic jet response by the end of the twenty-first century are readily apparent, with the maximum northward jet shift occurring in autumn (~ 1.5 degree) and no clear shift in winter (Fig. 4; Woollings and Blackburn 2012; Barnes and Polvani 2013; Simpson et al. 2014; Harvey et al. 2020; Zhou et al. 2022). Furthermore, the shift of the westerlies in the North Atlantic in winter is uncertain and the responses in individual models differ considerably from the multimodel mean and from each other (Simpson et al. 2014; Zappa and Shepherd 2017; McKenna and Maycock 2021). Projected changes in the North Atlantic jet tend to be squeezed on both its equatorward and poleward flanks and to be strengthened in the core, together with an eastward extension into Europe (Fig. 5; Peings et al. 2018; Harvey et al. 2020, 2023; Oudar et al. 2020), indicating an enhanced jet speed at the jet exit region and downstream extension of the North Atlantic jet. This feature is more pronounced and the time of emergence is earlier in the extended winter season (NDJFMA) than in DJF (Fig. 5). This may lead to increased seasonal mean precipitation and increased risk of flooding over the UK and western Europe in winter (Rousi et al. 2021; Harvey et al. 2023).

**Fig. 6:**
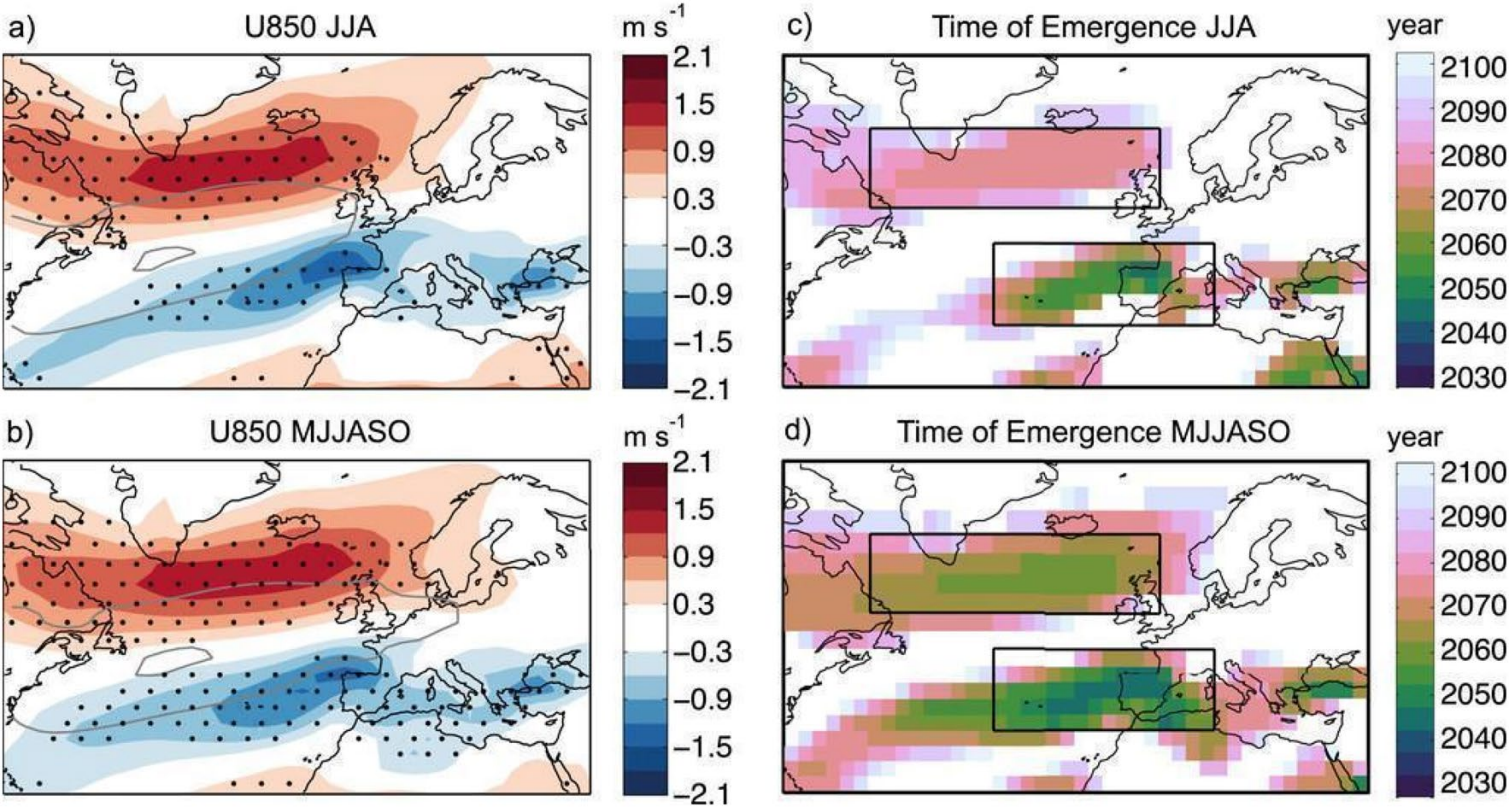
Multimodel mean end-of-century U850 response separately computed for the **a** meteorological summer (JJA) and **b** extended summer (MJJASO) time averages. **c**, **d** The time of emergence of the U850 response evaluated for the time periods in (**a**) and (**b**), respectively. In (**a**) and (**b**), stippling is applied where at least 90% of the models show a response of the same sign for the end-of-century climate change response, and the grey contours correspond to the 4 (outer) and 8 (inner) m s^−1^ isotachs of U850 in the historical period in the multimodel mean. This image is adapted from Fig. 2 of Zappa et al. (2015) 10.1175/JCLI-D-14-00823.1. ^©^ American Meteorological Society. Used with permission

Recent studies have shown some robust patterns emerging regarding future changes of westerlies in the lower troposphere over the North Atlantic sector with a notably northward shift of jet in summer by ~ 1.2 degree (Fig. 6; Simpson et al. 2014; Zappa et al. 2015; Harvey et al. 2020, 2023; Lee et al. 2021), which is consistent with the expected future drying of the UK and western Europe and exacerbated hot and dry extremes over the twenty-first century (Rousi et al. 2021; Herrera‐Lormendez et al. 2023).

During the transition seasons, climate model projections based on CMIP5 simulations show a poleward shift by about 0.8 and 1.5 degrees in the westerlies over the North Atlantic in spring and autumn, with no robust change in the jet speed (Barnes and Polvani 2013; Simpson et al. 2014), being consistent with a projected increase in the frequency of drier summer-type circulation regimes over the UK and western Europe in autumn (Cotterill et al. 2023).

## Changes of ocean circulation in North Atlantic

### Observed changes

Identification of historical changes to the basin scale North Atlantic Ocean circulation, principally the AMOC and the subtropical and subpolar gyres (STG and SPG respectively), is hampered by a paucity of measurements. Since the 1950s we have had reasonably good coverage of upper ocean temperature and salinity, much improved since the early 2000s with the deployment of the Argo float array, from which we can calculate upper ocean geostrophic transports. However, uncertainties in the choice of reference level of no motion remained until the advent of satellite based observations of absolute sea surface height from the early 1990s onwards. Direct measurements of the meridional overturning were not available until the deployment of trans-basin mooring arrays from the early 2000s. In addition to direct observations, there are three other potential sources of information about circulation changes: numerical model based state estimates which attempt to minimise observation-model mismatch; free running ocean models forced by observation based surface meteorological conditions; and observation based proxies which are thought to correlate with ocean circulation, evidenced by a combination of theory and model- and observation-based verification.

#### Changes of AMOC

**Fig. 7:**
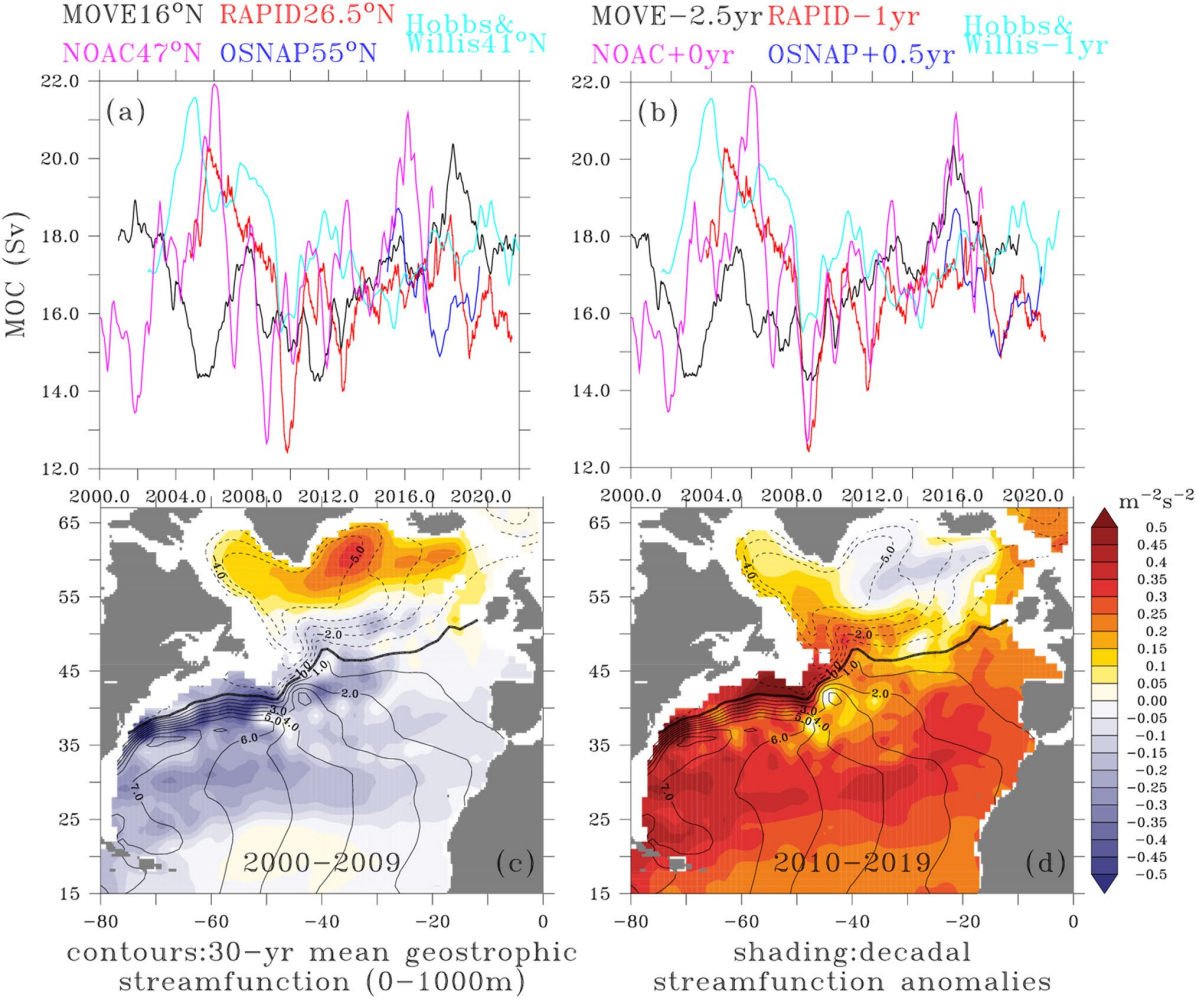
Synthesis of observed North Atlantic Ocean circulation changes since the year 2000 **a** North Atlantic MOC timeseries (Sv) as estimated at ~ 16 N (MOVE array, black line), ~ 26.5 N (RAPID/MOCHA array, red), ~ 41 N (Hobbs/Willis, cyan), ~ 47 N (N OAC, magenta), OSNAP (~ 55 N, blue). A 12 month running mean filter has been applied to each timeseries to remove subannual variations. **b** as **a** with the timeseries shifted temporally to maximise coherence between them. **c** contours represent absolute geostrophic streamfunction (dynamic metres) averaged over 0–1000 m depth and over years 1993–2021 based on the EN4 optimally interpolated temperature-salinity dataset (1 degree latitude–longitude grid) and sea surface height observed via satellite altimetry (CMEMS). Shading represents streamfunction anomalies (relative to 1993–2021) averaged over the decade 2000–2009. **d** as **c** except shading represents anomalies over 2010–2019

Our most reliable information about AMOC changes comes from synthesising estimates from the trans-basin monitoring arrays (Fig. 7a, see also Volkov et al. 2023). Here we focus on five major efforts. Going from north to south we have the OSNAP array (Lozier et al. 2019) at approximately 55° N, the NOAC array at 47° N (Wett et al. 2023), the Hobbs/Willis estimate at 41° N (Hobbs and Willis 2012), the RAPID/MOCHA array at approximately 26.5° N (Moat et al. 2020), and the MOVE array at 16° N (Kanzow et al. 2006; MOVE is not trans-basin but useful for our purposes as it monitors the transport of the main deep southward branch of the AMOC).

Figure 7 shows that there is a remarkable degree of coherence between the AMOC timeseries on multi-year to decadal timescales. Leaving aside the short OSNAP record for the moment, a rising trend in the AMOC from 2000 to about 2006 is well attested by RAPID, Hobbs/Willis and NOAC as is a subsequent downward trend until 2009–2010 (however continuing to about 2014 in the MOVE array). From the minimum at 2009–2010 RAPID, Hobbs/Willis and NOAC all show a rising trend, punctuated by interannual excursions (which are not always coherent across latitudes) until the 2020s. The MOVE array seems out of phase with the other three timeseries, showing a declining trend from 2000 to 2005 when the AMOC is rising at other latitudes and then a rising trend to 2008 when the AMOC is falling at other latitudes. However, from 2011 onwards, the AMOC at MOVE rises in a similar way to the other timeseries. Whilst the OSNAP timeseries is still too short to draw robust conclusions about decadal trends, it does show a reduction in overturning strength between 2015 and 2018 followed by a slight rise in subsequent years, opposite to the general rising trend of the other timeseries.

Interestingly, the coherence between all five timeseries can be dramatically improved by applying temporal shifts to the data (Fig. 7b). Remarkably, the size of the temporal shift depends monotonically on the latitude, with larger delays required at lower latitudes, being consistent with the idea that AMOC anomalies propagate along the western boundary (Zhang 2008; Polo et al. 2014; Ortega 2021). Overall, changes occurring at OSNAP seem to be communicated to MOVE on a three year timescale. Ocean model hindcasts (e.g., Megann et al. 2021), surface forced overturning circulation measures (Josey et al. 2009), state estimates and observation based proxies generally support the observations and further suggest that the changes seen in the trans-basin arrays are part of a longer term variability consisting of an increasing trend from 1980 to the mid-1990s and a decline since then, with the peak occurring a few years earlier in subpolar latitudes compared to subtropical (Jackson et al. 2022).

#### Changes in the horizontal circulation

To explore changes in North Atlantic upper ocean circulation, we take advantage of the availability of three decades of concurrent satellite derived absolute sea surface height above geoid coupled with good quality gridded in situ temperature and salinity data to determine absolute pressure as a function of depth. Restricting ourselves to water depths less than 1000 m where the data is most reliable, we plot the absolute pressure divided by a reference density averaged over 0–1000 m depth to provide an indication of a streamfunction for the upper ocean horizontal transport (contours, Fig. 7c, d). The separated Gulf Stream and the North Atlantic Current (NAC) are well represented, with the zero contour (thick black) connecting Cape Hatteras on the US east coast with the coast of the island of Ireland off western Europe. The circulation in the clockwise SPG is indicated by the dashed contours to the north and the solid contours to the south indicate the STG recirculation. The shading on the figures indicates changes to this baseline transport during 2000–2009 (Fig. 7c) and 2010–2019 (Fig. 7d) to cover the decreasing/increasing AMOC regimes pre- and post-2010. During 2000–2009 there was a significant decrease in pressure all along the Gulf Stream and NAC resulting in a weakened cross-stream gradient and along stream transport relative to the 30 year mean. Simultaneously there was increased pressure over the subpolar North Atlantic indicating a weaker circulation in SPG, with the largest changes occurring in the Irminger Sea. 2010–2019 saw a reversal of these anomalies, with increased Gulf Stream/NAC and STG transport, in the latter case, with a centre of action in the eastern part of the gyre. Interestingly, anomalously positive pressure anomalies persisted over both periods in the western gyre.

The results presented here are consistent with a number of recent studies investigating sea surface height and steric height variability in the SPG and its relationship with the horizontal circulation. Chafik et al. (2019) analyzed steric and dynamic sea level trends to reveal a relatively weak SPG during the mid-1990s with a transition to a stronger gyre circulation since the late 2000s. Notably, the gyre contracts in longitudinal extent as it gets stronger, associated with a northward shift of the North Atlantic current and warmer conditions in the eastern subpolar North Atlantic. The strengthening of the gyre was found to be associated with a shift to stronger cyclonic windstress curl over its central part. Desbruyères et al. (2021) documented a more recent warming trend in the upper SPG since 2016 and used a variety of analysis techniques to infer that this has been associated with a weakening and contraction of the gyre continuing until the present.

#### Relationship between changes in AMOC and horizontal circulation

The question of the dynamical and thermodynamic links between AMOC changes and horizontal gyre changes remains much debated. The relatively strong subpolar gyre in the early 1990s (Chafik et al. 2019) was coincident with a strengthening AMOC, itself brought about by persistent positive NAO related buoyancy forcing from higher precipitation and surface ocean warming (Robson et al. 2012), whilst the warming and weakening of the SPG in the late 1990s and early 2000s coincided with a weakening AMOC related to a decline in deep convection in the Labrador Sea (Fig. 7c, Robson et al. 2012, 2016) also demonstrated that subpolar gyre strength in the 1990s and 2000s was related to buoyancy forcing (as opposed to wind forcing) associated with the NAO, although the mechanism behind this is still unclear.

### Drivers

There are different drivers that may influence decadal–multidecadal changes in large scale oceanic circulations over the North Atlantic (Fig. 1). These include: (1) natural internal variability in the atmosphere (e.g., Wunsch 1999; Feldstein 2000; Eden and Willebrand 2001) and ocean (Sévellec and Fedorov 2013; Moat et al. 2024), (2) internal coupled ocean–atmosphere processes (Delworth et al. 1993; Dong and Sutton 2005; Omrani et al. 2014; Ortega et al. 2015; Lai et al. 2022), (3) responses to external forcings, such as anthropogenic forcings in greenhouse gas forcing (Gregory et al. 2005; Delworth and Dixon 2006; Graff and LaCasce 2012; Lee et al. 2021) and aerosol emissions (Delworth and Dixon 2006; Bellomo et al. 2018; Menary et al. 2020; Hassan et al. 2021; Robson et al. 2022), and (4) responses to external natural forcings, such as the changes of solar radiation (Mignot et al. 2011; Menary et al. 2014; Ye et al. 2023a) and volcanic eruptions (Swingedouw et al. 2017; Marshall et al. 2022; Paik et al. 2023).

#### Internal Variability

Ocean-only experiments suggest that decadal–multidecadal AMOC variability in the North Atlantic primarily results from buoyancy forcing over subpolar regions (Eden and Willebrand 2001; Marshall et al. 2001; Böning et al. 2006; Robson et al. 2012; Yeager and Danabasoglu 2014). Many previous coupled atmosphere–ocean model simulations with constant forcings have also exhibited substantial multidecadal variations in AMOC and have associated this variability with the internal interactions of ocean currents or with coupled interactions between different components of the coupled system (e.g., ice, ocean, and atmosphere) in the North Atlantic region (Delworth et al. 1993; Vellinga and Wu 2004; Dong and Sutton 2005; Jungclaus et al. 2005; Park and Latif 2008; Delworth and Zeng 2012; Menary et al. 2015; Ortega et al. 2015; Wills et al. 2019; Jiang et al. 2021; Jackson et al. 2022; Lai et al. 2022; Meccia et al. 2023). In many of the studies the propagation of freshwater from the Arctic (e.g., Jungclaus et al. 2005; Jiang et al. 2021) or salinity anomalies from the south (Delworth et al. 1993; Vellinga and Wu 2004), and the dominant timescales are set by advective processes, such as the spin-up/spin-down of the North Atlantic SPG circulation or the accumulation of high-/low-density water in deep water formation regions. However, many studies also indicate an important role for atmospheric circulation changes due to ocean–atmosphere coupling (e.g., Omrani et al. 2022; Lai et al. 2022). Some studies also suggest that Rossby wave adjustment, with little or no influence of the atmosphere, can be the dominant driver of decadal timescale AMOC variability, especially within the subpolar latitudes (Sevelec and Fedorov 2013; Muir and Fedorov 2017).

#### Anthropogenic forcings

##### The response to greenhouse gas forcing

There is general agreement that increasing concentrations of greenhouse gases act to weaken the AMOC in climate models (Gregory et al. 2005; Delworth and Dixon 2006; Stouffer et al. 2006; Caesar et al. 2018; Thornalley et al. 2018; Menary et al. 2020; Eyring et al. 2021). Weakening of the AMOC under greenhouse gas forcing results from both reduced heat loss to the atmosphere and increasing freshwater fluxes at high latitudes, both leading to lighter surface waters, which in turn may lead to a reduction of deep convection in sinking regions and thus impact the strength of the AMOC (e.g., Gregory et al. 2005; Manabe and Stouffer 1999; Stouffer et al. 2006; Eyring et al. 2021). Advection of heat and salinity anomalies into the North Atlantic deep convection region can also affect the AMOC. For instance, studies have also attributed a weakening of the AMOC in GCMs to Arctic sea ice loss (Sévellec et al. 2017) and subsurface warming of the North Atlantic (Haskins et al. 2020; Levang and Schmitt 2020), which both increase ocean stratification and inhibit deep convection. However, the amount, the rate and the effects of this decline are highly uncertain across models (Gregory et al. 2005; Collins et al. 2013; Weijer et al. 2020; Bellomo et al. 2021; Gulev et al. 2021; Lee et al. 2021; Fox-Kemper et al. 2021). The response to decreasing greenhouse gas concentrations has recently been examined (Schwinger et al. 2022). Overshoot scenarios show reduction of AMOC and subsequent recovery (see Sect. 3.4.2). However, the AMOC response depends strongly on peak GHG concentrations and the rate at which they are then removed.

##### The response to aerosol forcing

Many studies have highlighted that AAer forcing can have a large impact on the North Atlantic, and AAer forcing has been shown to strengthen the AMOC in climate models (Cai et al. 2006; Delworth and Dixon 2006; Undorf et al. 2018a, b; Andrews et al. 2020; Menary et al. 2020; Hassan et al. 2021; Robson et al. 2022). Menary et al. (2020) showed that the multimodel mean AMOC increased significantly over 1850–1985 in historical simulations of CMIP6 models. Furthermore, Menary et al. (2020) attributed the AMOC increase to stronger AAer forcing in CMIP6 compared to CMIP5, primarily due to the inclusion of aerosol–cloud interactions in more models although increases in the temporal variability of CMIP6 emissions may also play a role (Needham et al. 2024). Hassan et al. (2021) showed that CMIP6 AAer simulations yield robust AMOC strengthening (weakening) in response to increasing (decreasing) anthropogenic aerosols during 1900–2020. They argued that AMOC multi-decadal variability is initiated by North Atlantic aerosol optical thickness perturbations to net surface shortwave radiation, sea surface temperature, and hence sea surface density. Robson et al. (2022) analyzed CMIP6 historical simulations in order to understand the processes leading to the anthropogenic aerosol AAer forced increase in AMOC over the period 1850–1985. They split models between “strong” or “weak” sensitivity to AAer forcing and explained differences of AMOC response. They showed that in both strong and weak changes in AAer effects on AMOC are via changes in downwelling surface shortwave radiation over the subpolar North Atlantic (SPNA), similar to Hassan et al. (2021). However, in models with a strong sensitivity turbulent heat loss over the SPNA is significantly larger because the air advected over the ocean is colder and drier, in turn because of greater AAer-forced cooling over the continents upwind, especially North America. Robson et al. (2022) also argued that the strengthening of the AMOC also feeds back on itself positively in two distinct ways: by raising the sea surface temperature and hence further increasing turbulent heat loss in the SPNA, and by increasing the sea surface density across it due to increased northward transport of saline water.

After 1985, the role of AAer forcings on AMOC is less clear. This lack of clarity is partly due to the fact that greenhouse gases are also contributing to a simulated decline. However, the changes in AAer emissions become much more complex, with decreases over North America and Europe, and increases over Asia (Lamarque et al. 2010; Kang et al. 2021) but small declines in global mean emissions that are associated with a global “brightening” (e.g., Wild 2016; Wang et al. 2022). Furthermore, there is some evidence that the response of AMOC to AAer emissions from different regions may be non-linear (e.g., Liu et al. 2024).

#### Natural forcings

The oceanic response to solar forcing associated with the 11-year solar cycle could be amplified by ocean–atmosphere coupling the North Atlantic Ocean (Gray et al. 2013; Scaife et al. 2013; Andrews et al. 2015; Ye et al 2023a). Andrews et al. (2015) and Gray et al. (2016) showed that the accumulated solar energy in the mixed layer of the North Atlantic could generate a response lag of 3–4 years of surface atmospheric pressure to the decadal solar cycle, which in turn could affect AMOC. For example, Ye et al. (2023a) assessed the influence of varied total solar irradiance (TSI) due to the effects of solar activity on AMOC based on an Earth System model with intermediate complexity and the results showed a significant and stable negative correlation between TSI and AMOC on a multidecadal timescale. However, there is a growing debate regarding the influence of solar activity on AMOC change (Ye et al. 2023a).

Volcanic eruptions may also affect the AMOC (Swingedouw et al. 2017; Marshall et al. 2022; Paik et al. 2023) and AMV. Based on a high-resolution 600-year proxy temperature record from the subtropical Atlantic, Waite et al. (2020) detected multidecadal temperature variability from the record which suggests a link between the volcanic eruption and the AMV. Using climate model simulations, Pausata et al. (2015b) found that large summer high-latitude eruptions in the Northern Hemisphere cause strong hemispheric cooling which induces an El Niño-like anomaly during the first 8–9 months after the start of the eruption in response to hemispherically asymmetric cooling. The high-latitude eruption also leads to a strengthening of the AMOC in the first 25 years after the eruption, followed by a weakening of the AMOC that lasts at least 35 years. However, the AMOC response to volcanic forcing is poorly constrained and likely to be sensitive to the period, distribution, and strength of the forcing (Mignot et al. 2011; Bilbao et al. 2024) and the background state (Zanchettin et al. 2013); For example, some models suggesting the response to volcanic forcing is a weakening (Zhong et al. 2011), some a strengthening (Stenchikov et al. 2009; Iwi et al. 2012).

#### Arctic influences on the North Atlantic

Arctic–Subarctic heat and freshwater fluxes play a central role in linking Arctic Ocean variability with the North Atlantic. On the one hand, the North Atlantic constitutes a net source of heat for the Arctic. On the other hand, the Arctic constitutes a source of freshwater for the North Atlantic. In this section, we focus on changes and driving mechanisms of freshwater fluxes from the Arctic into the North Atlantic.

Freshwater enters the Arctic Ocean as net precipitation, as river runoff from the Siberian and Alaskan-Canadian shelves, and as inflow from the Pacific through Bering Strait, and it leaves the Arctic through Davis Strait and eastern Fram Strait, both in the form of liquid freshwater and as ice (Carmack et al. 2016). For instance, over the period 1980–2000, freshwater import and export rates were approximately balanced, with estimates ranging from 7950 ± 400 km^3^ yr^−1^ (Serreze et al. 2006) to 8800 ± 530 km^3^ yr^−1^ (Haine et al. 2015) for the net import rates and from 8720 ± 700 km^3^ yr^−1^ (Serreze et al. 2006) to 8700 ± 700 km^3^ yr^−1^ (Haine et al. 2015) for the net export rates (Carmack et al. 2016).

The release of freshwater from the Arctic into the North Atlantic is not uniform but occurs in isolated time-limited events (Proshutinsky et al. 2015). Between 1950 and 2000, observations indicate four distinct, large Arctic freshwater releases into the North Atlantic. These observed, past Arctic freshwater releases are manifest as distinct periods of cold and fresh polar water in hydrographic observations from the Nordic Seas and subpolar North Atlantic (Belkin et al. 1998; Haak 2003; Belkin 2004; Sundby and Drinkwater 2007). A particularly strong freshwater event was the Great Salinity Anomaly from 1969 to 1972, which was associated with a temporary shutdown of ocean convection in the Labrador Sea, an important ocean convection region (Dickson et al. 1988; Lazier 1980). Weaker freshwater anomalies occurred in the 1980s and 1990s (Belkin et al. 1998; Belkin 2004; Sundby and Drinkwater 2007). Yet, the duration of the Arctic freshwater releases, and the exact pathways, propagation speed and arrival times in the subpolar North Atlantic and Nordic Seas differed between these freshwater releases.

Over the period 2000–2010, the Arctic has accumulated freshwater (Haine et al. 2015; Proshutinsky et al. 2019; Solomon et al. 2021; Wang et al. 2020; 2023; Wang 2021; Timmermans and Toole 2023). Most of the freshwater has been stored in the upper layers of the Arctic Ocean, particularly in the Beaufort gyre, where it is estimated that an extra 5000 km^3^(25%) of freshwater has been stored in the period 2000–2010 compared to the period 1980–2000 (Haine et al. 2015). After the period 2000–2010, the Arctic freshwater storage has stabilised (Solomon et al. 2021). Moreover, a comprehensive set of observations suggests that the cold halocline layer, which caps the warm, salty Atlantic water, has significantly thinned and that further thinning may allow for an emerging freshwater release into the North Atlantic (Lin et al. 2023). The recent observed changes in the North Atlantic salinity are thought to be influenced by the freshwater excess coming from the Arctic (Holliday et al. 2020). However, so far there is only limited evidence of the Arctic freshwater fluxes impacting freshwater accumulation in the Labrador Sea and the North Atlantic (Florindo-Lopez et al. 2020).

The extended period of accumulation and increased storage of freshwater in the Arctic over the last two decades has primarily been attributed to the wind forcing (Giles et al. 2012; Haine et al. 2015; Proshutinsky and Johnson 1997). Specifically, a more cyclonic ocean and atmospheric circulation in the Arctic have been suggested to lead to enhanced outflow of freshwater from the Beaufort Gyre into the Transpolar drift, and in the Arctic boundary currents following the shelf slopes, then further through Davis Strait and Fram Strait into the Nordic Seas and North Atlantic (Proshutinsky and Johnson 1997; Proshutinsky et al. 2019; Solomon et al. 2021). On the other hand, a more anticyclonic circulation promotes an enhanced storage of freshwater in the Beaufort Gyre, due to wind-driven Ekman transports setting up a cross-gyre pressure gradient with increased sea level in the central gyre (Proshutinsky and Johnson 1997). This accumulation can be balanced by the eddy transports releasing freshwater from the gyre (Armitage et al. 2020). This fresh water leakage from the gyre due to eddies may become larger as sea ice declines and the ocean spins up (Meneghello et al. 2018).

Over the period 2000–2015, the atmospheric and oceanic circulation in the Arctic have primarily been in a more anticyclonic regime (Armitage et al. 2017; Proshutinsky et al. 2015, 2019; Kelly et al. 2019; Regan et al. 2019). The observed changes in the Arctic surface currents for the more recent years were indicative of the Arctic ocean circulation returning to the more cyclonic state with the Beaufort Gyre shrinking back (Lin et al. 2023; Nishino et al. 2023).

While past Arctic freshwater releases constituted an integral part of the low-frequency, decadal variability (Zhang and Vallis 2006), the extent to which Arctic ice and freshwater releases act as a driver, a response or a side effect of the North Atlantic low-frequency variability is unclear. Progress is impeded by reduced spatial and temporal coverage of long-term salinity observations, as well as by freshwater biases in models (Mecking et al. 2017; Menary et al. 2015). In theory, Arctic releases of cold and fresh polar water into the subpolar region could lead to an increased meridional SST gradient and thus, an increase in atmospheric instability, triggering atmospheric feedbacks (Oltmanns et al. 2020, 2024) which give rise to predictability (Zhang and Vallis 2006). For instance, subpolar cold and freshwater anomalies are typically coupled to a positive North Atlantic Oscillation, which is associated with a stronger wind stress curl increasing the advection of cold and fresh polar water into the subpolar gyre (Häkkinen and Rhines 2004; Häkkinen et al. 2013; Holliday et al. 2020; Oltmanns et al. 2020).

In turn, changes in the North Atlantic Ocean and atmospheric circulation can feed back on processes in the Arctic by modulating ice and freshwater outflows. Specifically, observations show an increased heat transport into the Arctic due to warmer Atlantic water, resulting in a thinning of the halocline (Asbjørnsen et al. 2020; Polyakov et al. 2017, 2023; Tesi et al. 2021; Wang 2021). By integrating a two-sided coupling between the Arctic and North Atlantic into an idealised delayed, harmonic oscillator model, it is possible to reproduce the observed, multi-decadal variability of the North Atlantic Ocean (Wei and Zhang 2022). Still, the active role of the North Atlantic Ocean and atmospheric circulations in influencing the ocean and atmospheric circulations in the Arctic and hence, ice and freshwater exports are largely unknown.

### Attribution of observed changes

Observed AMOC changes estimated from various trans-basin monitoring arrays (Fig. 7a, see also Volkov et al. 2021; Jackson et al. 2022) showed a rising trend from 2000 to about 2006, a subsequent declining trend until about 2010, and a recovery afterwards. CMIP5 and CMIP6 models produce a forced weakening of the AMOC over the 2012–2017 period relative to 2004–2008, but at 26° N the multi-model mean response is substantially weaker than the observed AMOC decline over the same period. The discrepancy between the modelled multi-model mean and the RAPID observed AMOC changes has led studies to suggest that the observed weakening over 2004–2017 is largely due to internal variability (Yan et al. 2018). In summary, models do not support robust assessment of the role of anthropogenic forcing in the observed AMOC weakening between the mid-2000s and the mid-2010s and there is low confidence that anthropogenic forcing has influenced the observed changes in AMOC strength in the post-2004 period (e.g., Eyring et al. 2021).

Previous studies shown that increasing concentrations of GHG act to weaken the AMOC in climate models (Gregory et al. 2005; Delworth and Dixon 2006; Stouffer et al. 2006; Caesar et al. 2018; Thornalley et al. 2018; Menary et al. 2020; Eyring et al. 2021) and increasing AAer forcings tend to strengthen the AMOC (Cai et al. 2006; Delworth and Dixon 2006; Menary et al. 2013; Undorf et al. 2018a, b; Andrews et al. 2020; Menary et al. 2020; Hassan et al. 2021; Robson et al. 2022). These competing anthropogenic effects were thought to lead to relatively little externally forced change of AMOC over the historical period in some early studies (Delworth and Dixon 2006; Cheng et al. 2013). More recent studies showed that an increase of AMOC over the historical period in CMIP6 models due to AAer forced increases overwhelm GHG induced decreases, resulted from stronger AAer forcing in CMIP6 compared to CMIP5, primarily due to the inclusion of aerosol–cloud interactions in more models (Menary et al. 2020; Hassan et al. 2021; Robson et al 2022). However, the increase in the historically simulated AMOC in CMIP6 is in stark contrast with weakened AMOC, estimated since at least 1950 from observed surface temperatures or sea surface height (Caesar et al. 2018, 2021) or from reconstructions based on sediment-based proxies (Thornalley et al. 2018). In addition, models simulate a range of anthropogenic aerosol effective radiative forcing and a range of historical AMOC trends in CMIP6 (Menary et al. 2020) and there remains considerable uncertainty over the realism of the CMIP6 AMOC response during the twentieth century. As a result, there is low confidence that anthropogenic forcing has had a significant influence on changes in AMOC strength during the 1860–2014 period (e.g., Eyring et al. 2021).

### Projected changes

**Fig. 8:**
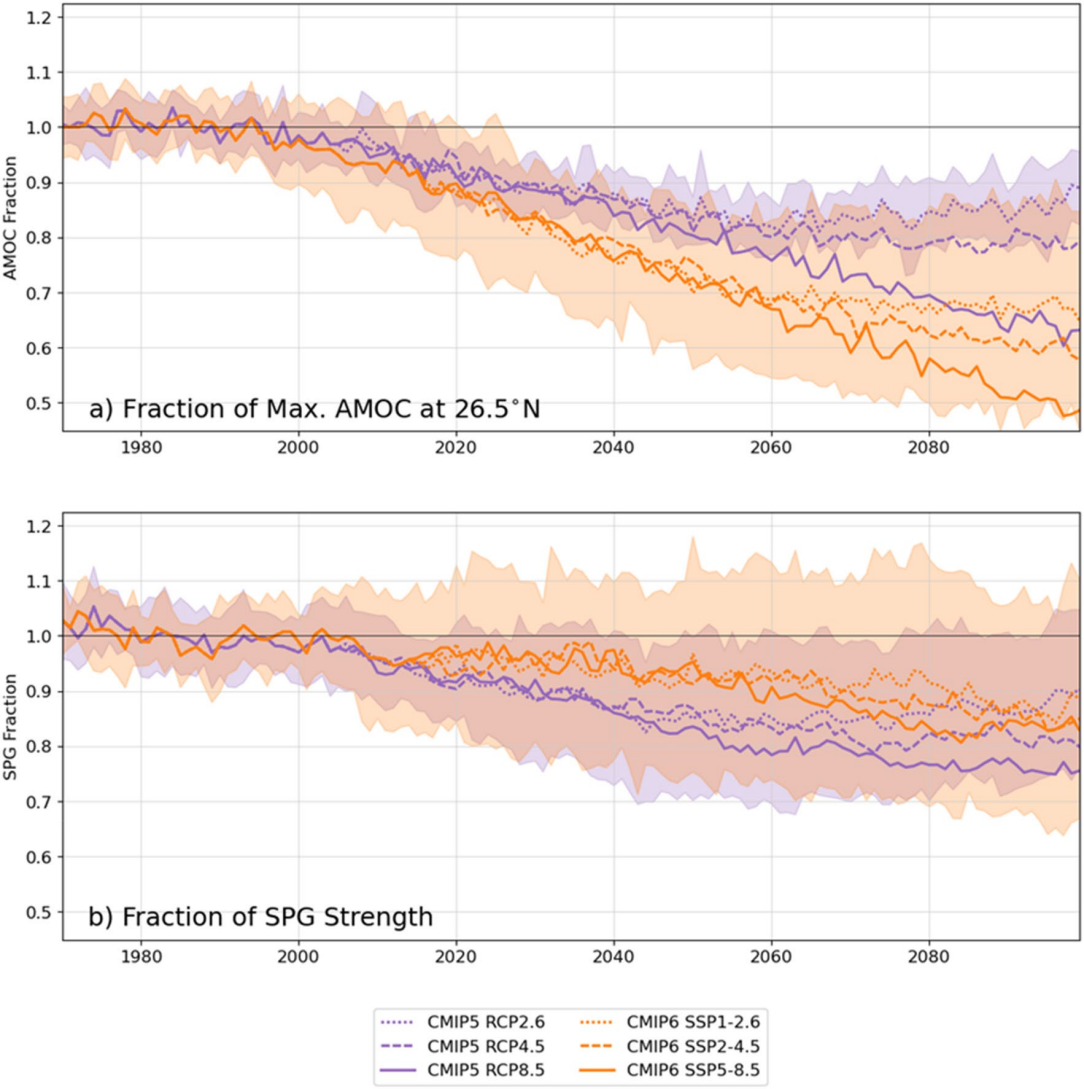
The fraction of the maximum AMOC at 26.5° N (**a**) and SPG strength (**b**) (defined as minimum streamfunction in 60–15° W, 48–65° N) with respect to the 1970–1999 historical reference period for CMIP5 (purple) and CMIP6 (orange) and future climate projections RCP2.6/SSP1-2.6 (dotted line), RCP4.5/SSP2-4.5 (dashed line) and RCP8.5/SSP5-8.5 (solid line). The shading shows ± one standard deviation of the RCP2.6/SSP1-2.6 scenarios. For following models were used: CMIP5 - CCSM4, CESM1-CAM5, CNRM-CM5, CSIRO-Mk3-6-0, GFDL-ESM2G, MPI-ESM-LR, MPI-ESM-MR, NorESM1-ME, NorESM1-M and CMIP6 - CESM2-WACCM, CIESM, CMCC-ESM2, CanESM5, EC-Earth3-Veg-LR, EC-Earth3, FGOALS-g3, HadGEM3-GC31-LL, MIROC6, MPI-ESM1-2-HR, MPI-ESM1-2-LR, MRI-ESM2-0, NorESM2-LM, UKESM1-0-LL (Adopted and expanded from Mecking and Drijfhout 2023)

Future climate projections in the CMIP phases 3, 5 and 6 have consistently shown decreases in the AMOC until the end of the twenty-first century (Fig. 8a; Meehl et al. 2007; Fox-Kemper et al. 2021). According to the IPCC AR6 the multi-model mean AMOC is projected to decline by 24% with a 95% confidence interval of 4–46% and 39% with a 95% confidence interval of 17–55% for the low emission scenario, SSP1-2.6, and the high emission scenario, SSP5-8.5, respectively (Fox-Kemper et al. 2021). The reduction in the AMOC in CMIP6 is larger than in previous CMIP phases (Weijer et al. 2020), and the known minor differences in forcing (Lee et al. 2021, Fig. 4.35), are not enough to explain the differences in AMOC response (Mecking and Drijfhout 2023). Several studies have shown a stronger role for ocean warming relative to freshening in AMOC reduction (Gregory et al. 2005; Levang and Schmitt 2020; Couldrey et al. 2021), even when the AMOC decline is forced using freshwater hosing as opposed to global warming (Haskins et al. 2020).

A change in the AMOC is associated with changes in density in the high latitude North Atlantic, in particular, the Labrador and Irminger Seas (Ba et al. 2014; Heuzé 2017). Furthermore, recent studies linked sea ice changes (Sévellec et al. 2017; Sun et al. 2018; Liu et al. 2019; Dai 2022), diffusive upwelling in the Indo-Pacific (Baker et al. 2023), aerosols and air quality (Hassan et al. 2022) to AMOC reduction. The recent study, Asbjørnsen and Årthun (2023), investigated changes in the Gulf Stream and deep western boundary current in the CMIP6 SSP5-8.5 scenario, showing a weakening of 47% for the deep western boundary current and 29% reduction in Gulf Stream in 2090–2100 relative to 2015–2025. Using a different method of defining the Gulf Stream, Sen Gupta et al. (2021) find a reduction of 15% in CMIP5 and CMIP6 models in 2050–2100 relative to 1900–2000. 33% of the weakening of the Gulf Stream can be explained by changes in the wind (Asbjørnsen and Årthun 2023). Multi-model means across future climate projections in CMIP3/5/6 show a reduction in the strength of the subpolar gyre (Reintges et al. 2017; Fox-Kemper et al. 2021) as well as a poleward shift of the subtropical gyre (Yang et al. 2020). However, not all models show a reduction in the strength of the subpolar gyre, for example MPI-ESM1.1 initially has an increase in gyre strength in model simulations with CO_2_ emissions from pre-industrial levels increased by 1% per year due to density differences between the eastern and central subpolar gyre, which after 2 K warming no longer changes strength and is pushed northward due a northward shift of the subtropical gyre (Ghosh et al. 2023).

#### Tipping points

**Fig. 9:**
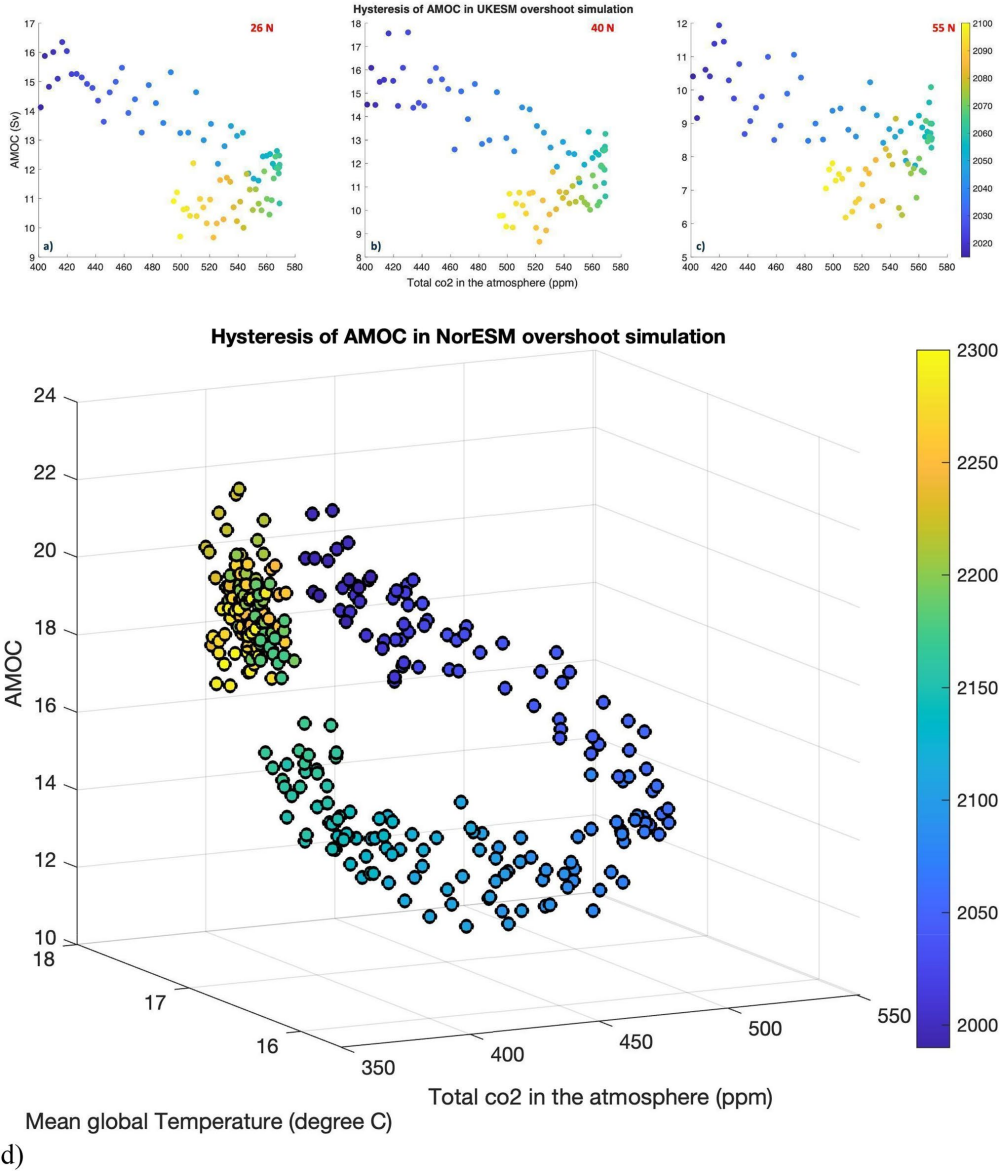
Delayed response (hysteresis) of the annual Atlantic Meridional Overturning Circulation (AMOC) strength (Sv) up to year 2100 in the CDR-MIP esm-SSP534 concentration-driven overshoot scenario integrations with the UKESM model at 26° N –corresponding to the “RAPID” observational array (**a**), at 40° N (**b**), and at 55° N (**c**). Only one ensemble member is shown; the colour bars indicate the time sequence. Note the different vertical axes (Adapted from Heinze et al. 2023). **d** AMOC hysteresis loop in the esm-SSP534-ov emission-driven NorESM long integrations until 2300 (original analysis, simulations are described by Tjiputra et al. (2023)

There are two commonly considered tipping points in North Atlantic circulation, the AMOC collapse on centennial time scales and a substantial cooling in the SPG (Fig. 9, Swingdouw et al. 2020; Loriani et al. 2023a, b). While CMIP models have shown that they generally do not simulate an AMOC to collapse before the end of the twenty-first century (Fig. 8), recent studies warn that a collapse could happen sooner (Boers 2021; Ditlevsen and Ditlevsen 2023). Due to the short time period of available RAPID mooring array (2004-present) it is difficult to come to conclusions of the current trajectory of the AMOC behaviour, with Lobelle et al. (2020) suggesting that at least 29–67 year of data is required from the RAPID array to detect a decline in AMOC for the 90% confidence level of detection. Both statistical (Boulton et al. 2014) and physical (van Western et al. 2024) methods have been suggested as early warning signals for AMOC collapse, but they require several decades of data to robustly detect an abrupt change. Rapid cooling event in the SPG due to a collapse of deep convection on timescales of around a decade have been seen in the future projections of both CMIP5 (Sgubin et al. 2017) and CMIP6 (Swingedouw et al. 2021). The likelihood of this abrupt cooling event in the SPG has been estimated at 45.5% from CMIP5 and 36.4% from CMIP6.

#### AMOC recovery

The IPCC AR6 report asserts with “high confidence” that the projected by the CMIP6 models future AMOC decline can be reversible on the multi-centennial timescales if the anthropogenic-induced radiative forcing is reversed; this can be done, for example, following carbon dioxide removal scenarios (Sect. 4.6.2 and Table 4.10 in Lee et al. 2021). However, the AMOC still does not recover until the end of the century either in the idealised simulation of the Carbon Dioxide Removal intercomparison (CDR) project (Keller et al. 2018) or in the emission-driven overshoot scenarios esm-SSP534 (Schwinger et al. 2022; Heinze et al. 2023; Loriani et al. 2023a, b). In addition, there is emerging evidence that recovery of the AMOC and of the surface atmospheric temperature (SAT) can vary for different latitudes (Fig. 9), also noted by Heinze et al. (2023) and Schwinger et al. (2022). Specifically, a transient, ~ 100-year long, strong cooling of the low atmosphere (and SAT) in the high latitudes can occur when a cooling climate due to carbon dioxide removal coincides with a still-weak AMOC (Schwinger et al. 2022). A transient cooling hiatus has been also reported in the simulation with the ramp down of CO_2_ emissions to zero (but without negative emissions) (An et al. 2021). Both effects are indicative of a potential partial decoupling of poleward heat transport from AMOC decline (Smedsrud et al. 2022).

Hysteresis and bistability both refer to systems which can adopt one of two or more states for the same external forcing, such as CO_2_ concentration, with the subtle distinction that bistability implies the potential for abrupt or rapid transitions between the states, whereas hysteresis does not carry this implication (e.g., Boucher et al. 2013). Commonly, this is explored by approaching the same external conditions with different trajectories in model simulations, e.g. increasing and reversing the forcing to study reversibility. Bistability involving a full collapse of the AMOC by artificially flooding the North Atlantic with freshwater has been demonstrated (or strongly implied) in theoretical models (Stommel 1961) and climate models of reduced complexity (Rahmstorf et al. 2005) or of low resolution (Hawkins et al. 2011; Van Westen et al. 2024). The freshwater anomalies required to shut down the AMOC in Hawkins et al. (2011) and in van Westen et al. (2024) far exceeds the freshwater equivalent contained in the Greenland Ice sheet. We don't know whether an AMOC shutdown could happen with the much smaller FW anomalies that could occur in the real world.

In more complex or higher resolution models it is difficult to conduct experiments for long enough to demonstrate bistability or hysteresis. However, weak states have been shown to be stable for at least 100 years in about half of a test group of CMIP6-type models (Jackson et al. 2023a) and in high-resolution ocean–atmosphere coupled climate models (Mecking et al. 2016). A recent study finds AMOC tipping in a CMIP6-type model in response to gradually increasing freshwater release in the North Atlantic thought to be consistent with a realistic Greenland Ice Sheet mass-loss scenario (Van Westen et al. 2024). AMOC bistability is model-dependent, controlled by the balance of the positive and negative feedbacks that determine the salinity of the subpolar North Atlantic. It is not yet understood why collapse occurs in some models and not others (Jackson et al. 2023b). However, there is evidence that the present generation of climate models is too stable due to model biases in the distribution of ocean salinity (Liu et al. 2017; Mecking et al. 2017).

Bistability is harder to prove in transient climate change simulations because, by definition, the external forcing is changing, so the system is not in equilibrium and may also respond with a time lag to the forcing. Nevertheless, overshoot scenarios, where the CO_2_ trend is assumed to reverse at some point in the future, provide some useful information about reversibility of the AMOC on human timescales. UKESM runs under an overshoot emission scenario exceeding and returning to 500 ppm show that even if CO_2_ concentrations return to 500 ppm by 2100 the AMOC is still only 77 percent of the strength it was in 2050 when CO_2_ concentration was also at 500 ppm (Fig. 9). Although the AMOC does not collapse in this model, Fig. 9d shows that full recovery takes on the order of 300 years in NorESM.

#### Caveats to projected changes

Several studies looking into future projections have focused on the most extreme future projection scenario (i.e. Asbjørnsen and Årthun 2023), but the most extreme scenario is often seen as misleading so caution has to be used when looking at results (Hausfather and Peters 2020). Other uncertainty arises because concentration-driven (with prescribed atmospheric CO_2_ concentration) projections in the CMIP5/6 experiments impose extra control on the radiative forcing, as opposed to emission-driven runs. This makes high-end climate projections significantly warmer in the emission-driven simulations due to stronger carbon cycle feedbacks (Friedlingstein et al. 2014).

On the other hand, models are designed to be stable in the present-day climate which means it is difficult to know whether they are capable of capturing extrema and abrupt changes (Valdes 2011). Coupled climate models and earth system models have biases in the mean state which can have an impact on the North Atlantic variability (Menary et al. 2015; Reintges et al. 2024) and AMOC reduction (Mecking et al. 2017). A commonly known bias is the fresh bias in the upper 500 m of the South Atlantic, which when corrected through flux adjustment leads to a larger decline in AMOC strength (Liu et al. 2017). Furthermore, the impact of melting of the Greenland Ice Sheet, a source of freshwater input into the North Atlantic which is not included in CMIP5 and CMIP6 models, varies between very little additional AMOC decline to almost double AMOC decline, dependent on the model used in AMOC emulators (Bakker et al. 2016). Finally, the majority of future climate projections in CMIP6 are using coupled climate models with an ocean resolution of ~ 1 degree, which does not permit or resolve eddies. The higher resolution models allow for stronger more realistic boundary currents, resolving and/or permitting eddies (Hirschi et al. 2020). Higher resolution can have an impact on the model biases (Jüling et al. 2021) and salinity budget facilitating AMOC collapse (Mecking et al. 2016).

For the changes in the Arctic, CMIP6 model simulations mostly disagree with observations in the Arctic mean ocean state and variability (Zanowski et al. 2021). Moreover, in future projections covering the twenty-first century, CMIP6 model simulations also exhibit substantial differences in the Arctic mean states and the magnitude of the freshwater storage and flux changes (Zanowski et al. 2021). Yet, most models agree that the warming of Atlantic waters flowing into the Arctic will continue to lead to an increased poleward ocean heat convergence in the twenty-first century (Wang et al. 2023). The models also predict an increase in freshwater input in the Arctic and in freshwater export rates into the North Atlantic, particularly through Fram Strait, due to both higher volume transports and a reduced salinity (Wang et al. 2023). Therefore, whereas it might be challenging to have confidence in the overall projected Arctic changes, trends in the Arctic-North Atlantic linkages can be assessed with high confidence.

## Ocean–atmosphere interactions

Ocean–atmosphere interactions play a major role in shaping changes in atmosphere and ocean circulation in the North Atlantic region. Mid-latitude atmospheric variability over the North Atlantic is known to strongly influence the underlying ocean. Anomalies in air-sea heat flux drive ocean convection and changes in local heat content over the subpolar gyre (Visbeck et al. 2003; Grist et al. 2010; Josey et al. 2018). Changes in wind stress drive variations in heat content and Ekman circulations (Lozier et al. 2008; Williams et al. 2014). The combined effect of wind stress and air sea buoyancy flux changes drive variations in the AMOC (Lozier et al. 2010; Robson et al. 2012), which in turn alters SST over the Atlantic through changes in heat transport convergence (Delworth et al. 1993; Kushnir 1994; Williams et al. 2014; Zhang et al. 2019; Srokosz et al. 2021; Jackson et al. 2023a; Robson et al. 2023).

Changes in the ocean circulation and state influence atmospheric circulation (Magnusdottir et al. 2004; Brayshaw et al. 2011; Yeager and Robson 2017; Sutton et al. 2017; Simpson et al. 2018; Ma et al. 2020; Chemke et al. 2022; Strommen et al. 2023). In particular ocean dynamics have been shown to play a significant role in controlling air-sea heat flux variability on interannual to multidecadal timescales (Josey and Sinha 2022; Moat et al. 2024). Variations in the AMOC and North Atlantic gyres have been argued to modify the seasonal to decadal variability of the North Atlantic jet stream and storm track, in part via changes in the AMV (Gastineau et al. 2013; Gulev et al. 2013; Frankignoul et al. 2015; Ciasto et al. 2016; Gervais et al. 2019; Qasmi et al. 2020; Bellomo et al. 2021; Ruggieri et al. 2021). There is also evidence that the AMOC is an important factor in modulating the response of the North Atlantic jet stream and storm track in winter to greenhouse gas forcing in CMIP3, CMIP5 and CMIP6 simulations (Woollings and Blackburn 2012; Bellomo et al. 2021). Modelling studies have shown further that the development of the North Atlantic “warming hole” (a cold temperature anomaly in subpolar North Atlantic, e.g. Drijfhout et al. 2012) in response to anthropogenic forcing changes could impact the intensity and location of the mid-latitude jet stream and storm tracks (Woollings and Blackburn 2012; Gervais et al. 2019; Karnauskas et al. 2021); these changes also influence the ocean state, suggesting a two-way coupling between the atmosphere and ocean.

Different mechanisms have been put forward to understand how the AMV affects North Atlantic atmospheric circulations in winter, including direct modulation of low-level baroclinicity and stationary waves by the North Atlantic SST anomalies (Kushnir 1994; Kushnir et al. 2002; Msadek et al. 2011; Peings et al. 2016; Zhang et al. 2019), forcing from the tropical Atlantic (Davini et al. 2015), and stratospheric pathways (Omrani et al. 2014). The relative importance of these different mechanisms, and how they may interact, is not known. In summer, there is evidence of an atmospheric circulation response to positive phase of AMV which projects on the negative summer NAO (Sutton and Hodson 2005; Sutton and Dong 2012; Qasmi et al. 2021); this may reflect a response to weakening of the meridional SST gradient between the subtropical and subpolar gyres.

## Multi-annual predictability and prediction

### Dynamical predictability and prediction

It has long been thought that there should be significant potential to predict ocean circulation changes in the North Atlantic on multi-year to decadal timescales. For example, idealised prediction experiments, whereby a model attempts to predict its own evolution, indicated that AMOC could be predicted for up to a decade in advance, and sometimes longer (Griffies and Bryan 1997; Collins and Sinha 2003; Collins et al. 2006). Such predictability was thought to also lead to the potential to predict SSTs and other features of the North Atlantic (Duchez et al. 2016a). There is also evidence that atmospheric circulation responds to SST changes (Kushnir 1994; Msadek et al. 2011; Kushnir et al. 2002; Peings et al. 2016) or changes in external forcings (Ortega et al. 2015; Sjolte et al. 2018), suggesting that prediction of decadal changes in atmospheric circulation could also be possible. However, there was little evidence in the early studies for significant potential predictability in the Atmospheric circulation, particularly in winter (Griffies and Bryan 1997; Collins et al. 2006).

The importance of predicting changes in ocean circulation has been underlined by the assessment of near-term climate predictions. For example, it has been argued that the initialisation of ocean circulation, and primarily the buoyancy forced ocean circulation (including both AMOC and gyre), have been key to delivering improved predictions of North Atlantic SST, especially on decadal timescales. For example, many studies have highlighted that successful predictions of SPNA was dependent on the initialisation of ocean circulation and ocean heat transport (e.g., Robson et al. 2012, 2014, 2018; Yeager et al. 2012, 2015; Yeager and Robson 2017; Msadek et al. 2014; Borchert et al. 2021), and decadal time-scale cooling of the SPNA in the 1960s and after 2005. Such predictability was associated with the initialisation of subsurface density anomalies, which then propagate southward and interact with the topography (Robson et al. 2014; Yeager and Robson 2017; Yeager 2020).

Although the initialisation of the ocean circulation (e.g., the strength of the circulation at the start of the prediction) has been shown to be important to deliver skillful predictions of the North Atlantic Ocean, there is less evidence that the changes in ocean circulation are predictable themselves. Indeed, when assessed in depth space, the skill of AMOC was shown to be limited to lead times of a few years ahead, and significantly less predictable than related variables, including sub-surface density anomalies, sea surface height and subpolar heat content (Menary et al. 2016; Yeager and Robson 2017; Yeager 2020). Predictability of subsurface density anomalies in regions such as the Labrador Sea was also low (Yeager and Robson 2017). This lack of skill in the AMOC could be associated with the lack of consistency in initial conditions (e.g., Karspeck et al. 2017), complicated and non-linear drifts in mean-states and variability (Menary and Hermanson et al. 2018), or due to the lack of skill in predicting atmospheric circulation changes (Yeager and Robson 2017). However, recent work has also highlighted that AMOC in density space may be significantly more predictable than AMOC in depth space (Yeager 2020).

Although it was previously thought that the atmospheric circulation was not predictable on multi-year or longer timescales, there is now significant evidence that North Atlantic Atmospheric circulation can be predicted on a range of time-scales. For example, there is evidence that the winter NAO can be predicted over a year ahead (Dunstone et al. 2016), and low-frequency changes in the winter NAO are also predictable (Smith et al. 2020). Furthermore, skill has also been reported in both wintertime atmospheric blocking (Athanasiadis et al. 2020), and also in the jet latitude (Marcheggiani et al. 2023) and jet speed (Marcheggiani et al. 2023; Strommen et al. 2023).

However, the predictable signals in the atmospheric circulation are substantially weaker than would be expected. In particular, the signal-to-noise ratios are small yet the forecast skill is relatively large, leading to the counterintuitive situation where the predictability of the real world exceeds the predictability within the model world. For example, for predictions of winter NAO on both 2 year timescales and 2–9 year timescales, the predicted signals are > 2 (Dunstone et al. 2016) and > 10 (Smith et al. 2020) times smaller than expected based on the ratio of predictable component between real and model world (Eade et al. 2014; Weisheimer et al. 2024). This so-called signal-to-noise paradox (Scaife and Smith 2018) very much remains an open question, despite numerous studies exploring different facets of the problem and a conclusive solution to the problem has not yet been reached (Weisheimer et al. 2024).

In summer, the predictability of the circumglobal teleconnection originating in the North Atlantic region can provide skillful predictions of surface temperature over Europe and East Asia, including seasonal extremes (Monerie et al. 2018; Borchert et al. 2019), but there is less evidence of successful predictions of atmospheric circulation such as the summer NAO on multi-annual-to-decadal timescales.

Unfortunately, there is less understanding of the processes that are leading to skill. In particular, the sources of skill for the winter NAO predictions for years 2–9 is currently unknown, which could limit confidence in future predictions. There are some indications, for example, that changes in external forcing are able to provide some skill (e.g., Klavans et al. 2021). However, the skill for NAO is sensitive to the time-period over which it is computed, and decadal predictions currently do not capture the return to positive winter NAO following 2010 (Marcheggiani et al. 2023). Changes in the skill in the NAO could be related to changes in the role of external forcings (e.g., different forcing factors), non-stationarity in NAO predictability (e.g., Weisheimer et al. 2017; 2020) and ENSO teleconnections (e.g., O’Reilly et al. 2019), or degraded predictions of North Atlantic SST post 2010 (e.g., Marcheggiani et al. 2023).

### Machine learning

Recently there has been a growing interest in utilising machine learning (ML) techniques as a new approach to weather and climate forecasting. Various studies show that neural network-based models trained on either reanalysis data or model data can produce reliable predictions, which in turn can be used to identify forecasts of opportunity (Bihlo 2021; Dueben and Bauer 2018; Weyn et al. 2021; Gordon and Barnes 2022). While still in its infancy, these data-driven approaches would enable issuing forecasts and mechanistic understanding of climate-related processes at several orders of magnitude faster than conventional NWP models (Pathak et al. 2022).

Such studies also use a broad hierarchy of ML methodologies including logistic regression (LR), neural networks (NN), convolutional neural networks, generative adversarial networks. ML techniques such as NN offer a valuable means of uncovering potential sources of predictability among large-scale factors yet such methodologies have long been regarded as “black boxes,” making it challenging to decipher the specific relationships learned by the network. Recent advancement of explainable machine learning (XML) methods to climate sciences (Hall et al. 2019; McGovern et al. 2019; Toms et al. 2020; Gordon and Barnes 2022; Sun et al. 2014) now makes it possible to gain insight into the ML's understanding of the complex interrelationships between climate factors.

Although ML techniques have effectively been used to predict climate variability, particularly the El Niño-Southern Oscillation (Ham et al. 2019; Colfescu et al. 2024), there has been a lack of research in using these techniques for predicting long-term, decadal to multidecadal variability, such as the AMV. This is largely due to the limited availability of data. Unlike interannual modes like ENSO, which have ample observational data for training and testing, predicting a single AMV cycle would require 60–70 years of data, making it challenging to train and test a neural network solely on observational data.

**Fig. 10:**
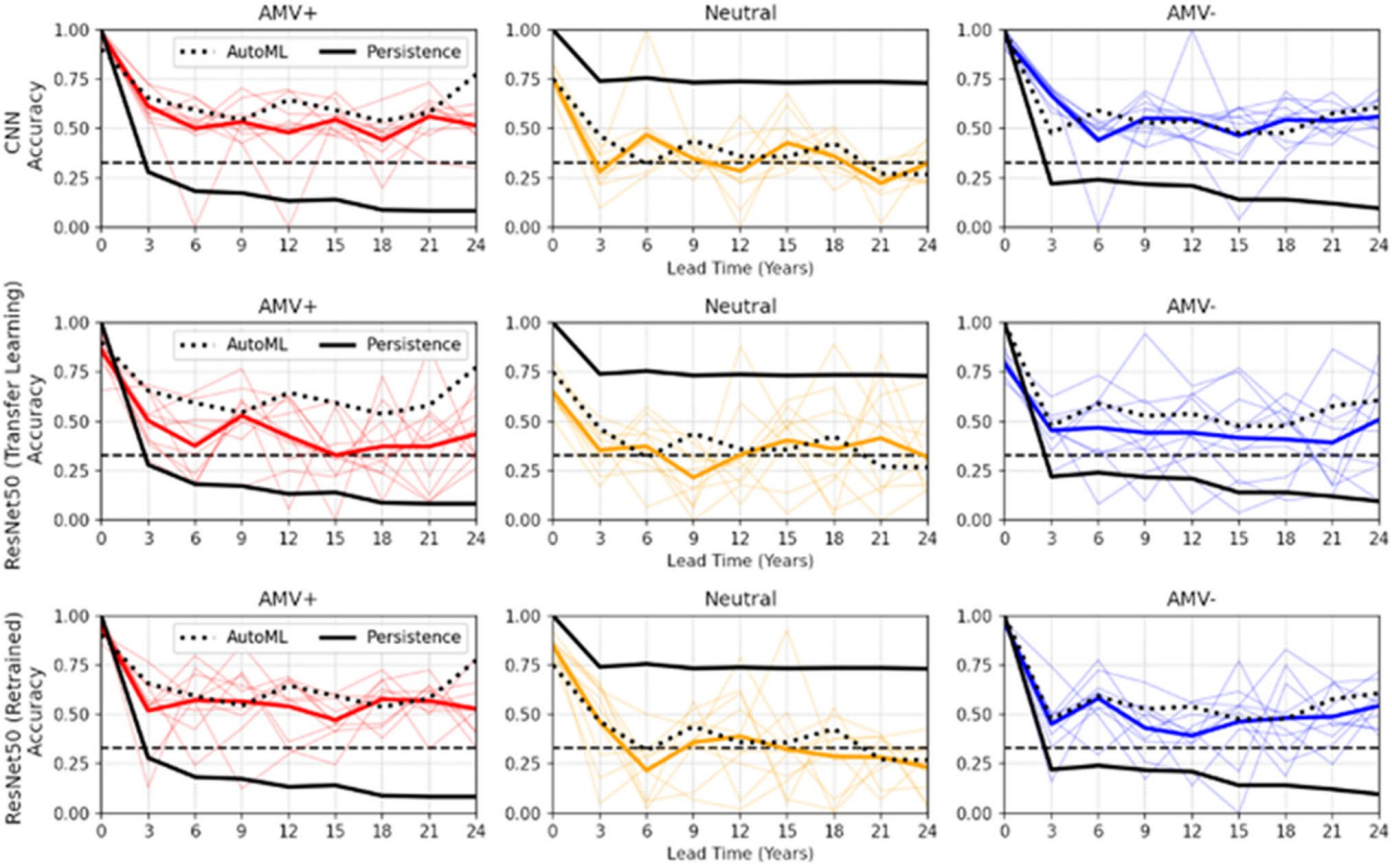
Accuracy of AMV state predictions by lead year for each class (columns) and each machine learning model (rows). AutoML results (dotted black line), persistence baseline (solid black line), and random chance (33% for each class, dashed black line) are shown on each subplot for comparison. For each of the CNN and ResNet50, we performed 10 ensemble runs with different initial conditions, each of the ensemble is shown in the thin colored lines, and the thick colored lines indicate ensemble mean. This image is adapted from Fig. 2 of Liu et al. (2021) https://arxiv.org/pdf/2111.00124

Some previous studies have used climate model data to test ML methodologies for prediction of the north Atlantic sea surface temperature variability. For example, Gordon and Barnes (2022) have used CESM2 LE and artificial neural networks to identify initial climate states where the signals rise above the noise and obtain better predictions for long-term SSTs, in particular, patterns of heat in the Atlantic and Pacific Oceans. Mayer and Barnes (2021) demonstrate that neural networks can identify patterns of storminess ideal for predicting atmospheric circulation over the North Atlantic (up to 4 weeks in advance) while Mercer (2021) uses multiple machine learning methods to quantify the predictability of the cold-season monthly phases of four standard teleconnection indices, the North Atlantic Oscillation (NAO), the Pacific North American oscillation (PNA), the West Pacific Oscillation (WPO), and the Arctic Oscillation (AO). Liu et al. (2021) have used CESM1 LE and a hierarchy of multiple machine learning models to improve the state of AMV prediction (Fig. 10). Their results consistently show that all of the models used outperform the traditional persistence forecast baseline. While not directly trained with observational data, such ensemble model trained networks can be used to then successfully predict long term variability in an observational data set as in Liu et al. (2023). Their study shows the phasing of multidecadal variability in an observational data set using a ML based approach trained on CESM1 and examines AMV predictability with and without external forcings. Similarly, Pasini et al. (2022) uses a data driven ML approach to investigate whether the AMO is forced by natural variability or external forcing and finds that a forced component of AMO in the last 150 years is coming from anthropogenic sulphates.

While such methodologies are still in their infancy, there is a broad range of original application of ML models to future predictions of the decadal to multidecadal variability, including prediction and mechanistic understanding of AMV in observational sets.

## Synthesis and discussions

Understanding large-scale changes in North Atlantic atmospheric and oceanic circulations, their associated climate impacts, and their predictability is crucial for predicting future climate change and variability over the UK and western Europe. In this review, we have synthesized evidence from observations and climate simulations to provide a comprehensive picture of our current understanding of these changes. Firstly, we documented large-scale changes of atmospheric and oceanic circulations over the North Atlantic sector with a focus on decadal–multidecadal changes during the recent decades. Secondly, we discussed the drivers and physical processes which influence these changes. Thirdly, we summarized projected changes in the future. Finally, we discussed predictability and predictions of these circulation features. Some of major points discussed and synthesised are:

- Observations/reanalyses showed notable decadal–multidecadal changes in atmospheric circulation over the North Atlantic sector in all seasons with some distinct features. Prominent decadal variations of atmospheric circulation in winter are characterized by a strengthening and northward shift of the Atlantic jet from the 1960s to the 1990s, weakening and southward shift from the 1990s to 2000s and recent strengthening in jet speed with weak change in jet latitude (Fig. 2). In summer, multidecadal changes are characterized by poleward migration and decreasing jet speed during from 1950 to 1970s, followed by an equatorward migration and increasing jet speed from 1980s (Fig. 3).
- However, climate models have struggled to capture these observed decadal–multidecadal variations of atmospheric circulation over the North Atlantic in both winter and summer (Bracegirdle et al. 2018; Simpson et al. 2018; Blackport and Fyfe 2022; Harvey et al. 2023). These inconsistencies suggest either that the models do not correctly simulate the responses to the external forcings, or that external forcing datasets used to drive models are in error, or both. Alternatively, they could imply that the discrepancy between observed changes and model simulated changes arises simply from chaotic internal variability (Simpson et al. 2018; Peings et al. 2021; Screen et al. 2022). The weak responses to external forcings in the models have been attributed to a number of reasons. These include: weak atmospheric responses to sea-surface temperature (SST) anomalies (Peings et al. 2016; Simpson et al. 2018; Bracegirdle 2022); weak eddy feedbacks (Smith et al. 2017; Scaife et al. 2019; Ruggieri et al. 2021; Hardiman et al. 2022); weak ocean–atmosphere coupling in the North Atlantic (Osso et al. 2020; Zhang et al. 2021); or model biases in North Atlantic atmospheric circulations and SSTs (Scaife et al. 2011; Keeley et al. 2012; Woollings and Blackburn 2012; Smith et al. 2017; Harvey et al. 2020; Simpson et al. 2020; Ruggieri et al. 2021; Bracegirdle et al. 2022; Hermoso et al. 2023).
- Multi-model mean projected changes in the North Atlantic jet show a notable poleward shift in summer and an eastward extension and strengthening in winter. However, a consistent picture or mechanism for these changes under future climate change does not exist since there are large uncertainties in projected changes among different climate models (Simpson et al. 2014; Zappa and Shepherd 2017; Peings et al. 2018; Harvey et al. 2020, 2023; Oudar et al. 2020; Lee et al. 2021; McKenna and Maycock 2021). Opposite effects of different drivers can give rise to more complex patterns than simple shifts in the North Atlantic jet (Oudar et al. 2020; Lee et al. 2021), including an eastward elongation and narrowing of the North Atlantic jet stream (e.g., Peings et al. 2018; Harvey et al. 2020, 2023; McKenna and Maycock 2021), with potential impacts on regional climates (e.g., Harvey et al. 2023).
- Surface forced ocean simulations (e.g., Megann et al. 2021), surface forced overturning circulation measures (Josey et al. 2009), free running ocean models, state estimates and observation based proxies generally suggest that the AMOC showed an increasing trend from 1980 to the mid-1990s, and a decline since then, with the peak occurring a few years earlier in subpolar latitudes compared to subtropical latitudes (Jackson et al. 2022). The relatively strong subpolar gyre in the early 1990s (Chafik et al. 2019) was coincident with a strengthening AMOC, itself brought about by persistent positive NAO related buoyancy forcing (Robson et al. 2012), whilst the warming and weakening of the SPG in the late 1990s and early 2000s coincided with a weakening AMOC related to a decline in deep convection in the Labrador Sea (Robson et al. 2016, and Fig. 7c).
- Observed AMOC changes estimated from various trans-basin monitoring arrays showed a rising trend from 2000 to about 2006 and a subsequent decline trend until about 2010). CMIP5 and CMIP6 models produce a forced weakening of the AMOC over the 2012–2017 period relative to 2004–2008, but at 26° N the multi-model mean response is substantially weaker than the observed AMOC decline over the same period. The discrepancy between the modelled multi-model mean and the RAPID observed AMOC changes has led studies to suggest that the observed weakening over 2004–2017 is largely due to internal variability and models do not support robust assessment of the role of anthropogenic forcing in the observed AMOC weakening between the mid-2000s and the mid-2010s. There is low confidence that anthropogenic forcing has influenced the observed changes in AMOC strength in the post-2004 period (e.g., Eyring et al. 2021).
- The AMOC is projected to weaken over the course of the twenty-first century in response to increasing concentrations of greenhouse gases. However, the magnitude and rate of decline as well as the impacts of this weakening are highly uncertain across models (Collins et al. 2013; Weijer et al. 2020; Arias et al. 2021; Bellomo et al. 2021; Fox-Kemper et al. 2021; Lee et al. 2021). Although climate model simulations archived in the Coupled Model Intercomparison Project (CMIP) do not project a shutdown of the AMOC (Fox-Kemper et al. 2021; Lee et al. 2021), it has been hypothesized that models may overlook this possibility (Liu et al. 2017; van Western et al. 2024). The collapse of the AMOC is nevertheless considered a ‘low-likelihood, high-impact’ outcome (e.g., Arias et al. 2021; Bellomo et al. 2023; van Western et al. 2024). Experiments with stabilised and negative emissions indicate that the AMOC may recover on timescales of 100 years or more (Schwinger et al. 2022; Jackson et al. 2023b).
- Future projections, till the end of the twenty-first century, with CMIP6 models show large divergence in the Arctic mean states and changes (Zanowski et al. 2021), although being in agreement on the increased poleward ocean heat transports and higher freshwater exports from the Arctic into the North Atlantic (Wang et al. 2023). Models indicate future potential large freshwater releases from the Arctic, which may disrupt the North Atlantic ocean circulation (both AMOC and SPG). However, observations do not yet provide consistent evidence of increased oceanic freshwater transport from the Arctic to the North Atlantic (Solomon et al. 2020; Lin et al. 2023).

## Outstanding questions

There is clear evidence that the North Atlantic climate system has changed over the past century (Sutton et al. 2017; Robson et al. 2018; Woollings et al. 2018) and we have strong indications that it will continue to change in the twenty-first century (Fox-Kemper et al. 2021; Harvey et al. 2020, 2023; Lee et al. 2021). However, there remains large uncertainty in the future changes and this is a challenge for developing robust approaches to adapt to the risks caused by climate change (Deser et al. 2012; Lehner et al 2023; Gillett 2024).

Uncertainty in regional climate projections may be usefully partitioned between internal variability, uncertainties in the forcing of the climate system (e.g. by anthropogenic emissions) and uncertainties in the responses to forcings (Hawkins and Sutton 2009). All three contributions to uncertainty are relevant to projections of North Atlantic climate change. There are substantial differences in the characteristics of internal variability simulated by different climate models, and the real world characteristics, particularly on decadal and longer timescales, are not known. Future forcing of the climate system is inherently uncertain due to ignorance of future human behaviour. However, it has been shown recently that the range of possible scenarios can be constrained, particularly in the near future of the next few decades (Beckage et al. 2018; 2022; Otto et al. 2020; Moore et al. 2022; Lehner et al. 2023). In addition to considering an appropriate range of scenarios for greenhouse gas emissions, uncertain anthropogenic aerosol emissions have been repeatedly highlighted as a major source of uncertainty in future climate projections (Myhre et al. 2013; Eyring et al. 2021; Szopa et al. 2021; Fiedler et al. 2023; Wilcox et al. 2023); this includes the necessity to consider uncertainty of different types of aerosol emissions.

Perhaps the most important scientific challenge is to advance understanding of the responses of regional atmosphere and ocean circulations to both anthropogenic and natural forcings (Collins et al. 2013; Harvey et al. 2014; Voigt and Shaw 2015; Ceppi and Shepherd 2017; Grise and Davis 2020; Bellomo et al. 2021; Lee et al. 2021; Robson et al. 2022; Shaw et al. 2024). There remain substantial differences between the regional responses simulated by different climate models, and there is a lack of understanding of the key processes involved. Large ensembles provide a valuable opportunity to better quantify and investigate model differences (Deser et al. 2020; Smith et al. 2022b). However, significant biases in the mean climates simulated by models add further uncertainty to the interpretation of model results (Iqbal et al. 2018; Lee et al. 2018; Harvey et al. 2020, 2023; Weijer et al. 2020; Fox-Kemper et al. 2021; Jackson et al. 2023a, b; McCarthy and Caesar 2023). Some recent studies have suggested that improved resolution of ocean fronts and the overlying atmosphere could significantly alter the nature of ocean–atmosphere coupling and improve model simulated SST biases over the North Atlantic (Smirnov et al. 2015; Siqueira and Kirtman 2016; Parfitt et al. 2017; Lee et al. 2018; Docquier et al. 2019; Athanasiadis et al. 2022; Moreno-Chamarro et al. 2022; Tsartsali et al. 2022). The extent to which higher resolution climate models change responses to different forcings needs to be assessed more thoroughly.

## Supplementary Information

Below is the link to the electronic supplementary material.

Supplementary file1 (DOCX 1387 KB)

## Data Availability

ERA5 reanalysis is available at https://climate.copernicus.eu/climate-reanalysis. CRUTS4.06 data is available at https://catalogue.ceda.ac.uk/uuid/e0b4e1e56c1c4460b796073a31366980.

## Code availability

Analysis codes are available upon request from the corresponding author BD (b.dong@reading.ac.uk).

## References

[CR1] An S-I, Shin J, Yeh S-W et al (2021) Global cooling hiatus driven by an AMOC overshoot in a carbon dioxide removal scenario. Earth’s Future 9:e2021EF002165. 10.1029/2021EF002165

[CR2] Andrews MB, Knight JR, Gray LJ (2015) A simulated lagged response of the North Atlantic Oscillation to the solar cycle over the period 1960–2009. Environ Res Lett 10(5):054022

[CR3] Andrews MB, Ridley JK, Wood RA et al (2020) Historical simulations with HadGEM3-GC3. 1 for CMIP6. J Adv Model Earth Syst 12(6):e2019MS001995

[CR4] Arias PA et al (2021) Technical summary. In: Masson-Delmotte V, Zhai P, Pirani A, Connors SL, Péan C, Berger S, Caud N, Chen Y, Goldfarb L, Gomis MI, Huang M, Leitzell K, Lonnoy E, Matthews JBR, Maycock TK, Waterfield T, Yelekçi O, Yu R, Zhou B (eds) Climate Change 2021: The physical science basis. Contribution of Working Group I to the Sixth Assessment Report of the Intergovernmental Panel on Climate Change. Cambridge University Press, Cambridge, pp. 33−144

[CR5] Armitage TW, Bacon S, Ridout AL et al (2017) Arctic Ocean surface geostrophic circulation 2003–2014. Cryosphere 11(4):1767–1780. 10.5194/tc-11-1767-2017

[CR6] Armitage TW, Manucharyan K, Petty GE et al (2020) Enhanced eddy activity in the Beaufort Gyre in response to sea ice loss. Nat Commun 11(1):761. 10.1038/s41467-020-14449-z 32029737 10.1038/s41467-020-14449-z PMC7005044

[CR7] Asbjørnsen H, Årthun M, Skagseth Ø, Eldevik T (2020) Mechanisms underlying recent Arctic atlantification. Geophys Res Lett 47(15):e2020GL088036

[CR8] Asbjørnsen H, Årthun M (2023) Deconstructing future AMOC decline at 26.5 N. Geophys Res Lett 50(14):e2023GL103515

[CR9] Athanasiadis PJ, Yeager S, Kwon Y-O, Bellucci A, Smith DW, Tibaldi S (2020) Decadal predictability of North Atlantic blocking and the NAO. NPJ Clim Atmos Sci 3(1):20. 10.1038/s41612-020-0120-6

[CR10] Athanasiadis PJ, Ogawa F, Omrani NE et al (2022) Mitigating climate biases in the midlatitude North Atlantic by increasing model resolution: SST gradients and their relation to blocking and the jet. J Clim 35(21):6985–7006

[CR11] Ba J, Keenlyside NS, Latif M et al (2014) A multi-model comparison of Atlantic multidecadal variability. Clim Dyn 43:2333–2348

[CR12] Bakker P, Schmittner A, Lenaerts JTM et al (2016) Fate of the Atlantic meridional overturning circulation: strong decline under continued warming and Greenland melting. Geophys Res Lett 43(23):12–252

[CR13] Baker JA, Bell MJ, Jackson LC et al (2023) Overturning pathways control AMOC weakening in CMIP6 models. Geophys Res Lett 50(14):e2023GL103381

[CR14] Barriopedro D, García-Herrera R, Huth R (2008) Solar modulation of Northern Hemisphere winter blocking. J Geophys Res Atmos. 10.1029/2008JD009789

[CR15] Barriopedro D, Ayarzagüena B, García-Burgos M, García-Herrera R (2023) A multi-parametric perspective of the North Atlantic eddy-driven jet. Clim Dyn 61(1–2):375–397

[CR16] Barnes EA, Polvani L (2013) Response of the midlatitude jets, and of their variability, to increased greenhouse gases in the CMIP5 models. J Clim 26:7117–7135. 10.1175/JCLI-D-12-00536.1

[CR17] Barnes EA, Screen JA (2015) The impact of Arctic warming on the midlatitude jet-stream: Can it? Has it? Will it? Wiley Interdiscip Rev Clim Change 6(3):277–286. 10.1002/wcc.337

[CR18] Beckage B, Gross LJ, Lacasse K et al (2018) Linking models of human behavior and climate alters projected climate change. Nat Clim Chang 8(1):79–84. 10.1038/s41558-017-0031-7

[CR19] Beckage B, Moore FC, Lacasse K (2022) Incorporating human behavior into Earth system modelling. Nat Hum Behav 6(11):1493–1502. 10.1038/s41562-022-01478-5 36385182 10.1038/s41562-022-01478-5

[CR20] Belkin IM, Levitus S, Antonov J, Malmberg SA (1998) “Great salinity anomalies” in the North Atlantic. Prog Oceanogr 41(1):1–68

[CR21] Belkin IM (2004) Propagation of the “Great Salinity Anomaly” of the 1990s around the northern North Atlantic. Geophys Res Lett. 10.1029/2003GL019334

[CR22] Bellomo K, Murphy LN, Cane MA et al (2018) Historical forcings as main drivers of the Atlantic multidecadal variability in the CESM large ensemble. Clim Dyn 50:3687–3698

[CR23] Bellomo K, Angeloni M, Corti S, von Hardenberg J (2021) Future climate change shaped by inter-model differences in Atlantic meridional overturning circulation response. Nat Commun 12(1):3659 34135324 10.1038/s41467-021-24015-w PMC8209213

[CR24] Bengtsson L, Hodges KI, Roeckner E (2006) Storm tracks and climate change. J Clim 19(15):3518–3543

[CR25] Bengtsson L, Hodges KI, Keenlyside N (2009) Will extratropical storms intensify in a warmer climate? J Clim 22(9):2276–2301

[CR26] Berthou S, Renshaw R, Smyth T et al (2024) Exceptional atmospheric conditions in June 2023 generated a northwest European marine heatwave which contributed to breaking land temperature records. Commun Earth Environ 5(1):287

[CR27] Bihlo A (2021) A generative adversarial network approach to (ensemble) weather prediction. Neural Netw 139:1–16. 10.1016/j.neunet.2021.02.003 33662648 10.1016/j.neunet.2021.02.003

[CR28] Bilbao R, Ortega P, Swingedouw D et al (2024) Impact of volcanic eruptions on CMIP6 decadal predictions: a multi-model analysis. Earth Syst Dyn 15(2):501–525

[CR29] Bingham R, Hughes CW (2009) Signature of the Atlantic meridional overturning circulation in sea level along the east coast of North America. Geophys Res Lett 36:L02603

[CR30] Blackport R, Screen JA (2020) Insignificant effect of Arctic amplification on the amplitude of midlatitude atmospheric waves. Sci Adv 6(8):eaay880. 10.1126/sciadv.aay2880 10.1126/sciadv.aay2880 PMC7030927 32128402

[CR31] Blackport R, Fyfe JC (2022) Climate models fail to capture strengthening wintertime North Atlantic jet and impacts on Europe. Sci Adv 8(45):eabn3112 36367934 10.1126/sciadv.abn3112 PMC9651855

[CR32] Boers N (2021) Observation-based early-warning signals for a collapse of the Atlantic Meridional Overturning Circulation. Nat Clim Chang 11(8):680–688

[CR33] Böning C, Scheinert M, Dengg J, Biastoch A, Funk A (2006) Decadal variability of subpolar gyre transport and its reverberation in the North Atlantic overturning. Geophys Res Lett. 10.1029/2006GL026906

[CR34] Booth BBB, Dunstone NJ, Halloran PR et al (2012) Aerosols implicated as a prime driver of twentieth-century North Atlantic climate variability. Nature 484:228–232 22498628 10.1038/nature10946

[CR35] Borchert LF, Pohlmann H, Baehr J et al (2019) Decadal predictions of the probability of occurrence for warm summer temperature extremes. Geophys Res Lett 46(23):14042–14051

[CR36] Borchert LF, Menary MB, Swingedouw D et al (2021) Improved decadal predictions of North Atlantic subpolar gyre SST in CMIP6. Geophys Res Lett 48(3):e2020GL091307

[CR37] Boucher O, Randall D, Artaxo P et al (2013) Clouds and aerosols. In: Climate change 2013: The physical science basis. Contribution of working group I to the fifth assessment report of the intergovernmental panel on climate change. Cambridge University Press, pp 571–657

[CR38] Boulton CA, Allison LC, Lenton TM (2014) Early warning signals of Atlantic Meridional Overturning Circulation collapse in a fully coupled climate model. Nat Commun 5(1):5752 25482065 10.1038/ncomms6752 PMC4268699

[CR39] Bracegirdle TJ, Lu H, Eade R, Woollings T (2018) Do CMIP5 models reproduce observed low-frequency North Atlantic jet variability? Geophys Res Lett 45:7204–7212. 10.1029/2018GL078965

[CR40] Brayshaw DJ, Hoskins B, Blackburn M (2011) The basic ingredients of the North Atlantic storm track. Part II: sea surface temperatures. J Atmos Sci 68:1784–1805

[CR41] Buckley MW, Marshall J (2016) Observations, inferences, and mechanisms of the Atlantic Meridional overturning circulation: a review. Rev Geophys. 10.1002/2015RG000493

[CR42] Budikova D, Ford TW, Ballinger TJ (2017) Connections between north-central United States summer hydroclimatology and Arctic sea ice variability. Int J Climatol 37(12):4434–4450

[CR43] Butler AH, Thompson DW, Heikes R (2010) The steady-state atmospheric circulation response to climate change–like thermal forcings in a simple general circulation model. J Clim 23(13):3474–3496

[CR44] Caesar L, Rahmstorf S, Robinson A, Feulner G, Saba V (2018) Observed fingerprint of a weakening Atlantic Ocean overturning circulation. Nature 556(7700):191–196. 10.1038/s41586-018-0006-5 29643485 10.1038/s41586-018-0006-5

[CR45] Caesar L, McCarthy GD, Thornalley DJR et al (2021) Current Atlantic Meridional Overturning Circulation weakest in last millennium. Nat Geosci 14:118–120. 10.1038/s41561-021-00699-z

[CR46] Cai W, Bi D, Church J et al (2006) Pan-oceanic response to increasing anthropogenic aerosols: impacts on the Southern Hemisphere oceanic circulation. Geophys Res Lett 33:L21707. 10.1029/2006GL027513

[CR47] Carmack E, Yamamoto-Kawai M, Haine T et al (2016) Freshwater and its role in the Arctic Marine System: sources, disposition, storage, export, and physical and biogeochemical consequences in the Arctic and global oceans. J Geophys Res-Biogeo 121:675–717. 10.1002/2015JG003140

[CR48] Ceppi P, Shepherd TG (2017) Contributions of climate feedbacks to changes in atmospheric circulation. J Clim 30(22):9097–9118

[CR49] Chafik L, Nilsen JEØ, Dangendorf S, Reverdin G, Frederikse T (2019) North Atlantic Ocean circulation and decadal sea level change during the altimetry era. Sci Rep 9(1):1041 30705311 10.1038/s41598-018-37603-6 PMC6355806

[CR50] Chang EKM, Ma C-G, Zheng C, Yau AMW (2016) Observed and projected decrease in Northern Hemisphere extratropical cyclone activity in summer and its impacts on maximum temperature. Geophys Res Lett 43(5):2200–2208. 10.1002/2016GL068172

[CR51] Charlton-Perez AJ, Baldwin MP, Birner T et al (2013) On the lack of stratospheric dynamical variability in low-top versions of the CMIP5 models. J Geophys Res Atmos 118:2494–2505

[CR52] Chemke R, Zanna L, Orbe C, Sentman LT, Polvani LM (2022) The future intensification of the North Atlantic winter storm track: the key role of dynamic ocean coupling. J Clim 35(8):2407–2421

[CR53] Chen S, Wu R, Chen W, Hu K, Yu B (2020) Structure and dynamics of a springtime atmospheric wave train over the North Atlantic and Eurasia. Clim Dyn 54:5111–5126

[CR54] Cheng W, Chiang JCH, Zhang D (2013) Atlantic meridional overturning circulation (AMOC) in CMIP5 models: RCP and historical simulations. J Clim 26(18):7187–7197. 10.1175/jcli-d-12-00496.1

[CR55] Chiodo G, Oehrlein J, Polvani LM, Fyfe JC, Smith AK (2019) Insignificant influence of the 11-year solar cycle on the North Atlantic Oscillation. Nat Geosci 12(2):94–99

[CR56] Ciasto LM, Li C, Wettstein JJ, Kvamstø NG (2016) North Atlantic storm-track sensitivity to projected sea surface temperature: local versus remote influences. J Clim 29(19):6973–6991

[CR57] Cohen JL, Furtado JC, Barlow MA, Alexeev VA, Cherry JE (2012) Arctic warming, increasing snow cover and widespread boreal winter cooling. Environ Res Lett 7:014007. 10.1088/1748-9326/7/1/014007

[CR58] Cohen J, Screen JA, Furtado JC, Barlow M, Whittleston D, Coumou D, Francis J, Dethloff K, Entekhabi D, Overland J, Jones J (2014) Recent Arctic amplification and extreme mid-latitude weather. Nat Geosci 7:627–637. 10.1038/ngeo2234

[CR59] Cohen J, Pfeiffer K, Francis J (2018) Warm Arctic episodes linked with increased frequency of extreme winter weather in the United States. Nat Commun 9:869. 10.1038/s41467-018-02992-9 29535297 10.1038/s41467-018-02992-9 PMC5849726

[CR60] Cohen J, Zhang X, Francis J et al (2020) Divergent consensuses on Arctic amplification influence on midlatitude severe winter weather. Nat Clim Change 10:20–29. 10.1038/s41558-019-0662-y

[CR61] Colfescu I, Christensen H, Gagne DJ (2024) A machine learning-based approach to quantify ENSO sources of predictability. Geophys Res Lett 51(13):e2023GL105194

[CR62] Collins M, Sinha B (2003) Predictability of decadal variations in the thermohaline circulation and climate. Geophys Res Lett 30(6)

[CR63] Collins M, Botzet M, Carril AF et al (2006) Interannual to decadal climate predictability in the North Atlantic: a multimodel-ensemble study. J Clim 19(7):1195–1203

[CR64] Collins M, Knutti R, Arblaster J et al (2013) Long-term climate change: projections, commitments and irreversibility. In: Stocker TF, Qin D, Plattner G-K, Tignor M, Allen SK, Boschung J, Nauels A, Xia Y, Bex V, Midgley PM (eds) Climate Change 2013: The Physical Science Basis. Contribution of Working Group I to the Fifth assessment report of the intergovernmental panel on climate change. Cambridge University Press, Cambridge, United Kingdom and New York

[CR65] Cotterill DF, Pope JO, Stott PA (2023) Future extension of the UK summer and its impact on autumn precipitation. Clim Dyn 60(5–6):1801–1814

[CR66] Couldrey MP, Gregory JM, Boeira Dias F et al (2021) What causes the spread of model projections of ocean dynamic sea-level change in response to greenhouse gas forcing? Clim Dyn 56(1):155–187

[CR67] Coumou D, Rahmstorf S (2012) A decade of weather extremes. Nat Clim Chang 2(7):491–496

[CR68] Coumou D, Petoukhov V, Rahmstorf S, Petri S, Schellnhuber HJ (2014) Quasi-resonant circulation regimes and hemispheric synchronization of extreme weather in boreal summer. Proc Natl Acad Sci 111(34):12331–12336 25114245 10.1073/pnas.1412797111 PMC4151761

[CR69] Coumou D, Lehmann J, Beckmann J (2015) The weakening summer circulation in the Northern Hemisphere mid-latitudes. Science 348(6232):324–327. 10.1126/science.1261768 25765067 10.1126/science.1261768

[CR70] Coumou D, Di Capua G, Vavrus S, Wang L, Wang S (2018) The influence of Arctic amplification on mid-latitude summer circulation. Nat Commun 9(1):1–12. 10.1038/s41467-018-05256-8 30127423 10.1038/s41467-018-05256-8 PMC6102303

[CR71] Czaja A, Frankignoul C, Minobe S, Vannière B (2019) Simulating the midlatitude atmospheric circulation: what might we gain from high-resolution modeling of air-sea interactions? Current Clim Change Rep 5:390–406

[CR72] Dai A, Song M (2020) Little influence of Arctic amplification on mid-latitude climate. Nat Clim Chang 10(3):231–237. 10.1038/s41558-020-0694-3

[CR73] Dai A (2022) Arctic amplification is the main cause of the Atlantic meridional overturning circulation weakening under large CO_2_ increases. Clim Dyn 58(11–12):3243–3259

[CR74] DallaSanta K, Gerber EP, Toohey M (2019) The circulation response to volcanic eruptions: the key roles of stratospheric warming and Eddy interactions. J Climate 32:1101–1120. 10.1175/JCLI-D-18-0099.1

[CR75] Davini P, von Hardenberg J, Corti S (2015) Tropical origin for the impacts of the Atlantic multidecadal variability on the Euro-Atlantic climate. Environ Res Lett 10:094010. 10.1088/1748-9326/10/9/094010

[CR76] Delworth T, Manabe M, Stouffer RJ (1993) Interdecadal variations of the thermohaline circulation in a coupled ocean-atmosphere model. J Clim 6(11):1993–2011. 10.1175/1520-0442(1993)0062.0.CO;2

[CR77] Delworth T, Dixon KW (2006) Have anthropogenic aerosols delayed a greenhouse gas-induced weakening of the North Atlantic thermohaline circulation? Geophys Res Lett 33:L02606. 10.1029/2005GL024980

[CR78] Delworth TL, Zeng F (2012) Multicentennial variability of the Atlantic meridional overturning circulation and its climatic influence in a 4000 year simulation of the GFDL CM2.1 climate model. Geophys Res Lett 39:13702. 10.1029/2012GL052107

[CR79] Deng KQ, Azorin-Molina C, Yang S et al (2022) Shifting of summertime weather extremes in Western Europe during 2012–2020. Adv Clim Chang Res 13(2):218–227

[CR80] Desbruyères D, Chafik L, Maze G (2021) A shift in the ocean circulation has warmed the subpolar North Atlantic Ocean since 2016. Commun Earth Environ 2(1):48

[CR81] Deser C, Phillips A, Bourdette V, Teng H (2012) Uncertainty in climate change projections: the role of internal variability. Clim Dyn 38:527–546. 10.1007/s00382-010-0977-x

[CR82] Deser C, Hurrell JW, Phillips AS (2017) The role of the North Atlantic Oscillation in European climate projections. Clim Dyn 49:3141–3157

[CR83] Deser C, Phillips AS, Simpson IR et al (2020) Isolating the evolving contributions of anthropogenic aerosols and greenhouse gases: a new CESM1 large ensemble community resource. J Clim 33:7835–7858. 10.1175/JCLI-D-20-0123.1

[CR84] Dickson RR, Meincke J, Malmberg SA, Lee AJ (1988) The “great salinity anomaly” in the northern North Atlantic 1968–1982. Prog Oceanogr 20(2):103–151

[CR85] Ditlevsen P, Ditlevsen S (2023) Warning of a forthcoming collapse of the Atlantic meridional overturning circulation. Nat Commun 14(1):4254 37491344 10.1038/s41467-023-39810-w PMC10368695

[CR86] Doblas-Reyes FJ, Sorensson A, Almazroui M et al (2021) Linking global to regional climate change. In: Masson-Delmotte V et al (eds) Climate change 2021: the physical science basis. Contribution of working group I to the sixth assessment report of the intergovernmental panel on climate change. Cambridge University Press, Cambridge, United Kingdom and New York, NY, USA, pp 1363–1512

[CR87] Docquier D, Grist JP, Roberts MJ et al (2019) Impact of model resolution on Arctic sea ice and North Atlantic Ocean heat transport. Clim Dyn 53:4989–5017. 10.1007/s00382-019-04840-y

[CR88] Dong BW, Sutton RT (2005) Mechanism of interdecadal thermohaline circulation variability in a coupled ocean-atmosphere GCM. J Clim 18:1117–1135. 10.1175/JCLI3328.1

[CR89] Dong BW, Sutton RT (2007) Enhancement of ENSO variability by a weakened Atlantic thermohaline circulation in a coupled GCM. J Clim 20(19):4920–4939. 10.1175/JCLI4284.1

[CR90] Dong BW, Sutton RT, Scaife AA (2006) Multidecadal modulation of El Nino-Southern Oscillation (ENSO) variance by Atlantic Ocean sea surface temperatures. Geophys Res Lett 33:L08705. 10.1029/2006GL025766

[CR91] Dong BW, Sutton RT, Woollings T, Hodges K (2013) Variability of the North Atlantic summer storm track: mechanisms and impacts on European climate. Environ Res Lett 8(3):034037

[CR92] Dong B, Sutton RT, Shaffrey L (2017) Understanding the rapid summer warming and changes in temperature extremes since the mid-1990s over Western Europe. Clim Dyn. 10.1007/s00382-016-3158-8

[CR93] Dong BW, Sutton RT (2021) Recent trends in summer atmospheric circulation in the North Atlantic/European region: is there a role for anthropogenic aerosols? J Clim. 10.1175/JCLI-D-20-0665.1

[CR94] Dong BW, Sutton RT, Shaffrey L, Harvey B (2022) Recent decadal weakening of the summer Eurasian westerly jet attributable to anthropogenic aerosol emissions. Nat Commun. 10.1038/s41467-022-28816-5 10.1038/s41467-022-28816-5 PMC8894405 35241666

[CR95] Drews A, Huo W, Matthes K, Kodera K, Kruschke T (2022) The Sun’s role in decadal climate predictability in the North Atlantic. Atmos Chem Phys 22:7893–7904. 10.5194/acp-22-7893-2022

[CR96] Drijfhout S, van Oldenborgh GJ, Cimatoribus A (2012) Is a decline of AMOC causing the warming hole above the North Atlantic in observed and modeled warming patterns? J Clim 25:8373–8379

[CR97] Duchez A, Courtois P, Harris E et al (2016a) Potential for seasonal prediction of Atlantic sea surface temperatures using the RAPID array at 26° N. Clim Dyn 46:3351–3370. 10.1007/s00382-015-2918-1

[CR98] Duchez A, Frajka-Williams E, Josey SA et al (2016b) Drivers of exceptionally cold North Atlantic Ocean temperatures and their link to the 2015 European heat wave. Environ Res Lett 11(7):074004

[CR99] Dueben PD, Bauer P (2018) Challenges and design choices for global weather and climate models based on machine learning. Geosci Model Dev 11(10):3999–4009. 10.5194/gmd-11-3999-2018

[CR100] Dunn-Sigouin E, Son SW (2013) Northern Hemisphere blocking frequency and duration in the CMIP5 models. J Geophys Res 118:1179–1188

[CR101] Dunstone N, Smith D, Scaife A et al (2016) Skilful predictions of the Winter North Atlantic Oscillation one year ahead. Nat Geosci 9:809–814

[CR102] Dunstone N, Smith D, Hardiman SC et al (2023) Skilful predictions of the summer North Atlantic Oscillation. Commun Earth Environ 4(1):409

[CR103] Eade R, Smith D, Scaife A et al (2014) Do seasonal-to-decadal climate predictions underestimate the predictability of the real world? Geophys Res Lett 41(15):5620–5628 25821271 10.1002/2014GL061146 PMC4373130

[CR104] Eade R, Stephenson DB, Scaife AA et al (2022) Quantifying the rarity of extreme multi-decadal trends: how unusual was the late twentieth century trend in the North Atlantic Oscillation? Clim Dyn 58:1555–1568. 10.1007/s00382-021-05978-4

[CR105] Eden C, Willebrand J (2001) Mechanism of interannual to decadal variability of the North Atlantic circulation. J Clim 14(10):2266–2280. 10.1175/1520-0442(2001)0142.0.CO;2

[CR106] England M, Polvani LM, Sun L (2018) Contrasting the Antarctic and Arctic atmospheric responses to projected sea ice loss in the late twenty-first century. J Clim 31:6353–6370

[CR107] Eyring V, Gillett NP, Rao KMA et al (2021) Human influence on the climate system. In: Masson-Delmotte V et al(eds) Climate change 2021: the physical science basis. Contribution of Working Group I to the Sixth Assessment Report of the Intergovernmental Panel on Climate Change. Cambridge University Press, Cambridge, United Kingdom and New York, NY, USA, pp 423–552

[CR108] Famooss Paolini L, Athanasiadis PJ, Ruggieri P, Bellucci A (2022) The atmospheric response to meridional shifts of the Gulf Stream SST front and its dependence on model resolution. J Clim 35(18):6007–6030

[CR109] Feldstein SB (2000) The timescale, power spectra, and climate noise properties of teleconnection patterns. J Clim 13(24):4430–4440

[CR110] Fiedler S, van Noije T, Smith CJ et al (2023) Historical changes and reasons for model differences in anthropogenic aerosol forcing in CMIP6. Geophys Res Lett 50(15):e2023GL104848

[CR111] Florindo-López C, Bacon S, Aksenov Y et al (2020) Arctic Ocean and Hudson Bay freshwater exports: New estimates from seven decades of hydrographic surveys on the Labrador Shelf. J Clim 33(20):8849–8868. 10.1175/JCLI-D-19-0083.1

[CR112] Folland CK, Palmer TN, Parker DE (1986) Sahel rainfall and worldwide sea temperatures, 1901–85. Nature 320(6063):602–607. 10.1038/320602a0

[CR113] Fox-Kemper B, Hewitt HT, Xiao C et al (2021) Ocean, cryosphere and sea level change. In: Masson-Delmotte V, Zhai P, Pirani A, Connors SL, Péan C, Berger S, Caud N, Chen Y, Goldfarb L, Gomis MI, Huang M, Leitzell K, Lonnoy E, Matthews JBR, Maycock TK, Waterfield T, Yelekçi O, Yu R, Zhou B (eds) Climate change 2021: the physical science basis. Contribution of Working Group I to the Sixth Assessment Report of the Intergovernmental Panel on Climate Change. Cambridge University Press, Cambridge, United Kingdom and New York, NY, USA, pp 1211–1362

[CR114] Francis JA, Vavrus SJ (2012) Evidence linking Arctic amplification to extreme weather in mid-latitudes. Geophys Res Lett 39:L06801. 10.1029/2012GL051000

[CR115] Francis JA, Vavrus SJ (2015) Evidence for a wavier jet stream in response to rapid Arctic warming. Environ Res Lett 10(1):14005

[CR116] Francis JA (2017) Why are Arctic linkages to extreme weather still up in the air. Bull Am Meteorol Soc 98(12):2551–2557. 10.1175/BAMS-D-17-0006.1

[CR117] Frankignoul C, Gastineau G, Kwon YO (2015) Wintertime atmospheric response to North Atlantic ocean circulation variability in a climate model. J Clim 28(19):7659–7677. 10.1175/JCLI-D-15-0007.1

[CR118] Friedlingstein P, Meinshausen M, Arora VK et al (2014) Uncertainties in CMIP5 climate projections due to carbon cycle feedbacks. J Clim 27(2):511–526. 10.1175/JCLI-D-12-00579.1

[CR119] Gastineau G, Frankignoul C (2012) Cold-season atmospheric response to the natural variability of the Atlantic meridional overturning circulation. Clim Dyn 39:37–57

[CR120] Gastineau G, D’Andrea F, Frankignoul C (2013) Atmospheric response to the North Atlantic Ocean variability on seasonal to decadal time scales. Clim Dyn 40:2311–2330

[CR121] Gervais M, Shaman J, Kushnir Y (2019) Impacts of the North Atlantic warming hole in future climate projections: mean atmospheric circulation and the North Atlantic jet. J Clim 32(10):2673–2689

[CR122] Ghosh R, Putrasahan D, Manzini E et al (2023) Two distinct phases of North Atlantic Eastern Subpolar Gyre and warming hole evolution under global warming. J Clim 36(6):1881–1894

[CR123] Giles KA, Laxon SW, Ridout AL et al (2012) Western Arctic Ocean freshwater storage increased by wind-driven spin-up of the Beaufort Gyre. Nat Geosci 5(3):194–197

[CR124] Gillett NP (2024) Halving of the uncertainty in projected warming over the past decade. npj Clim Atmos Sci 7(1):146

[CR125] Gordon EM, Barnes EA (2022) Incorporating uncertainty into a regression neural network enables identification of decadal state-dependent predictability in CESM2. Geophys Res Lett 49(15):e2022GL098635

[CR126] Goldenberg SB, Landsea CW, Mestas-Nuñez AM, Gray WM (2001) The recent increase in Atlantic hurricane activity: causes and implications. Science 293(5529):474–479. 10.1126/science.1060040 11463911 10.1126/science.1060040

[CR128] Graff LS, LaCasce JH (2012) Changes in the extratropical storm tracks in response to changes in SST in an AGCM. J Clim 25(6):1854–1870

[CR127] Gray LJ, Beer J, Geller M et al (2010) Solar influences on climate. Rev Geophys 48:RG4001. 10.1029/2009RG000282

[CR129] Gray LJ, Scaife AA, Mitchell DM et al (2013) A lagged response to the 11 year solar cycle in observed winter Atlantic/European weather patterns. J Geophys Res Atmos 118(24):13–405

[CR130] Gray LJ, Wollings TJ, Andrews M, Knight J (2016) Eleven-year solar cycle signal in the NAO and Atlantic/European blocking. Quart J Roy Meteor Soc 142:1890–1903. 10.1002/qj.2782

[CR131] Gregory JM, Dixon KW, Stouffer RJ et al (2005) A model intercomparison of changes in the Atlantic thermohaline circulation in response to increasing atmospheric CO2 concentration. Geophys Res Lett. 10.1029/2005GL023209

[CR132] Griffies SM, Bryan K (1997) Predictability of North Atlantic multidecadal climate variability. Science 275(5297):181–184 8985005 10.1126/science.275.5297.181

[CR133] Grise KM, Polvani LM (2016) Is climate sensitivity related to dynamical sensitivity? J Geophys Res: Atmos 121(10):5159–5176

[CR134] Grise KM, Davis SM (2020) Hadley cell expansion in CMIP6 models. Atmos Chem Phys 20(9):5249–5268

[CR135] Grist JP, Josey SA, Marsh R et al (2010) The roles of surface heat flux and ocean heat transport convergence in determining Atlantic Ocean temperature variability. Ocean Dyn 60:771–790

[CR136] Grist JP, Sinha B, Hewitt HT et al (2019) Re-emergence of North Atlantic subsurface ocean temperature anomalies in a seasonal forecast system. Clim Dyn 53:4799–4820. 10.1007/s00382-019-04826-w

[CR137] Gulev SK, Latif M, Keenlyside N, Park W, Koltermann KP (2013) North Atlantic Ocean control on surface heat flux on multidecadal timescales. Nature 499(7459):464–467 23887431 10.1038/nature12268

[CR138] Gulev SK, Thorne PW, Ahn J et al (2021) Changing state of the climate system. In: Masson-Delmotte V, Zhai P, Pirani A, Connors SL, Péan C, Berger S, Caud N, Chen Y, Goldfarb L, Gomis MI, Huang M, Leitzell K, Lonnoy E, Matthews JBR, Maycock TK, Waterfield T, Yelekçi O, Yu R, Zhou B (eds.) Climate change 2021: the physical science basis. Contribution of Working Group I to the Sixth Assessment Report of the Intergovernmental Panel on Climate Change. Cambridge University Press, Cambridge, United Kingdom and New York, NY, USA, pp 287–422

[CR139] Haak H, Jungclaus J, Mikolajewicz U, Latif M (2003) Formation and propagation of great salinity anomalies. Geophys Res Lett 30(9)

[CR140] Haarsma RJ, Selten F, van Oldenborgh GJ (2013) Anthropogenic changes of the thermal and zonal flow structure over Western Europe and Eastern North Atlantic in CMIP3 and CMIP5 models. Clim Dyn 41:2577–2588

[CR141] Haigh JD (1996) The impact of solar variability on climate. Science 272:981–984 8662582 10.1126/science.272.5264.981

[CR142] Haine TW, Curry B, Gerdes R et al (2015) Arctic freshwater export: status, mechanisms, and prospects. Global Planet Change 125:13–35. 10.1016/j.gloplacha.2014.11.013

[CR143] Häkkinen S, Rhines PB (2004) Decline of subpolar North Atlantic circulation during the 1990s. Science 304(5670):555–559 15087505 10.1126/science.1094917

[CR144] Häkkinen S, Rhines PB, Worthen DL (2013) Northern North Atlantic sea surface height and ocean heat content variability. J Geophys Res Oceans 118(7):3670–3678

[CR145] Hall R, Erdélyi R, Hanna E, Jones JM, Scaife AA (2015) Drivers of North Atlantic polar front jet stream variability. Int J Climatol 35:1697–1720. 10.1002/joc.4121

[CR146] Hall RJ, Wei HL, Hanna E (2019) Complex systems modelling for statistical forecasting of winter North Atlantic atmospheric variability: a new approach to North Atlantic seasonal forecasting. Q J R Meteorol Soc 145:2568–2585

[CR147] Hallam S, Marsh R, Josey SA et al (2019) Ocean precursors to the extreme Atlantic 2017 hurricane season. Nat Commun 10(1):896 30796207 10.1038/s41467-019-08496-4 PMC6384944

[CR148] Hallam S, Josey SA, McCarthy GD, Hirschi JJM (2022) A regional (land–ocean) comparison of the seasonal to decadal variability of the Northern Hemisphere jet stream 1871–2011. Clim Dyn 59(7–8):1897–1918

[CR149] Ham YG, Kim JH, Luo JJ (2019) Deep learning for multi-year ENSO forecasts. Nature 573(7775):568–572 31534218 10.1038/s41586-019-1559-7

[CR150] Hanna E, Cropper TE, Jones PD, Scaife AA, Allan R (2015) Recent seasonal asymmetric changes in the NAO (a marked summer decline and increased winter variability) and associated changes in the AO and Greenland Blocking Index. Int J Climatol. 10.1002/joc.4157

[CR151] Hanna E, Cropper TE, Hall RJ, Cappelen J (2016) Greenland Blocking Index 1851–2015: a regional climate change signal. Int J Climatol 36:4847–4861

[CR152] Hanna E, Hall RJ, Overland JE (2017) Can Arctic warming influence UK weather? Weather 72:346–352

[CR153] Hanna E, Cropper TE, Hall RJ, Cornes RC, Barriendos M (2022) Extended North Atlantic Oscillation and Greenland Blocking indices 1800–2020 from new meteorological analysis. Atmosphere 13:436. 10.3390/atmos13030436

[CR154] Hardiman SC, Dunstone NJ, Scaife AA et al (2022) Missing eddy feedback may explain weak signal-to-noise ratios in climate predictions. Npj Clim Atmos Sci 5:1–8. 10.1038/s41612-022-00280-4

[CR155] Harris I, Jones PD, Osborn TJ, Lister DH (2014) Updated high-resolution grids of monthly climatic observations—the CRU TS3.10 dataset. Int J Climatol 34:623–642. 10.1002/joc.3711

[CR156] Harvey BJ, Shafrey LC, Woollings TJ (2014) Equator-to-pole temperature differences and the extra-tropical storm track responses of the CMIP5 climate models. Clim Dyn 43:1171–1182

[CR157] Harvey BJ, Cook P, Shaffrey LC, Schiemann R (2020) The response of the northern hemisphere storm tracks and jet streams to climate change in the CMIP3, CMIP5, and CMIP6 climate models. J Geophys Res 125:e32701

[CR158] Harvey BJ, Hawkins E, Sutton RT (2023) Storylines for future changes of the North Atlantic jet and associated impacts on the UK. Int J Climatol

[CR159] Haskins RK, Oliver KI, Jackson LC, Wood RA, Drijfhout SS (2020) Temperature domination of AMOC weakening due to freshwater hosing in two GCMs. Clim Dyn 54:273–286

[CR160] Hassan T, Allen RJ, Liu W, Randles CA (2021) Anthropogenic aerosol forcing of the Atlantic meridional overturning circulation and the associated mechanisms in CMIP6 models. Atmos Chem Phys 21(8):5821–5846

[CR161] Hassan T, Allen RJ, Liu W et al (2022) Air quality improvements are projected to weaken the Atlantic meridional overturning circulation through radiative forcing effects. Commun Earth Environ 3(1):149

[CR162] Hatun H, Sandø AB, Drange H, Hansen B, Valdimarsson H (2005) Infuence of the Atlantic subpolar gyre on the thermohaline circulation. Science 309:1841–1844. 10.1126/science.1114777 16166513 10.1126/science.1114777

[CR163] Hawkins E, Sutton R (2009) The potential to narrow uncertainty in regional climate predictions. Bull Am Meteorol Soc 90:1095–1108

[CR164] Hawkins E, Smith RS, Allison LC et al (2011) Bistability of the Atlantic overturning circulation in a global climate model and links to ocean freshwater transport. Geophys Res Lett. 10.1029/2011GL047208

[CR165] Hausfather Z, Peters GP (2020) Emissions–the ‘business as usual’ story is misleading. Nature 577(7792):618–620 31996825 10.1038/d41586-020-00177-3

[CR166] Heinze C et al (2023) Reviews and syntheses: Abrupt ocean biogeochemical change under human-made climatic forcing–warming, acidification, and deoxygenation. Biogeosci. 10.5194/bg-2023-182

[CR167] Held IM (1975) Momentum transport by quasi-geostrophic eddies. J Atmos Sci 32:1494–1497

[CR168] Held IM (1993) Large-scale dynamics and global warming. Bull Am Meteor Soc 74(2):228–242

[CR169] Hermanson L, Bilbao R, Dunstone N et al (2020) Robust multiyear climate impacts of volcanic eruptions in decadal prediction systems. J Geophys Res: Atmos 125:e2019031739. 10.1029/2019JD031739

[CR170] Hermoso A, Riviere G, Harvey B, Methven J, Schemm S (2023) Modification of the winter North Atlantic jet stream due to anthropogenic climate change

[CR171] Herrera-Lormendez P, Douville H, Matschullat J (2023) European summer synoptic circulations and their observed 2022 and projected influence on hot extremes and dry spells. Geophys Res Lett 50(18):e2023GL104580

[CR172] Hersbach H, Bell B, Berrisford P et al (2020) The ERA5 global reanalysis. Q J R Meteorol Soc 146(730):1999–2049

[CR173] Heuzé C (2017) North Atlantic deep water formation and AMOC in CMIP5 models. Ocean Sci 13(4):609–622

[CR174] Hirschi JJM, Barnier B, Böning C et al (2020) The Atlantic meridional overturning circulation in high-resolution models. J Geophys Res: Oceans 125(4):e2019JC015522

[CR175] Hobbs WR, Willis JK (2012) Midlatitude North Atlantic heat transport: a time series based on satellite and drifter data. J Geophys Res: Oceans 117(C1)

[CR176] Holland MM, Bitz CM (2003) Polar amplification of climate change in coupled models. Clim Dyn 21(3):221–232

[CR177] Holliday NP, Bersch M, Berx B et al (2020) Ocean circulation causes the largest freshening event for 120 years in eastern subpolar North Atlantic. Nat Commun 11:585. 10.1038/s41467-020-14474-y 31996687 10.1038/s41467-020-14474-y PMC6989661

[CR178] Holton JR (1992) An introduction to dynamic meteorology, 3rd edn. Academic Press, New York

[CR179] Hoskins BJ, James IN, White GH (1983) The shape, propagation and mean-flow interaction of large scale weather systems. J Atmos Sci 40:1595–1612

[CR180] Hoskins B, Woollings T (2015) Persistent extratropical regimes and climate extremes. Curr Clim Change Rep 1(3):115–124

[CR181] Hu Y, Tao L, Liu J (2013) Poleward expansion of the Hadley circulation in CMIP5 simulations. Adv Atmos Sci 30:790–795

[CR182] Hurrell JW (1995) Decadal trends in the North Atlantic Oscillation: regional temperatures and precipitation. Science 269:676–679 17758812 10.1126/science.269.5224.676

[CR183] Ineson S, Scaife AA, Knight JR et al (2011) Solar forcing of winter climate variability in the Northern Hemisphere. Nat Geosci 4(11):753–757

[CR184] Iles C, Hegerl G (2017) Role of the North Atlantic oscillation in decadal temperature trends. Environ Res Lett 12:114010. 10.1088/1748-9326/aa9152

[CR185] Ionita M, Nagavciuc V, Kumar R, Rakovec O (2020) On the curious case of the recent decade, mid-spring precipitation deficit in central Europe. Npj Clim Atmos Sci 3(1):49

[CR186] Iqbal W, Leung WN, Hannachi A (2018) Analysis of the variability of the north Atlantic eddy-driven jet stream in cmip5. Clim Dyn 51(1–2):235–247

[CR187] Iwi AM, Hermanson L, Haines K, Sutton RT (2012) Mechanisms linking volcanic aerosols to the Atlantic meridional overturning circulation. J Clim 25(8):3039–3051

[CR188] Jackson LC, Biastoch A, Buckley MW et al (2022) The evolution of the North Atlantic Meridional overturning circulation since 1980. Nat Rev Earth Environ 3:241–254. 10.1038/s43017-022-00263-2

[CR189] Jackson LC, Hewitt HT, Bruciaferri D et al (2023a) Challenges simulating the AMOC in climate models. Phil Trans R Soc A 381:20220187. 10.1098/rsta.2022.0187 37866390 10.1098/rsta.2022.0187

[CR190] Jackson LC, Alastrué de Asenjo E, Bellomo K et al (2023b) Understanding AMOC stability: the North Atlantic hosing model intercomparison project. Geosci Model Dev Discuss. 10.5194/gmd-2022-277

[CR191] Jiang W, Gastineau G, Codron F (2021) Multicentennial variability driven by salinity exchanges between the Atlantic and the Arctic Ocean in a coupled climate model. J Adv Model Earth Syst 13(3):e2020MS002366

[CR192] Josey SA, Grist JP, Marsh RA (2009) Estimates of meridional overturning circulation variability in the North Atlantic from surface density flux fields. JGR–Oceans 114:C09022. 10.1029/2008JC005230

[CR193] Josey S, Hirschi JJM, Sinha B et al (2018) The recent Atlantic cold anomaly: causes, consequences and related phenomena. Ann Rev Mar Sci. 10.1146/annurev-marine-121916-063102 10.1146/annurev-marine-121916-063102 28934597

[CR194] Josey SA, Sinha B (2022) Sub-polar Atlantic Ocean mixed layer heat content variability is increasingly driven by an active Ocean. Nature Commun Earth Environ. 10.1038/s43247-022-00433-6

[CR195] Jüling A, Zhang X, Castellana D, Von Der Heydt AS, Dijkstra HA (2021) The Atlantic’s freshwater budget under climate change in the Community Earth System Model with strongly eddying oceans. Ocean Sci 17(3):729–754

[CR196] Jungclaus JH, Haak H, Latif M, Mikolajewicz U (2005) Arctic-North Atlantic interactions and multidecadal variability of the meridional overturning circulation. J Clim 18(19):4013–4031

[CR197] Kang SM, Xie SP, Deser C, Xiang B (2021) Zonal mean and shift modes of historical climate response to evolving aerosol distribution. Sci Bull 66(23):2405–2411 10.1016/j.scib.2021.07.013 36654126

[CR198] Kanzow T, Send U, Zenk W, Chave AD, Rhein M (2006) Monitoring the integrated deep meridional flow in the tropical North Atlantic: long-term performance of a geostrophic array. Deep Sea Res Part I 53(3):528–546

[CR199] Karnauskas KB, Zhang L, Amaya DJ (2021) The atmospheric response to North Atlantic SST trends, 1870–2019. Geophys Res Lett 48:e2020GL090677

[CR200] Karspeck AR, Stammer D, Köhl A et al (2017) Comparison of the Atlantic meridional overturning circulation between 1960 and 2007 in six ocean reanalysis products. Clim Dyn 49:957–982. 10.1007/s00382-015-2787-7

[CR201] Keeley S, Sutton R, Shaffrey L (2012) The impact of North Atlantic sea surface temperature errors on the simulation of North Atlantic European region climate. Q J R Meteorol Soc 138(668):1774–1783

[CR202] Keller DP, Lenton A, Scott V et al (2018) The carbon dioxide removal model intercomparison project (CDRMIP): rationale and experimental protocol for CMIP6. Geosci Model Dev 11:1133–1160

[CR203] Kelly SJ, Proshutinsky A, Popova EK, Aksenov YK, Yool A (2019) On the origin of water masses in the Beaufort Gyre. J Geophys Res Oceans 124:4696–4709. 10.1029/2019JC015022

[CR204] Kerr RA (2000) A North Atlantic climate pacemaker for the centuries. Science 288(5473):1984–1985 17835110 10.1126/science.288.5473.1984

[CR205] Kidston J, Gerber EP (2010) Intermodel variability of the poleward shift of the austral jet stream in the CMIP3 integrations linked to biases in 20th century climatology. Geophys Res Lett. 10.1029/2010GL042873

[CR207] Klavans JM, Cane MA, Clement AC, Murphy LN (2021) NAO predictability from external forcing in the late 20th century. NPJ Clim Atmos Sci 4(1):22

[CR208] Knight JR, Folland CK, Scaife AA (2006) Climate impacts of the Atlantic multidecadal oscillation. Geophys Res Lett 33:L17706. 10.1029/2006GL026242

[CR209] Kornhuber K, Petoukhov V, Karoly D, Petri S, Rahmstorf S, Coumou D (2017) Summertime planetary wave resonance in the Northern and Southern Hemispheres. J Clim 30(16):6133–6150

[CR210] Kornhuber K, Osprey S, Coumou D, Petri S, Petoukhov V, Rahmstorf S, Gray L (2019) Extreme weather events in early summer 2018 connected by a recurrent hemispheric wave-7 pattern. Environ Res Lett 14(5):054002

[CR211] Kucharski F, Ikram F, Molteni F et al (2016) Atlantic forcing of Pacific decadal variability. Clim Dyn 46(7–8):2337–2351. 10.1007/s00382-015-2705-z

[CR212] Kuroda Y, Kodera K, Yoshida K, Yukimoto S, Gray L (2022) Influence of the solar cycle on the North Atlantic Oscillation. J Geophys Res Atmos 127:e2021JD035519. 10.1029/2021JD035519

[CR213] Kushnir Y (1994) Interdecadal variations in North Atlantic sea surface temperature and associated atmospheric conditions. J Clim 7(1):141–157

[CR214] Kushnir Y, Robinson WA, Bladé I et al (2002) Atmospheric GCM response to extratropical SST anomalies: synthesis and evaluation. J Clim 15(16):2233–2256

[CR215] Lai WKM, Robson JI, Wilcox LJ, Dunstone N (2022) Mechanisms of internal Atlantic multidecadal variability in HadGEM3-GC3. 1 at two different resolutions. J Clim 35(4):1365–1383

[CR216] Lamarque J-F et al (2010) Historical (1850–2000) gridded anthropogenic and biomass burning emissions of reactive gases and aerosols: methodology and application. Atmos Chem Phys 10:7017–7039. 10.5194/acp-10-7017-2010

[CR217] Lazier JR (1980) Oceanographic conditions at ocean weather ship Bravo, 1964–1974. Atmos Ocean 18(3):227–238

[CR218] Lee JY, Marotzke J, Bala G et al (2021) Future Global Climate: Scenario-Based Projections and Near-Term Information. In: Masson-Delmotte V, Zhai P, Pirani A, Connors SL, Péan C, Berger S et al (eds) In climate change 2021: the physical science basis. Contribution of Working Group I to the Sixth Assessment Report of the Intergovernmental Panel on Climate Change. Cambridge, UK and New York, NY, USA: Cambridge University Press, pp 553–672. Available from: 10.1017/9781009157896.006

[CR219] Lee RW, Woollings TJ, Hoskins BJ et al (2018) Impact of Gulf Stream SST biases on the global atmospheric circulation. Clim Dyn 51:3369–3387. 10.1007/s00382-018-4083-9

[CR220] Lehner F, Hawkins E, Sutton R, Pendergrass AG, Moore FC (2023) New potential to reduce uncertainty in regional climate projections by combining physical and socio-economic constraints. AGU Adv 4:e2023AV000887. 10.1029/2023AV000887

[CR221] Levang SJ, Schmitt RW (2020) What causes the AMOC to weaken in CMIP5? J Clim 33(4):1535–1545

[CR222] Levermann A, Griesel A, Hofmann M, Montoya M, Rahmstorf S (2005) Dynamic sea level changes following changes in the thermohaline circulation. Clim Dyn 24:347–354

[CR223] Lin P, Pickart RS, Heorton H et al (2023) Recent state transition of the Arctic Ocean’s Beaufort Gyre. Nat Geosci. 10.1038/s41561-023-01184-5

[CR224] Little CM, Hu A, Hughes CW et al (2019) The relationship between U.S. east coast sea level and the Atlantic meridional overturning circulation: a review. J Geophys Res Ocean 124:6435–6458 10.1029/2019JC015152 PMC6853260 31763114

[CR225] Liu F, Li X, Luo Y et al (2024) Increased Asian aerosols drive a slowdown of Atlantic Meridional overturning circulation. Nat Commun 15:18. 10.1038/s41467-023-44597-x 38168125 10.1038/s41467-023-44597-x PMC10762259

[CR226] Liu G, Wang P, Beveridge M, Kwon YO, Drori I (2021) Predicting Atlantic multidecadal variability. arXiv preprint arXiv:2111.00124.

[CR227] Liu G, Wang P, Kwon YO (2023) Physical insights from the multidecadal prediction of North Atlantic sea surface temperature variability using explainable neural networks. Geophys Res Lett 50(24):e2023GL106278

[CR228] Liu W, Xie SP, Liu Z, Zhu J (2017) Overlooked possibility of a collapsed Atlantic meridional overturning circulation in warming climate. Sci Adv 3(1):e1601666 28070560 10.1126/sciadv.1601666 PMC5217057

[CR229] Liu W, Fedorov A, Sévellec F (2019) The mechanisms of the Atlantic meridional overturning circulation slowdown induced by Arctic sea ice decline. J Clim 32(4):977–996

[CR230] Lobelle D, Beaulieu C, Livina V, Sevellec F, Frajka-Williams E (2020) Detectability of an AMOC decline in current and projected climate changes. Geophys Res Lett 47(20):e2020GL089974

[CR231] Lockwood M, Harrison RG, Woollings T, Solanki SK (2010) Are cold winters in Europe associated with low solar activity? Environ Res Lett 5(2):024001

[CR232] Lorenz DJ, DeWeaver ET (2007) Tropopause height and zonal wind response to global warming in the IPCC scenario integrations. J Geophys Res: Atmos 112(D10)

[CR233] Lorenz DJ, Hartmann DL (2003) Eddy–zonal fow feedback in the Northern Hemisphere winter. J Clim 16:1212–1227

[CR234] Loriani S, Aksenov Y, Dijkstra H et al (2023a) Global tipping points 2023 Report: Ch1.4—Tipping points in ocean and atmosphere circulations. In: Lenton TM, Armstrong McKay DI, Loriani S, Abrams JF, Lade SJ, Donges JF, Milkoreit M, Powell T, Zimm C, Smith SR, Buxton JE, Laybourn L, Ghadiali A, Dyke J (eds) The global tipping points report 2023a. University of Exeter, Exeter

[CR235] Loriani S, Aksenov Y, Armstrong McKay D et al (2023b) Tipping points in ocean and atmosphere circulations. EGUsphere. 10.5194/egusphere-2023-2589

[CR236] Lozier MS, Leadbetter S, Williams RG et al (2008) The spatial pattern and mechanisms of heat-content change in the North Atlantic. Science 319(5864):800–803 18174399 10.1126/science.1146436

[CR237] Lozier MS, Roussenov V, Reed MS, Williams RG (2010) Opposing decadal changes for the North Atlantic meridional overturning circulation. Nat Geosci 3(10):728–734. 10.1038/ngeo947

[CR238] Lozier MS, Li F, Bacon S et al (2019) A sea change in our view of overturning in the subpolar North Atlantic. Science 363(6426):516–521 30705189 10.1126/science.aau6592

[CR239] Lu H, Gray LJ, Baldwin MP, Jarvis MJ (2009) Life cycle of the QBO-modulated 11-year solar cycle signals in the Northern Hemispheric winter. Q J R Meteorol Soc 135:1030–1043. 10.1002/qj.419

[CR240] Lu H, Scaife AA, Marshall GL, Turner J, Gray LJ (2017a) Downward wave reflection as a mechanism for the stratosphere–troposphere response to the 11-yr solar cycle. J Clim 30:2395–2414. 10.1175/JCLI-D-16-0400.1

[CR241] Lu H, Gray LJ, White IP, Bracegirdle TJ (2017b) Stratospheric response to the 11-Yr solar cycle: breaking planetary waves, internal reflection, and resonance. J Climate 30:7169–7190. 10.1175/JCLI-D-17-0023.1

[CR242] Lu J, Chen G, Frierson DM (2008) Response of the zonal mean atmospheric circulation to El Niño versus global warming. J Clim 21(22):5835–5851

[CR243] Ma L, Woollings T, Williams RG, Smith D, Dunstone N (2020) How does the winter jet stream affect surface temperature, heat flux, and sea ice in the North Atlantic? J Clim 33(9):3711–3730

[CR244] Magnusdottir G, Deser C, Saravanan R (2004) The effects of North Atlantic SST and sea ice anomalies on the winter circulation in CCM3. Part I: main features and storm track characteristics of the response. J Climate 17:857–876

[CR245] Manabe S, Stouffer RJ (1999) The role of thermohaline circulation in climate. Tellus B 51(1):91–109

[CR246] Mann ME, Rahmstorf S, Kornhuber K, Steinman BA, Miller SK, Petri S, Coumou D (2018) Projected changes in persistent extreme summer weather events: the role of quasi-resonant amplification. Sci Adv. 10.1126/sciadv.aat3272 10.1126/sciadv.aat3272 PMC6209391 30402537

[CR247] Manzini E, Karpechko AY, Anstey J et al (2014) Northern winter climate change: assessment of uncertainty in CMIP5 projections related to stratosphere-troposphere coupling. J Geophys Res 119:7979–7998. 10.1002/2013JD021403

[CR248] Marcheggiani A, Robson J, Monerie PA, Bracegirdle TJ, Smith D (2023) Decadal predictability of the North Atlantic eddy-driven jet in winter. Geophys Res Lett 50:e2022GL102071. 10.1029/2022GL102071

[CR249] Marshall J, Johnson H, Goodman J (2001) A study of the interaction of the North Atlantic Oscillation with ocean circulation. J Clim 14:1399–1421

[CR250] Marshall AG, Scaife AA, Ineson S (2009) Enhanced seasonal prediction of European winter warming following volcanic eruptions. J Clim 22(23):6168–6180

[CR251] Marshall LR, Maters EC, Schmidt A et al (2022) Volcanic effects on climate: recent advances and future avenues Bull. Volcanol 84:54

[CR252] Mayer KJ, Barnes EA (2021) Subseasonal forecasts of opportunity identified by an explainable neural network. Geophys Res Lett 48(10):e2020GL092092

[CR253] Mbengue C, Schneider T (2017) Storm-track shifts under climate change: toward a mechanistic understanding using baroclinic mean available potential energy. J Atmos Sci 74(1):93–110

[CR254] McCarthy GD, Caesar L (2023) Can we trust projections of AMOC weakening based on climate models that cannot reproduce the past? Phil Trans R Soc A 381:20220193. 10.1098/rsta.2022.0193 37866378 10.1098/rsta.2022.0193 PMC10590661

[CR255] McGovern A, Lagerquist R, Gagne DJ et al (2019) Making the black box more transparent: understanding the physical implications of machine learning. Bull Am Meteor Soc 100(11):2175–2199

[CR256] McKenna CM, Maycock AC (2021) Sources of uncertainty in multimodel large ensemble projections of the winter North Atlantic Oscillation. Geophys Res Lett 48(14):e2021GL093258

[CR257] Meccia VL, Fuentes-Franco R, Davini P et al (2023) Internal multi-centennial variability of the Atlantic Meridional overturning circulation simulated by EC-Earth3. Clim Dyn 60:3695–3712. 10.1007/s00382-022-06534-4

[CR258] Mecking JV, Drijfhout SS, Jackson LC, Graham T (2016) Stable AMOC off state in an eddy-permitting coupled climate model. Clim Dyn 47:2455–2470

[CR259] Mecking JV, Drijfhout SS, Jackson LC, Andrews MB (2017) The effect of model bias on Atlantic freshwater transport and implications for AMOC bi-stability. Tellus A: Dyn Meteorol Oceanogr 69(1):1299910

[CR260] Mecking JV, Drijfhout SS (2023) The decrease in ocean heat transport in response to global warming. Nat Clim Chang 13(11):1229–1236 10.1038/s41558-023-01829-8 PMC13538074 42694091

[CR261] Meehl GA, Stocker TF, Collins WD et al (2007) Global climate projections. In: Solomon S, Qin D, Manning M, Chen Z, Marquis M, Averyt KB, Tignor M, Miller HL (eds.) Climate change 2007: The Physical Science Basis. Contribution of Working Group I to the Fourth Assessment Report of the Intergovernmental Panel on Climate Change. Cambridge University Press, Cambridge, United Kingdom and New York

[CR262] Megann A, Blaker A, Josey S, New A, Sinha B (2021) Mechanisms for late 20th and early 21st Century decadal AMOC variability. J Geophys Res. 10.1029/2021JC017865

[CR263] Menary MB, Hermanson L (2018) Limits on determining the skill of North Atlantic Ocean decadal predictions. Nat Commun 9:1694. 10.1038/s41467-018-04043-9 29703895 10.1038/s41467-018-04043-9 PMC5923258

[CR264] Menary MB, Scaife AA (2014) Naturally forced multidecadal variability of the Atlantic meridional overturning circulation. Clim Dyn 42:1347–1362

[CR265] Menary MB, Hodson DL, Robson JI et al (2015) Exploring the impact of CMIP5 model biases on the simulation of North Atlantic decadal variability. Geophys Res Lett 42(14):5926–5934

[CR266] Menary MB, Hermanson L, Dunstone NJ (2016) The impact of Labrador Sea temperature and salinity variability on density and the subpolar AMOC in a decadal prediction system. Geophys Res Lett 43(23):12–217

[CR267] Menary MB, Robson J, Allan RP et al (2020) Aerosol-forced AMOC changes in CMIP6 historical simulations. Geophys Res Lett. 10.1029/2020GL088166

[CR268] Meneghello G, Marshall J, Campin JM, Doddridge E, Timmermans ML (2018) The ice-ocean governor: Ice-ocean stress feedback limits Beaufort Gyre spin-up. Geophys Res Lett 45(20):11–293

[CR269] Mercer AE (2021) Dominant United States Cold-Season near surface temperature anomaly patterns derived from kernel methods. Int J Climatol 41(4):2383–2396

[CR270] Mignot J, Khodri M, Frankignoul C, Servonnat J (2011) Volcanic impact on the Atlantic Ocean over the last millennium. Clim past 7(4):1439–1455. 10.5194/cp-7-1439-2011

[CR271] Moat BI, Sinha B, Berry DI et al (2024) Ocean Heat Convergence and North Atlantic multidecadal heat content variability. J Clim

[CR272] Monerie PA, Robson J, Dong BW et al (2018) (2018) A role of the Atlantic Ocean in predicting summer surface air temperature over North East Asia? Clim Dyn 51:473–491. 10.1007/s00382-017-3935-z

[CR273] Moore FC, Lacasse K, Mach KJ, Beckage B (2022) Determinants of emissions pathways in the coupled climate–social system. Nature 603(7899):103–111. 10.1038/s41586-022-04423-8 35173331 10.1038/s41586-022-04423-8

[CR274] Moreno-Chamarro E, Caron LP, Loosveldt Tomas S et al (2022) Impact of increased resolution on long-standing biases in HighResMIP-PRIMAVERA climate models. Geosci Model Dev 15(1):269–289

[CR275] Msadek R, Frankignoul C, Li LZ (2011) Mechanisms of the atmospheric response to North Atlantic multidecadal variability: a model study. Clim Dyn 36:1255–1276

[CR276] Msadek R, Delworth TL, Rosati A et al (2014) Predicting a decadal shift in North Atlantic climate variability using the GFDL forecast system. J Clim 27(17):6472–6496

[CR277] Muir LC, Fedorov AV (2017) Evidence of the AMOC interdecadal mode related to westward propagation of temperature anomalies in CMIP5 models. Clim Dyn 48:1517–1535. 10.1007/s00382-016-3157-9

[CR278] Myhre G et al (2013) Radiative forcing of the direct aerosol effect from AeroCom Phase II simulations. Atmos Chem Phys 13:1853–1877. 10.5194/acp-13-1853-2013

[CR279] Needham MR, Falter DD, Randall DA (2024) Changes in external forcings drive divergent AMOC responses across CESM generations. Geophys Res Lett 51(5):e2023GL106410

[CR280] Nishino S, Jung J, Cho KH et al (2023) Atlantic-origin water extension into the Pacific Arctic induced an anomalous biogeochemical event. Nat Commun 14:6235. 10.1038/s41467-023-41960-w 37919271 10.1038/s41467-023-41960-w PMC10622542

[CR281] Nguyen H, Lucas C, Evans A, Timbal B, Hanson L (2015) Expansion of the Southern Hemisphere Hadley cell in response to greenhouse gas forcing. J Clim 28(20):8067–8077

[CR282] Oltmanns M, Karstensen J, Moore GWK, Josey SA (2020) Rapid cooling and increased storminess triggered by freshwater in the North Atlantic. Geophys Res Lett. 10.1029/2020GL087207

[CR283] Oltmanns M, Holliday NP, Screen J et al (2024) European summer weather linked to North Atlantic freshwater anomalies in preceding years, Weather and Climate Dynamics, accepted subject to minor revisions.

[CR284] Omrani NE, Keenlyside NS, Bader J, Manzini E (2014) Stratosphere key for wintertime atmospheric response to warm Atlantic decadal conditions. Clim Dyn 42(3–4):649–663. 10.1007/s00382-013-1860-3

[CR285] Omrani N-E, Keenlyside N, Matthes K, Boljka L, Zanchettin D, Jungclaus JH, Lubis SW (2022) Coupled stratosphere-troposphere-Atlantic multidecadal oscillation and its importance for near-future climate projection. Npj Clim Atmos Sci 5:59. 10.1038/s41612-022-00275-1

[CR286] O’Reilly C, Woollings T, Zanna L, Weisheimer A (2019) An interdecadal shift of the extratropical ENSO teleconnection during boreal summer. Geophys Res Lett. 10.1029/2019GL084079

[CR287] O’Reilly CH, Befort DJ, Weisheimer A et al (2021) Projections of northern hemisphere extratropical climate underestimate internal variability and associated uncertainty. Commun Earth Environ. 10.1038/s43247-021-00268-7

[CR288] Ortega P, Lehner F, Swingedouw D et al (2015) A model-tested North Atlantic Oscillation reconstruction for the past millennium. Nature 523(7558):71–74. 10.1038/nature14518 26135450 10.1038/nature14518

[CR289] Ortega P, Robson JI, Menary M et al (2021) Labrador Sea subsurface density as a precursor of multidecadal variability in the North Atlantic: a multi-model study. Earth Syst Dyn 12(2):419–438

[CR290] Osborn TJ (2004) Simulating the winter North Atlantic Oscillation: the roles of internal variability and greenhouse gas forcing. Clim Dyn. 10.1007/s00382-004-0405-1

[CR291] Osman MB, Coats S, Das SB, McConnell JR, Chellman N (2021) North Atlantic jet stream projections in the context of the past 1250 years. Proc Natl Acad Sci 118(38):e2104105118 34518222 10.1073/pnas.2104105118 PMC8463874

[CR292] Osso A, Sutton R, Shaffrey L, Dong B (2020) Development, amplification, and decay of Atlantic/European summer weather patterns linked to spring North Atlantic sea surface temperatures. J Clim 33(14):5939–5951

[CR294] Otto IM, Donges JF, Cremades R et al (2020) Social tipping dynamics for stabilizing Earth’s climate by 2050. Proc Natl Acad Sci USA 117(5):2354–2365. 10.1073/pnas.1900577117 31964839 10.1073/pnas.1900577117 PMC7007533

[CR295] Oudar T, Cattiaux J, Douville H (2020) Drivers of the northern extratropical eddy-driven jet change in CMIP5 and CMIP6 models. Geophys Res Lett 47:e2019GL086695. 10.1029/2019GL086695

[CR296] Overland JE, Francis JA, Hall R, Hanna E, Kim S-J, Vihma T (2015) The melting Arctic and mid-latitude weather patterns: are they connected? J Clim 28(20):7917–7932. 10.1175/JCLI-D-14-00822.1

[CR297] Overland JE, Ballinger TJ, Cohen J, Francis JA, Hanna E, Jaiser R et al (2021) How do intermittency and simultaneous processes obfuscate the Arctic influence on midlatitude winter extreme weather events? Environ Res Lett 16(4):043002

[CR298] Paik S, Min SK, Son SW et al (2023) Impact of volcanic eruptions on extratropical atmospheric circulations: review, revisit and future directions. Environ Res Lett 18(6):063003

[CR299] Parfitt R, Czaja A, Kwon YO (2017) The impact of SST resolution change in the ERA-Interim reanalysis on wintertime Gulf Stream frontal air-sea interaction. Geophys Res Lett 44(7):3246–3254

[CR300] Park W, Latif M (2008) Multidecadal and multicentennial variability of the meridional overturning circulation. Geophys Res Lett 35:L22703. 10.1029/2008GL035779

[CR301] Pasini A, Amendola S, Federbusch E (2022) Is natural variability really natural? The case of Atlantic Multidecadal Oscillation investigated by a neural network model. Theoret Appl Climatol 150(1):881–892

[CR302] Pathak J, Subramanian S, Harrington P et al (2022) Fourcastnet: A global data-driven high-resolution weather model using adaptive Fourier neural operators. arXiv preprint arXiv:2202.11214

[CR303] Pausata FSR, Gaetani M, Messori G, Kloster S, Dentener FJ (2015a) The role of aerosol in altering North Atlantic atmospheric circulation in winter and its impact on air quality. Atmos Chem Phys 15(4):1725–1743

[CR304] Pausata FSR, Chafik L, Caballero R, Battisti DS (2015b) Impacts of high-latitude volcanic eruptions on ENSO and AMOC. Proc Natl Acad Sci 112(45):13784–13788. 10.1073/pnas.1509153112 26504201 10.1073/pnas.1509153112 PMC4653171

[CR305] Peings Y, Simpkins G, Magnusdottir G (2016) Multidecadal fluctuations of the North Atlantic Ocean and feedback on the winter climate in CMIP5 control simulations. J Geophys Res Atmos 121:2571–2592. 10.1002/2015JD024107

[CR306] Peings Y, Cattiaux J, Vavrus SJ, Magnusdottir G (2018) Projected squeezing of the wintertime North-Atlantic jet. Environ Res Lett 13:074016

[CR307] Piecuch CG, Dangendorf S, Gawarkiewicz GG et al (2019) How is New England coastal sea level related to the Atlantic meridional overturning circulation at 26° N? Geophys Res Lett 46(10):5351–5360

[CR308] Petrie RE, Shaffrey LC, Sutton RT (2015) Atmospheric response in summer linked to recent Arctic sea ice loss. Q J R Meteorol Soc 141(691):2070–2076. 10.1002/qj.2502

[CR309] Polo I, Robson J, Sutton R, Balmaseda MA (2014) The importance of wind and buoyancy forcing for the boundary density variations and the geostrophic component of the AMOC at 26 N. J Phys Oceanogr 44(9):2387–2408

[CR310] Polvani LM, Camargo SJ (2020) Scant evidence for a volcanically forced winter warming over Eurasia following the Krakatau eruption of August 1883. Atmos Chem Phys 20:13687–13700

[CR311] Polyakov IV, Pnyushkov AV, Alkire MB et al (2017) Greater role for Atlantic inflows on sea-ice loss in the Eurasian Basin of the Arctic Ocean. Science 356:285–291. 10.1126/science.aai8204 28386025 10.1126/science.aai8204

[CR312] Polyakov IV, Ingvaldsen RB, Pnyushkov AV et al (2023) Fluctuating Atlantic inflows modulate Arctic atlantification. Science 381:972–979. 10.1126/science.adh5158 37651524 10.1126/science.adh5158

[CR313] Proshutinsky AY, Johnson MA (1997) Two circulation regimes of the wind-driven Arctic Ocean. J Geophys Res: Oceans 102(C6):12493–12514. 10.1029/97JC00738

[CR314] Proshutinsky A, Dukhovskoy D, Timmermans ML, Krishfield R, Bamber JL (2015) Arctic circulation regimes. Philos Trans R Soc A: Math Phys Eng Sci 373(2052):20140160. 10.1098/rsta.2014.0160 10.1098/rsta.2014.0160 PMC4607701 26347536

[CR315] Proshutinsky A, Krishfield R, Toole JM et al (2019) Analysis of the Beaufort Gyre Freshwater content in 2003–2018. J Geophys Res: Oceans. 10.1029/2019JC015281 10.1029/2019JC015281 PMC7003849 32055432

[CR316] Qasmi S, Cassou C, Boé J (2020) Teleconnection processes linking the intensity of the Atlantic multidecadal variability to the climate impacts over Europe in boreal winter. J Clim 33(7):2681–2700

[CR317] Qasmi S, Sanchez-Gomez E, Ruprich-Robert Y, Boé J, Cassou C (2021) Modulation of the occurrence of heatwaves over the Euro-Mediterranean region by the intensity of the Atlantic multidecadal variability. J Clim 34:1099–1114

[CR319] Rahmstorf S, Crucifix M, Ganopolski A et al (2005) Thermohaline circulation hysteresis: a model intercomparison. Geophys Res Lett 32(23)

[CR320] Rantanen M, Karpechko AY, Lipponen A et al (2022) The Arctic has warmed nearly four times faster than the globe since 1979. Commun Earth Environ 3(1):168. 10.1038/s43247-022-00498-3

[CR321] Reintges A, Martin T, Latif M, Keenlyside NS (2017) Uncertainty in twenty-first century projections of the Atlantic Meridional Overturning Circulation in CMIP3 and CMIP5 models. Clim Dyn 49:1495–1511

[CR322] Reintges A, Robson I, Sutton R, Yeager SG (2024) Subpolar North Atlantic mean state affects the response of the Atlantic Meridional Overturning Circulation to the North Atlantic Oscillation in CMIP6 models. J Clim.

[CR323] Regan HC, Lique C, Armitage TWK (2019) The Beaufort Gyre extent, shape, and location between 2003 and 2014 from satellite observations. J Geophys Res: Oceans 124:844–862. 10.1029/2018JC014379

[CR324] Riboldi J, Lott F, d’Andrea F, Rivière G (2020) On the linkage between Rossby wave phase speed, atmospheric blocking, and Arctic amplification. Geophys Res Lett 47(19):e2020GL087796

[CR325] Riebold J, Richling A, Ulbrich U et al (2023) On the linkage between future Arctic sea ice retreat, Euro-Atlantic circulation regimes and temperature extremes over Europe. Weather Clim Dyn 4(3):663–682

[CR326] Robock A, Mao J (1995) The volcanic signal in surface temperature observations. J Clim 8(5):1086–1103

[CR327] Robock A (2000) Volcanic eruptions and climate. Rev Geophys 38(2):191–219

[CR328] Robson J, Sutton RT, Lohmann K, Smith D, Palmer MD (2012) Causes of the rapid warming of the North Atlantic ocean in the mid-1990s. J Clim 25(12):4116–4134. 10.1175/JCLI-D-11-00443.1

[CR329] Robson J, Hodson D, Hawkins ED, Sutton RT (2014) Atlantic overturning in decline? Nat Geosci 7(1):2–3

[CR330] Robson J, Ortega P, Sutton RT (2016) A reversal of climatic trends in the North Atlantic since 2005. Nature Geosci 9(7):513–517. 10.1038/ngeo2727

[CR331] Robson J, Sutton RT, Archibald A et al (2018) Recent multivariate changes in the North Atlantic climate system, with a focus on 2005–2016. Int J Climatol 38(14):5050–5076

[CR332] Robson J, Menary MB, Sutton RT et al (2022) The role of anthropogenic aerosol forcing in the 1850–1985 strengthening of the AMOC in CMIP6 historical simulations. J Clim 35(20):6843–6863

[CR333] Robson J, Sutton RT, Menary MB, Lai MWK (2023) Contrasting internally and externally generated Atlantic Multidecadal Variability and the role for AMOC in CMIP6 historical simulations. Phil Trans R Soc A 381:20220194. 10.1098/rsta.2022.0194 37866382 10.1098/rsta.2022.0194 PMC10590668

[CR334] Rotstayn LD, Collier MA, Jeffrey SJ et al (2013) Anthropogenic effects on the subtropical jet in the Southern Hemisphere: aerosols versus long-lived greenhouse gases. Environ Res Lett 8(1):014030

[CR335] Rousi E, Selten F, Rahmstorf S, Coumou D (2021) Changes in North Atlantic atmospheric circulation in a warmer climate favor winter flooding and summer drought over Europe. J Clim 34(6):2277–2295

[CR336] Rousi E, Kornhuber K, Beobide-Arsuaga G, Luo F, Coumou D (2022) Accelerated western European heatwave trends linked to more-persistent double jets over Eurasia. Nat Commun 13(1):3851 35788585 10.1038/s41467-022-31432-y PMC9253148

[CR337] Ruggieri P, Bellucci A, Nicolí D et al (2021) Atlantic multidecadal variability and North Atlantic jet: a multimodel view from the decadal climate prediction project. J Clim 34(1):347–360

[CR338] Santer BD, Thorne PW, Haimberger L et al (2008) Consistency of modelled and observed temperature trends in the tropical troposphere. Int J Climatol 28(13):1703–1722

[CR339] Scaife AA, Copsey D, Gordon C et al (2011) Improved Atlantic winter blocking in a climate model. Geophys Res Lett 38:L23703. 10.1029/2011GL049573

[CR340] Scaife AA, Ineson S, Knight JR et al (2013) A mechanism for lagged North Atlantic climate response to solar variability. Geophys Res Lett 40(2):434–439. 10.1002/grl.50099

[CR342] Scaife AA, Smith D (2018) A signal-to-noise paradox in climate science. npj Clim Atmos Sci 1:28. 10.1038/s41612-018-0038-4

[CR343] Scaife AA, Camp J, Comer R et al (2019) Does increased atmospheric resolution improve seasonal climate predictions? Atmos Sci Lett 20(8):e922

[CR344] Schurer AP, Hegerl GC, Goosse H et al (2023) Role of multi-decadal variability of the winter North Atlantic Oscillation on Northern Hemisphere climate. Environ Res Lett 18(4):044046

[CR345] Schwinger J, Asaadi A, Goris N, Lee H (2022) Possibility for strong northern hemisphere high-latitude cooling under negative emissions. Nature Commun 13:1095. 10.1038/s41467-022-28573-5 35232955 10.1038/s41467-022-28573-5 PMC8888562

[CR347] Screen JA (2017) Simulated atmospheric response to regional and pan-Arctic sea ice loss. J Clim 30:3945–3962. 10.1175/JCLI-D-16-0197.1

[CR348] Screen JA, Simmonds I (2010) The central role of diminishing sea ice in recent Arctic temperature amplification. Nature 464(7293):1334–1337 20428168 10.1038/nature09051

[CR349] Screen JA, Simmonds I, Deser C, Tomas R (2013) The atmospheric response to three decades of observed Arctic sea ice loss. J Clim 26:1230–1248. 10.1175/jcli-d-12-00063.1

[CR350] Screen JA, Deser C, Smith DM, Zhang X, Blackport R, Kushner PJ, Oudar T, McCusker KE, Sun L (2018) Consistency and discrepancy in the atmospheric response to Arctic sea-ice loss across climate models. Nat Geosci 11:155–163. 10.1038/s41561-018-0059-y

[CR351] Screen JA, Eade R, Smith DM, Thomson S, Yu H (2022) Net equatorward shift of the jet streams when the contribution from sea-ice loss is constrained by observed Eddy feedback. Geophys Res Lett 49(23):e2022GL100523

[CR352] Sen Gupta A, Stellema A, Pontes GM et al (2021) Future changes to the upper ocean Western Boundary currents across two generations of climate models. Sci Rep 11(1):9538 33953259 10.1038/s41598-021-88934-w PMC8099859

[CR353] Seo H, O’Neill LW, Bourassa MA et al (2023) Ocean mesoscale and frontal-scale ocean–atmosphere interactions and influence on large-scale climate: a review. J Clim 36(7):1981–2013

[CR354] Sévellec F, Fedorov AV (2013) The leading, interdecadal eigenmode of the Atlantic meridional overturning circulation in a realistic ocean model. J Clim 26(7):2160–2183

[CR355] Sévellec F, Drijfhout SS (2019) The signal-to-noise paradox for interannual surface atmospheric temperature predictions. Geophys Res Lett 46(15):9031–9041

[CR356] Sévellec F, Fedorov AV, Liu W (2017) Arctic sea-ice decline weakens the Atlantic Meridional overturning circulation. Nat Clim Change 7:604–610

[CR357] Serreze MC, Barrett AP, Slater AG et al (2006) The large-scale freshwater cycle of the Arctic. J Geophys Res: Oceans. 10.1029/2005JC003424

[CR358] Serreze MC, Barrett AP, Stroeve JC, Kindig DN, Holland MM (2009) The emergence of surface-based Arctic amplification. Cryosphere 3:11–19. 10.5194/tc-3-11-2009

[CR359] Shaw TA, Baldwin M, Barnes E et al (2016) Storm track processes and the opposing influences of climate change. Nature Geosci 9:656–664. 10.1038/ngeo2783

[CR360] Shaw TA (2019) Mechanisms of future predicted changes in the zonal mean Mid Latitude circulation. Curr Clim Change Rep 5:345–357. 10.1007/s40641-019-00145-8

[CR361] Shaw TA, Arias PA, Collins M et al (2024) Regional climate change: consensus, discrepancies, and ways forward. Front Clim 6:1391634

[CR362] Shen Z, Ming Y (2018) The influence of aerosol absorption on the extratropical circulation. J Clim 31(15):5961–5975

[CR363] Shine KP, Bourqui MS, Forster PDF et al (2003) A comparison of model-simulated trends in stratospheric temperatures. Q J R Meteorol Soc 129(590):1565–1588

[CR364] Sgubin G, Swingedouw D, Drijfhout S, Mary Y, Bennabi A (2017) Abrupt cooling over the North Atlantic in modern climate models. Nat Commun 8(1):14375 28198383 10.1038/ncomms14375 PMC5330854

[CR365] Simmons AJ (2022) Trends in the tropospheric general circulation from 1979 to 2022. Weather Clim Dyn 3(3):777–809

[CR366] Simpson IR, Polvani LM (2016) Revisiting the relationship between jet position, forced response, and annular mode variability in the southern midlatitudes. Geophys Res Lett 43(6):2896–2903

[CR367] Simpson IR, Shaw TA, Seager R (2014) A diagnosis of the seasonally and longitudinally varying midlatitude circulation response to global warming. J Atmos Sci 71:2489–2515. 10.1175/JAS-D-13-0325.1

[CR368] Simpson IR, Deser C, McKinnon KA, Barnes EA (2018) Modeled and observed multidecadal variability in the North Atlantic jet stream and its connection to sea surface temperatures. J Climate 31:8313–8338. 10.1175/JCLID-18-0168.1

[CR369] Simpson IR, Yeager SG, McKinnon KA, Deser C (2019) Decadal predictability of late winter precipitation in western Europe through an ocean–jet stream connection. Nat Geosci 12:613–619. 10.1038/s41561-019-0391-x

[CR370] Simpson I, Hanna E, Baker L, Sun Y, Wei HL (2024) North Atlantic atmospheric circulation indices: links with summer and winter temperature and precipitation in north-west Europe, including persistence and variability. Int J Climatol 44(3):902–922

[CR371] Siqueira L, Kirtman BP (2016) Atlantic near-term climate variability and the role of a resolved Gulf Stream. Geophys Res Lett 43(8):3964–3972

[CR372] Sjolte J, Sturm C, Adolphi F et al (2018) Solar and volcanic forcing of North Atlantic climate inferred from a process-based reconstruction. Clim Past 14(8):1179–1194. 10.5194/cp-14-1179-2018

[CR373] Sjolte J, Adolphi F, Guðlaugsdòttir H, Muscheler R (2021) Major differences in regional climate impact between high- and low-latitude volcanic eruptions. Geophys Res Lett 48(8):e2020GL092017. 10.1029/2020GL092017

[CR374] Smedsrud LH, Muilwijk M, Brakstad A et al (2022) Nordic seas heat loss, atlantic inflow, and arctic sea ice cover over the last century. Rev Geophys 60:e2020RG000725. 10.1029/2020RG000725

[CR375] Smirnov D, Newman M, Alexander MA, Kwon YO, Frankignoul C (2015) Investigating the local atmospheric response to a realistic shift in the Oyashio sea surface temperature front. J Clim 28(3):1126–1147

[CR376] Smith DM, Dunstone NJ, Scaife AA, Fiedler EK, Copsey D, Hardiman SC (2017) Atmospheric response to Arctic and Antarctic sea ice: the importance of ocean-atmosphere coupling and the background state. J Clim 30:4547–4565

[CR377] Smith DM, Scaife AA, Eade R et al (2020) North Atlantic climate far more predictable than models imply. Nature 583(7818):796–800 32728237 10.1038/s41586-020-2525-0

[CR378] Smith DM, Eade R, Andrews MB et al (2022a) Robust but weak winter atmospheric circulation response to future Arctic sea ice loss. Nat Commun 13:727. 10.1038/s41467-022-28283-y 35132058 10.1038/s41467-022-28283-y PMC8821642

[CR379] Smith DM, Gillett NP, Simpson IR et al (2022b) Attribution of multi-annual to decadal changes in the climate system: The Large Ensemble Single Forcing Model Intercomparison Project (LESFMIP). Front Clim 4:955414

[CR380] Solomon A, Heuzé C, Rabe B et al (2021) Freshwater in the arctic ocean 2010–2019. Ocean Sci 17(4):1081–1102. 10.5194/os-2020-113

[CR381] Srokosz M, Danabasoglu G, Patterson M (2021) Atlantic meridional overturning circulation: reviews of observational and modeling advances—an introduction. J Geophys Res Oceans 126:e2020JC016745. 10.1029/2020JC016745

[CR382] Stenchikov G, Delworth TL, Ramaswamy V et al (2009) Volcanic signals in oceans. J Geophys Res: Atmos 114(D16)

[CR383] Stendel M, Francis J, White R, Williams PD, Woollings T (2021) The jet stream and climate change. In Climate change. Elsevier, pp 327–357

[CR384] Stouffer RJ, Yin J, Gregory JM et al (2006) Investigating the causes of the response of the thermohaline circulation to past and future climate changes. J Clim 19:1365–1387

[CR385] Stommel H (1961) Thermohaline convection with two stable regimes of flow. Tellus 13(2):224–230. 10.1111/j.2153-3490.1961.tb00079.x

[CR386] Strommen K, Palmer TN (2019) Signal and noise in regime systems: a hypothesis on the predictability of the north Atlantic oscillation. Q J R Meteorol Soc 145(718):147–163. 10.1002/qj.3414

[CR387] Strommen K, Woollings T, Davini P, Ruggieri P, Simpson IR (2023) Predictable decadal forcing of the North Atlantic jet speed by sub-polar North Atlantic sea surface temperatures. Weather Clim Dyn 4(4):853–874

[CR389] Sun Y, Simpson I, Wei HL, Hanna E (2014) Probabilistic seasonal forecasts of North Atlantic atmospheric circulation using complex systems modelling and comparison with dynamical models. Meteorol Appl 31:e2178

[CR388] Sun L, Alexander M, Deser C (2018) Evolution of the global coupled climate response to Arctic sea ice loss during 1990–2090 and its contribution to climate change. J Clim 31(19):7823–7843

[CR390] Sundby S, Drinkwater K (2007) On the mechanisms behind salinity anomaly signals of the northern North Atlantic. Prog Oceanogr 73(2):190–202

[CR391] Sutton RT, Dong BW (2012) Atlantic Ocean influence on a shift in European climate in the 1990s. Nat Geosci 5(11):788–792. 10.1038/ngeo1595

[CR392] Sutton RT, Hodson DL (2005) Atlantic Ocean forcing of North American and European summer climate. Science 309(5731):115–118. 10.1126/science.1109496 15994552 10.1126/science.1109496

[CR393] Sutton RT, McCarthy GD, Robson J et al (2017) Atlantic multi-decadal variability and the UK ACSIS programme. Bull Am Meteor Soc 99:415–425. 10.1175/BAMS-D-16-0266.1

[CR394] Swingedouw D, Mignot J, Ortega P et al (2017) Impact of explosive volcanic eruptions on the main climate variability modes Glob. Planet Change 150:24–45

[CR395] Swingedouw D, Ifejika Speranza C, Bartsch A et al (2020) Early warning from space for a few key tipping points in physical, biological, and social-ecological systems. Surv Geophys 41:1237–1284

[CR396] Swingedouw D, Bily A, Esquerdo C et al (2021) On the risk of abrupt changes in the North Atlantic subpolar gyre in CMIP6 models. Ann N Y Acad Sci 1504(1):187–201 34212391 10.1111/nyas.14659

[CR397] Szopa S, Naik V, Adhikary B, Artaxo P, Berntsen T, Collins WD, Fuzzi S, Gallardo L, Kiendler-Scharr A, Klimont Z, Liao H, Unger N, Zanis P (2021) Short-lived climate forcers. In: Masson-Delmotte V, Zhai P, Pirani A, Connors SL, Péan C, Berger S, Caud N, Chen Y, Goldfarb L, Gomis MI, Huang M, Leitzell K, Lonnoy E, Matthews JBR, Maycock TK, Waterfield T, Yelekçi O, Yu R, Zhou B (eds) Climate change 2021: the physical science basis. Contribution of Working Group I to the Sixth Assessment Report of the Intergovernmental Panel on Climate Change. Cambridge University Press, Cambridge, United Kingdom and New York, NY, USA, pp 817–922

[CR398] Tao L, Hu Y, Liu J (2016) Anthropogenic forcing on the Hadley circulation in CMIP5 simulations. Clim Dyn 46:3337–3350

[CR399] Teng H, Leung R, Branstator G, Lu J, Ding Q (2022) Warming pattern over the Northern Hemisphere midlatitudes in boreal summer 1979–2020. J Clim 35:3479–3494

[CR400] Tesi T, Muschitiello F, Mollenhauer G et al (2021) Rapid Atlantification along the Fram Strait at the beginning of the 20th century. Sci Adv 7(48):eabj2946 34818051 10.1126/sciadv.abj2946 PMC8612687

[CR401] Thornalley DJR, Oppo DW, Ortega P et al (2018) Anomalously weak Labrador Sea convection and Atlantic overturning during the past 150 years. Nature 556:227–230. 10.1038/s41586-018-0007-4 29643484 10.1038/s41586-018-0007-4

[CR402] Timmermann A, Okumura Y, An SI et al (2007) The influence of a weakening of the Atlantic meridional overturning circulation on ENSO. J Clim 20(19):4899–4919. 10.1175/JCLI4283

[CR403] Timmermans ML, Toole JM (2023) The Arctic Ocean’s Beaufort Gyre. Ann Rev Mar Sci 15:223–248. 10.1146/annurev-marine-032122-012034 10.1146/annurev-marine-032122-012034 35973719

[CR404] Thomson SI, Vallis GK (2018a) Atmospheric response to SST anomalies. Part I: background-state dependence, teleconnections, and local effects in winter. J Atmos Sci 75(12):4107–4124

[CR405] Thomson SI, Vallis GK (2018b) Atmospheric response to SST anomalies. Part II: background-state dependence, teleconnections, and local effects in summer. J Atmos Sci 75(12):4125–4138

[CR406] Tjiputra J, Schwinger J, Seland Ø et al (2023) NCC NorESM2-LM model output prepared for CMIP6 CDRMIP esm-ssp534-over. Version *YYYYMMDD*[1]. Earth Syst Grid Feder. 10.22033/ESGF/CMIP6.13747

[CR407] Toms BA, Barnes EA, Ebert-Uphoff I (2020) Physically interpretable neural networks for the geosciences: applications to earth system variability. J Adv Model Earth Syst 12(9):e2019MS002002

[CR408] Tsartsali EE, Haarsma RJ, Athanasiadis PJ et al (2022) Impact of resolution on the atmosphere–ocean coupling along the Gulf Stream in global high resolution models. Clim Dyn 58:3317–3333. 10.1007/s00382-021-06098-9

[CR409] Ulbrich U, Pinto JG, Kupfer H et al (2008) Changing Northern Hemisphere storm tracks in an ensemble of IPCC climate change simulations. J Clim 21(8):1669–1679

[CR410] Undorf S, Bollasina MA, Hegerl GC (2018a) Impacts of the 1900–74 increase in anthropogenic aerosol emissions from North America and Europe on Eurasian summer climate. J Clim 31(20):8381–8399

[CR411] Undorf S, Bollasina MA, Booth BBB, Hegerl GC (2018b) Contrasting the effects of the 1850–1975 increase in sulphate aerosols from North America and Europe on the Atlantic in the CESM. Geophys Res Lett 45(21):11–930

[CR412] van Westen RM, Kliphuis M, Dijkstra HA (2024) Physics-based early warning signal shows that AMOC is on tipping course. Sci Adv 10(6):eadk1189 38335283 10.1126/sciadv.adk1189 PMC10857529

[CR413] Valdes P (2011) Built for stability. Nat Geosci 4(7):414–416

[CR414] Vellinga M, Wood RA (2002) Global climatic impacts of a collapse of the Atlantic thermohaline circulation. Clim Change 54(3):251–267. 10.1023/A:1016168827653

[CR415] Vellinga M, Wu P (2004) Low-latitude freshwater influence on centennial variability of the Atlantic thermohaline circulation. J Clim 17(23):4498–4511

[CR416] Volkov DL, Baringer M, Smeed D, Johns W, Landerer FW (2019) Teleconnection between the Atlantic meridional overturning circulation and sea level in the Mediterranean Sea. J Clim 32(3):935–955

[CR417] Volkov DL, Smeed DA, Lankhorst M et al (2023) Meridional overturning circulation and heat transport in the Atlantic Ocean. In: Blunden J, Boyer T, Bartow-Gillies E (eds): State of the climate in 2022. Bulletin of the American Meteorological Society 9. vol 104, pp Si-S501. 10.1175/2023BAMSStateoftheClimate.1

[CR418] Visbeck M, Chassignet EP, Curry RG et al (2003) The ocean’s response to North Atlantic Oscillation variability. Geophys Monogr Am Geophys Union 134:113–146

[CR419] Vrac M, Vaittinada Ayar P, Yiou P (2014) Trends and variability of seasonal weather regimes. Int J Climatol 34:472–480. 10.1002/joc.3700

[CR420] Waite AJ, Klavans JM, Clement AC et al (2020) Observational and model evidence for an important role for volcanic forcing driving Atlantic multidecadal variability over the last 600 years. Geophys Res Lett 47(23):e2020GL089428

[CR421] Wang Q (2021) Stronger variability in the Arctic Ocean induced by sea ice decline in a warming climate: freshwater storage, dynamic sea level and surface circulation. J Geophys Res: Oceans 126:e2020JC016886. 10.1029/2020JC016886

[CR422] Wang Q, Wekerle C, Wang X et al (2020) Intensification of the Atlantic Water supply to the Arctic Ocean through Fram Strait induced by Arctic sea ice decline. Geophys Res Lett 47:e2019GL086682. 10.1029/2019GL086682

[CR423] Wang Q, Shu Q, Wang S et al (2023) A review of arctic-subarctic ocean linkages: past changes, mechanisms, and future projections. Ocean-Land-Atmos Res 2:0013. 10.34133/olar.0013

[CR425] Wang Z, Zhang M, Wang L, Qin W (2022) A comprehensive research on the global all-sky surface solar radiation and its driving factors during 1980–2019. Atmos Res 265:105870. 10.1016/j.atmosres.2021.105870

[CR426] Watanabe M, Tatebe H (2019) Reconciling roles of sulphate aerosol forcing and internal variability in Atlantic multidecadal climate changes. Clim Dyn 53(7):4651–4665

[CR427] Wei X, Zhang R (2022) A simple conceptual model for the self-sustained multidecadal AMOC variability. Geophys Res Lett 49(14):e2022GL099800

[CR428] Weijer W, Cheng W, Garuba OA, Hu A, Nadiga BT (2020) CMIP6 models predict significant 21st century decline of the Atlantic meridional overturning circulation. Geophys Res Lett 47(12):e2019GL086075

[CR429] Weisheimer A, Schaller N, O’Reilly C, MacLeod D, Palmer TN (2017) Atmospheric seasonal forecasts of the 20th Century: multi-decadal variability in predictive skill of the winter North Atlantic Oscillation and their potential value for extreme event attribution. Q J R Meteorol Soc 143:917–926. 10.1002/qj.2976 31413423 10.1002/qj.2976 PMC6686212

[CR430] Weisheimer A, Befort D, MacLeod D et al (2020) Seasonal forecasts of the 20th Century Bull. Amer Meteor Soc 101(8):E1413–E1426. 10.1175/BAMS-D-19-0019.1

[CR431] Weisheimer A, Baker LH, Broecker J et al (2024) The signal-to-noise paradox in climate forecasts: revisiting our understanding and identifying future priorities. Bull Am Meteor Soc. 10.1175/BAMS-D-24-0019.1

[CR432] Wett S, Rhein M, Kieke D, Mertens C, Moritz M (2023) Meridional connectivity of a 25-year observational AMOC record at 47° N. Geophys Res Lett 50(16):e2023GL103284

[CR433] Weyn JA, Durran DR, Caruana R, Cresswell-Clay N (2021) Sub-seasonal forecasting with a large ensemble of deep-learning weather prediction models. J Adv Model Earth Syst 13(7):e2021MS002502. 10.1029/2021ms002502

[CR434] Wilcox LJ, Allen RJ, Samset BH et al (2023) The Regional Aerosol Model Intercomparison Project (RAMIP). Geosci Model Dev 16(13):4451–4479. 10.5194/gmd-16-4451-2023

[CR435] Wild M (2016) Decadal changes in radiative fluxes at land and ocean surfaces and their relevance for global warming. Wiley Interdiscip Rev Clim Change 7:91–107. 10.1002/wcc.372

[CR436] Williams RG, Roussenov V, Smith D, Lozier MS (2014) Decadal evolution of ocean thermal anomalies in the North Atlantic: the effects of Ekman, overturning, and horizontal transport. J Clim 27(2):698–719

[CR437] Wills RC, Armour KC, Battisti DS, Hartmann DL (2019) Ocean–atmosphere dynamical coupling fundamental to the Atlantic multidecadal oscillation. J Clim 32(1):251–272

[CR438] Woollings T, Hannachi A, Hoskins B (2010) Variability of the North Atlantic eddy-driven jet stream. Q J R Meteorol Soc 136:856–868

[CR439] Woollings T, Blackburn M (2012) The North Atlantic jet stream under climate change and its relation to the NAO and EA patterns. J Clim 25:886–902

[CR440] Woollings T, Czuchnicki C, Franzke C (2014) Twentieth century North Atlantic jet variability. Q J Roy Meteor Soc 140:783–791. 10.1002/qj.2197

[CR441] Woollings T, Franzke C, Hodson DLR, Dong BW et al (2015) Contrasting interannual and multidecadal NAO variability. Clim Dyn 45:539–556

[CR442] Woollings T, Barnes E, Hoskins B et al (2018) Daily to decadal modulation of jet variability. J Clim. 10.1175/JCLI-D-17-0286.1

[CR443] Woollings T, Drouard M, O’Reilly CH et al (2023) Trends in the atmospheric jet streams are emerging in observations and could be linked to tropical warming. Commun Earth Environ 4:125. 10.1038/s43247-023-00792-8

[CR444] Wunsch C (1999) The interpretation of short climate records, with comments on the North Atlantic and Southern Oscillations. Bull Am Meteor Soc 80(2):245–256

[CR445] Yan X, Zhang R, Knutson TR (2018) Underestimated AMOC variability and implications for AMV and predictability in CMIP models. Geophys Res Lett 45(9):4319–4328. 10.1029/2018gl077378

[CR446] Yang H, Lohmann G, Krebs-Kanzow U et al (2020) Poleward shift of the major ocean gyres detected in a warming climate. Geophys Res Lett 47(5):e2019GL085868

[CR447] Ye A, Zhu Z, Zhang R, Xiao Z, Zhou L (2023a) Influence of solar forcing on multidecadal variability in the Atlantic meridional overturning circulation (AMOC). Front Earth Sci 11:1165386. 10.3389/feart.2023.1165386

[CR448] Ye K, Woollings T, Screen JA (2023b) European winter climate response to projected Arctic sea-ice loss strongly shaped by change in the North Atlantic jet. Geophys Res Lett 50:e2022102005. 10.1029/2022GL102005

[CR449] Yeager S (2020) The abyssal origins of North Atlantic decadal predictability. Clim Dyn 55(7):2253–2271

[CR450] Yeager S, Danabasoglu G (2014) The origins of late-twentieth-century variations in the large-scale North Atlantic circulation. J Clim 27:3222–3247. 10.1175/JCLI-D-13-00125.1

[CR451] Yeager SG, Robson JI (2017) Recent progress in understanding and predicting Atlantic decadal climate variability. Curr Clim Change Rep 3:112–127 32055436 10.1007/s40641-017-0064-z PMC6991968

[CR452] Yeager S, Karspeck A, Danabasoglu G, Tribbia J, Teng H (2012) A decadal prediction case study: late twentieth-century North Atlantic ocean heat content. J Clim 25:5173–5189. 10.1175/JCLI-D-11-00595.1

[CR453] Yeager SG, Karspeck AR, Danabasoglu G (2015) Predicted slowdown in the rate of Atlantic Sea ice loss. Geophys Res Lett 42:10704–10713. 10.1002/2015GL065364

[CR454] Yin JH (2005) A consistent poleward shift of the storm tracks in simulations of 21st century climate. Geophys Res Lett. 10.1029/2005GL023684

[CR455] Zanchettin D, Bothe O, Graf HF et al (2013) Background conditions influence the decadal climate response to strong volcanic eruptions. J Geophys Res Atmos 118(10):4090–4106

[CR456] Zanowski H, Jahn A, Holland MM (2021) Arctic Ocean freshwater in CMIP6 ensembles: declining sea ice, increasing ocean storage and export. J Geophys Res: Oceans 126(4):e2020JC016930

[CR457] Zappa G, Shepherd TG (2017) Storylines of atmospheric circulation change for european regional climate impact assessment. Am Met Soc. 10.1175/JCLI-D-16-0807.1

[CR458] Zappa G, Lucarini V, Navarra A (2011) Baroclinic stationary waves in aquaplanet models. J Atmos Sci 68(5):1023–1040

[CR459] Zappa G, Hoskins BJ, Shepherd TG (2015) Improving climate change detection through optimal seasonal averaging: the case of the North Atlantic jet and European precipitation. J Clim 28:6381–6397. 10.1175/JCLI-D-14-00823.1

[CR460] Zappa G, Pithan F, Shepherd TG (2018) Multimodel evidence for an atmospheric circulation response to arctic sea ice loss in the CMIP5 future projections. Geophys Res Lett 45(2):1011–1019 29576667 10.1002/2017GL076096 PMC5856070

[CR461] Zhang R (2008) Coherent surface-subsurface fingerprint of the Atlantic meridional overturning circulation. Geophys Res Lett 35:L20705. 10.1029/2008GL035463

[CR462] Zhang R, Sutton R, Danabasoglu G et al (2019) A review of the role of the Atlantic Meridional Overturning Circulation in Atlantic Multidecadal Variability and associated climate impacts. Rev Geophys 57:316–375. 10.1029/2019RG000644

[CR463] Zhang R, Vallis GK (2006) Impact of great salinity anomalies on the low-frequency variability of the North Atlantic climate. J Clim 19(3):470–482

[CR464] Zhang W, Kirtman B, Siqueira L, Clement A, Xia J (2021) Understanding the signal-to-noise paradox in decadal climate predictability from CMIP5 and an eddying global coupled model. Clim Dyn 56:2895–2913. 10.1007/s00382-020-05621-8

[CR465] Zhong Y, Miller GH, Otto-Bliesner BL et al (2011) Centennial-scale climate change from decadally-paced explosive volcanism: a coupled sea ice-ocean mechanism. Clim Dyn 37(11):2373–2387. 10.1007/s00382-010-0967-z

[CR466] Zhou W, Leung LR, Lu J (2022) Seasonally and regionally dependent shifts of the atmospheric westerly jets under global warming. J Clim 35(16):5433–5447

