# Supplementary material for: Key drivers of large scale changes in North Atlantic atmospheric and oceanic circulations and their predictability
Source: Clim Dyn. 2025 Feb 5;63(2):113. doi: 10.1007/s00382-025-07591-1 (PMC11882673; doi:10.1007/s00382-025-07591-1)
Supplement: Supplementary file 1 — Supplementary file1 (DOCX 1387 KB) [file 382_2025_7591_MOESM1_ESM.docx]

**Supplementary Information for the manuscript**

**Key drivers of large scale changes in North Atlantic atmospheric and oceanic circulations and their predictability**

Buwen Dong^1^*, Yevgeny Aksenov^2^, Ioana Colfescu^3^, Ben Harvey^1^, Joël Hirschi^2^, Simon Josey^2^, Hua Lu^4^, Jenny Mecking^2^, Marilena Oltmanns^2^, Scott Osprey^5^, Jon Robson^1^, Stefanie Rynders^2^, Len Shaffrey^1^, Bablu Sinha^2^, Rowan Sutton^1^, and Antje Weisheimer^5^

^1^National Centre for Atmospheric Science, Department of Meteorology, University of Reading, Reading, UK

^2^National Oceanography Centre, Southampton, UK

^3^University of St Andrews, St Andrews, UK

^4^British Antarctic Survey, Cambridge, UK

^5^Atmosphere, Ocean and Planetary Physics, University of Oxford, Oxford, UK

*Corresponding author address: Buwen Dong, National Centre for Atmospheric Science, Department of Meteorology, University of Reading, Reading, RG6 6BB. UK. E-mail: [b.dong@reading.ac.uk](mailto:b.dong@reading.ac.uk)

- Supplementary Figures S1-S4

**
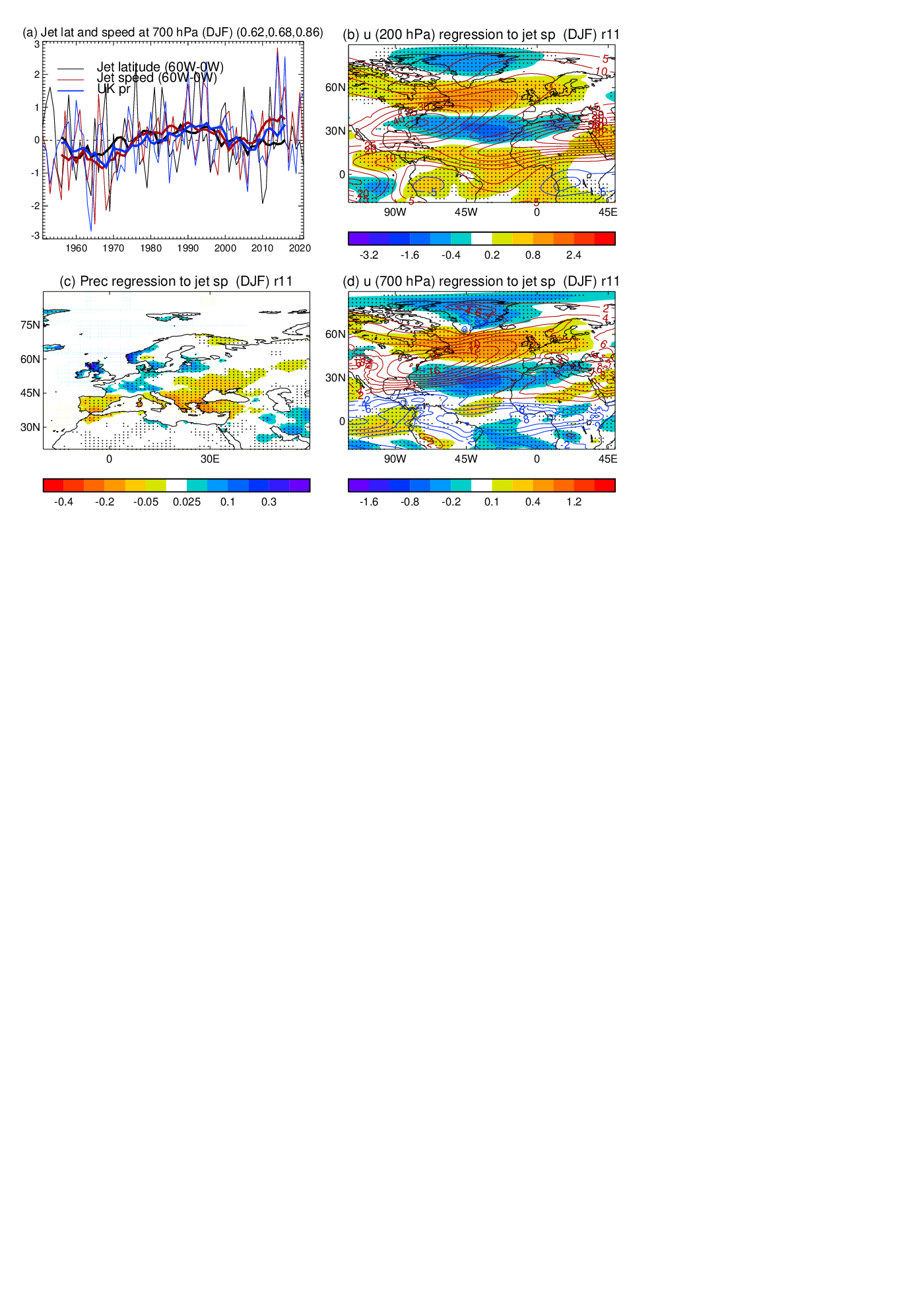
**

Supplementary Fig. S1: (a) Normalized (by standard deviation of interannual variability) time series of jet latitude, jet speed indices, based on ERA5 reanalysis (Hersbach et al. [2020](https://link.springer.com/article/10.1007/s00382-022-06438-3#ref-CR19)) at 700 hPa over the North Atlantic and UK precipitation index, based on CRUTS4.06 data set (Harris et al. 2014) in DJF with thick lines representing low frequency (11 year running mean) variations. Jet latitude and jet speed indices are defined as the latitude of maximum zonal wind averaged over (60^o^W-0) and the corresponding maximum zonal wind speed at 700 hPa. The UK precipitation index is defined as the area averaged precipitation over the land region (51-59^o^N, 6^o^W-0). The three numbers in the bracket of the panel (a) are corelation coefficients among the jet latitude, jet speed, and precipitation indices (e.g., jet latitude vs jet speed, jet latitude vs precipitation, and jet speed vs precipitation). (b, c, d) Spatial patterns of 200 hPa zonal wind, precipitation, and 700 hPa zonal wind in DJF regressed to the normalized low frequency variations of jet speed index in DJF. Contours (b and d) show climatology and dots (b, c, d) highlight regions where regressions are statistically significant at the 10% level based on the two-tailed Student’s t test.


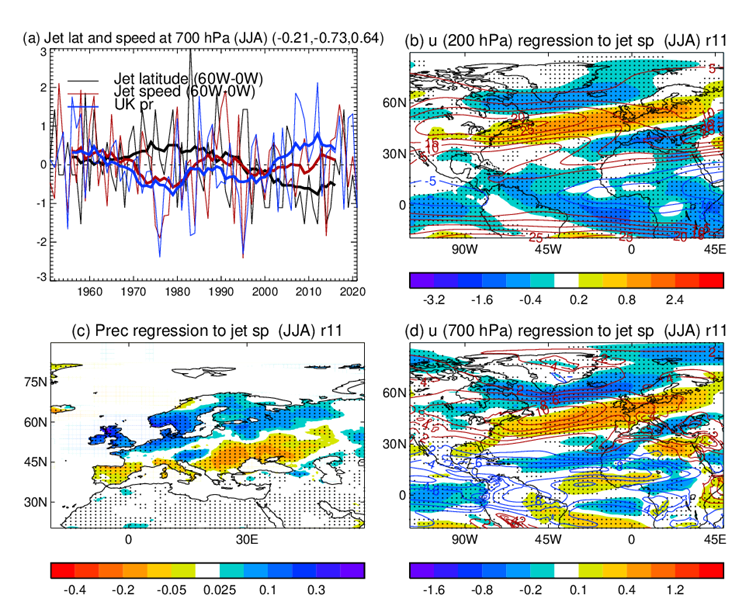


Supplementary Fig. S2: As Fig, S1 but for JJA.

**
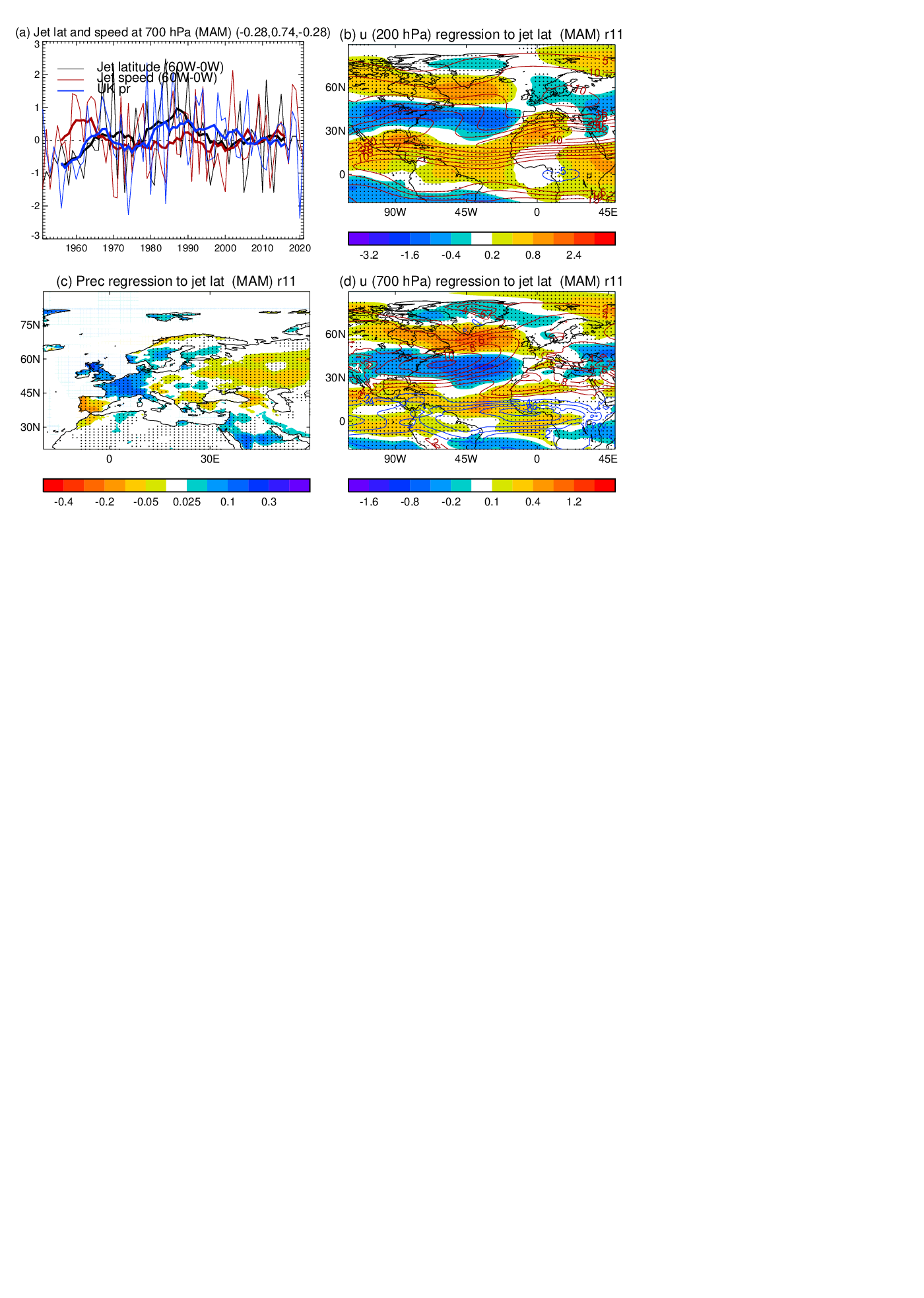
**

Supplementary Fig. S3: As Fig, S1 but for MAM. (b, c, d) Spatial patterns of 200 hPa zonal wind, precipitation, and 700 hPa zonal wind in MAM regressed to the normalized low frequency variations of jet speed in MAM.

**
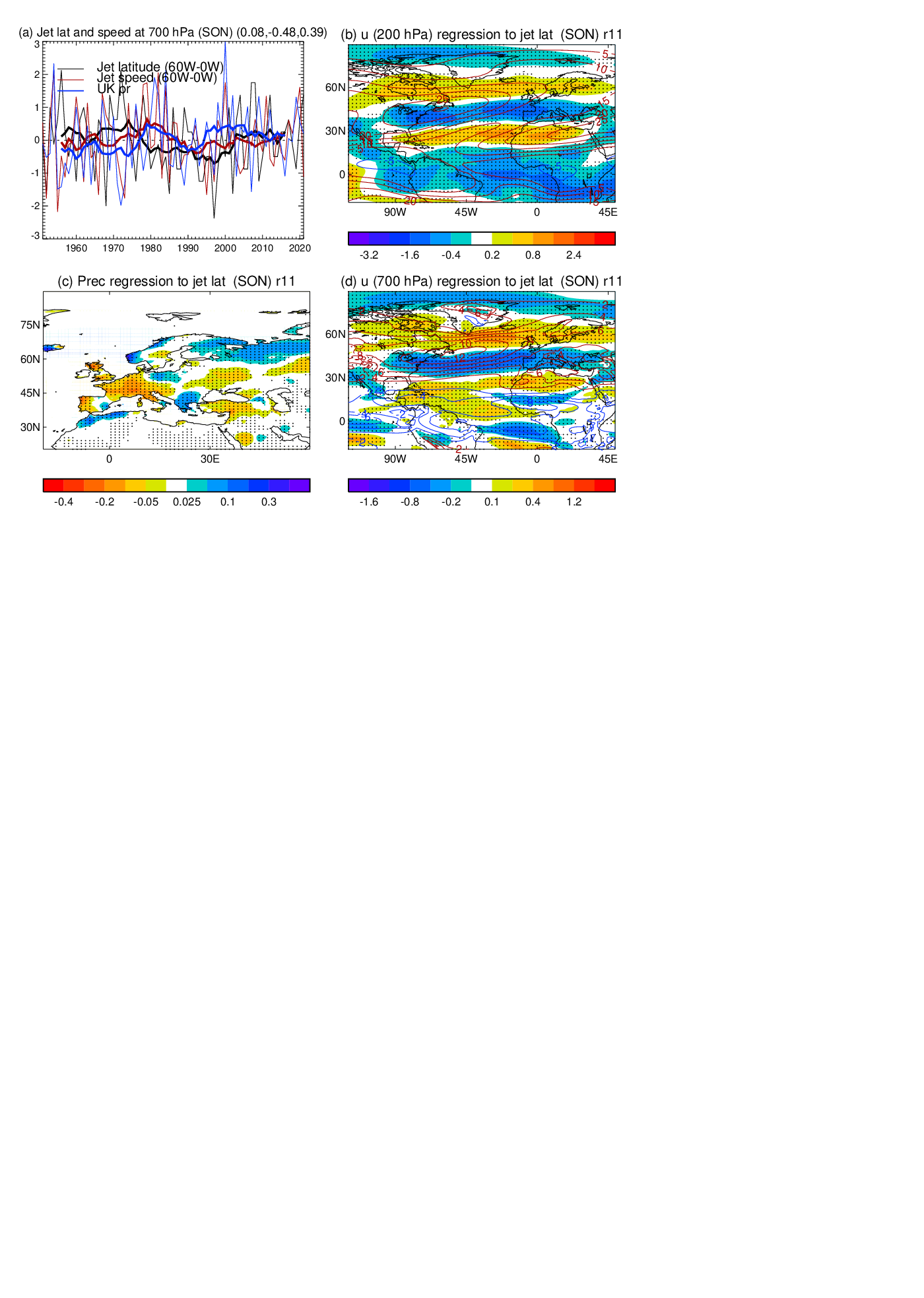
**

Supplementary Fig. S4: As Fig. S1 but for SON.
